# Waste Not, Want Not: Why Rarefying Microbiome Data Is Inadmissible

**DOI:** 10.1371/journal.pcbi.1003531

**Published:** 2014-04-03

**Authors:** Paul J. McMurdie, Susan Holmes

**Affiliations:** Statistics Department, Stanford University, Stanford, California, United States of America; Heinrich Heine University, Germany

## Abstract

Current practice in the normalization of microbiome count data is inefficient in the statistical sense. For apparently historical reasons, the common approach is either to use simple proportions (which does not address heteroscedasticity) or to use *rarefying* of counts, even though both of these approaches are inappropriate for detection of differentially abundant species. Well-established statistical theory is available that simultaneously accounts for library size differences and biological variability using an appropriate mixture model. Moreover, specific implementations for DNA sequencing read count data (based on a Negative Binomial model for instance) are already available in RNA-Seq focused R packages such as edgeR and DESeq. Here we summarize the supporting statistical theory and use simulations and empirical data to demonstrate substantial improvements provided by a relevant mixture model framework over simple proportions or rarefying. We show how both proportions and rarefied counts result in a high rate of false positives in tests for species that are differentially abundant across sample classes. Regarding microbiome sample-wise clustering, we also show that the rarefying procedure often discards samples that can be accurately clustered by alternative methods. We further compare different Negative Binomial methods with a recently-described zero-inflated Gaussian mixture, implemented in a package called *metagenomeSeq*. We find that metagenomeSeq performs well when there is an adequate number of biological replicates, but it nevertheless tends toward a higher false positive rate. Based on these results and well-established statistical theory, we advocate that investigators avoid rarefying altogether. We have provided microbiome-specific extensions to these tools in the R package, phyloseq.

## Author Summary

The term *microbiome* refers to the ecosystem of microbes that live in a defined environment. The decreasing cost and increasing speed of DNA sequencing technology has recently provided scientists with affordable and timely access to the genes and genomes of microbiomes that inhabit our planet and even our own bodies. In these investigations many microbiome samples are sequenced at the same time on the same DNA sequencing machine, but often result in total numbers of sequences per sample that are vastly different. The common procedure for addressing this difference in sequencing effort across samples – different *library sizes* – is to either (1) base analyses on the proportional abundance of each species in a library, or (2) *rarefy*, throw away sequences from the larger libraries so that all have the same, smallest size. We show that both of these *normalization methods* can work when comparing obviously-different whole microbiomes, but that neither method works well when comparing the relative proportions of each bacterial species across microbiome samples. We show that alternative methods based on a statistical *mixture model* perform much better and can be easily adapted from a separate biological sub-discipline, called RNA-Seq analysis.

This is a *PLOS Computational Biology* Methods article.

## Introduction

Modern, massively parallel DNA sequencing technologies have changed the scope and technique of investigations across many fields of biology [1], [2]. In gene expression studies the standard measurement technique has shifted away from microarray hybridization to direct sequencing of cDNA, a technique often referred to as *RNA-Seq* [3]. Analogously, culture independent [4] microbiome research has migrated away from detection of species through microarray hybridization of small subunit rRNA gene PCR amplicons [5] to direct sequencing of highly-variable regions of these amplicons [6], or even direct *shotgun* sequencing of microbiome metagenomic DNA [7]. Even though the statistical methods available for analyzing microarray data have matured to a high level of sophistication [8], these methods are not directly applicable because DNA sequencing data consists of discrete counts of sequence *reads* rather than continuous values derived from the fluorescence intensity of hybridized probes. In recent generation DNA sequencing the total reads per sample (*library size*; sometimes referred to as *depths of coverage*) can vary by orders of magnitude within a single sequencing run. Comparison across samples with different library sizes requires more than a simple linear or logarithmic scaling adjustment because it also implies different levels of uncertainty, as measured by the sampling variance of the proportion estimate for each feature (a feature is a gene in the RNA-Seq context, and is a species or Operational Taxonomic Unit, OTU, in the context of microbiome sequencing). In this article we are primarily concerned with optimal methods for addressing differences in library sizes from microbiome sequencing data.

Variation in the read counts of features between technical replicates have been adequately modeled by Poisson random variables [9]. However, we are usually interested in understanding the variation of features among biological replicates in order to make inferences that are relevant to the corresponding population; in which case a mixture model is necessary to account for the added uncertainty [10]. Taking a hierarchical model approach with the Gamma-Poisson has provided a satisfactory fit to RNA-Seq data [11], as well as a valid regression framework that leverages the power of generalized linear models [12]. A Gamma mixture of Poisson variables gives the negative binomial (NB) distribution [10], [11] and several RNA-Seq analysis packages now model the counts, *K*, for gene *i*, in sample *j* according to:
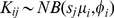
(1)where *s_j_* is a linear scaling factor for sample *j* that accounts for its library size, *μ_i_* is the mean proportion for gene *i*, and *φ_i_* is the dispersion parameter for gene *i*. The variance is 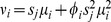, with the NB distribution becoming Poisson when *φ* = 0. Recognizing that *φ*>0 and estimating its value is necessary in gene-level tests in order to control the rate of false positive genes. Many false positive genes appear significantly differentially expressed between experimental conditions under the assumption of a Poisson distribution, but are nevertheless not-significant in tests that account for the larger variance that results from non-zero dispersion.

The uncertainty in estimating *φ_i_* for every gene when there is a small number of samples — or a small number of biological replicates — can be mitigated by sharing information across the thousands of genes in an experiment, leveraging a systematic trend in the mean-dispersion relationship [11]. This approach substantially increases the power to detect differences in proportions (differential expression) while still adequately controlling for false positives [13]. Many R packages implementing this model of RNA-Seq data are now available, differing mainly in their approach to modeling dispersion across genes [14]. Although DNA sequencing-based microbiome investigations use the same sequencing machines and represent the processed sequence data in the same manner — a feature-by-sample contingency table where the features are OTUs instead of genes — to our knowledge the modeling and normalization methods currently used in RNA-Seq analysis have not been transferred to microbiome research [15]–[17].

Instead, microbiome analysis workflows often begin with an *ad hoc* library size normalization by random subsampling without replacement, or so-called *rarefying* [17]–[19]. There is confusion in the literature regarding terminology, and sometimes this normalization approach is conflated with a non-parametric resampling technique — called *rarefaction* [20], or *individual-based taxon re-sampling curves* [21] — that can be justified for coverage analysis or species richness estimation in some settings [21], though in other settings it can perform worse than parametric methods [22]. Here we emphasize the distinction between taxon re-sampling curves and normalization by strictly adhering to the terms *rarefying* or *rarefied counts* when referring to the normalization procedure, respecting the original definition for *rarefaction*. Rarefying is most often defined by the following steps [18].

1. Select a minimum library size, 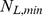. This has also been called the *rarefaction level* [17], though we will not use the term here.
2. Discard libraries (microbiome samples) that have fewer reads than 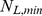.
3. Subsample the remaining libraries without replacement such that they all have size 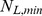.

Often 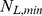 is chosen to be equal to the size of the smallest library that is not considered *defective*, and the process of identifying defective samples comes with a risk of subjectivity and bias. In many cases researchers have also failed to repeat the random subsampling step (3) or record the pseudorandom number generation seed/process — both of which are essential for reproducibility. To our knowledge, rarefying was first recommended for microbiome counts in order to moderate the sensitivity of the UniFrac distance [23] to library size, especially differences in the presence of rare OTUs [24]. In these and similar studies the principal objective is an exploratory/descriptive comparison of microbiome samples, often from different environmental/biological sources; a research task that is becoming increasingly accessible with declining sequencing costs and the ability to sequence many samples in parallel using barcoded primers [25], [26]. Rarefying is now an exceedingly common precursor to microbiome multivariate workflows that seek to relate sample covariates to sample-wise distance matrices [19], [27], [28]; for example, integrated as a recommended option in QIIME's [29] beta_diversity_through_plots.py workflow, in Sub.sample in the mothur software library [30], in daisychopper.pl [31], and is even supported in phyloseq's rarefy_even_depth function [32] (though not recommended in its documentation). The perception in the microbiome literature of “rarefying to even sampling depth” as a standard normalization procedure appears to explain why rarefied counts are also used in studies that attempt to detect *differential abundance* of OTUs between predefined classes of samples [33]–[37], in addition to studies that use proportions directly [38]. It should be noted that we have adopted the recently coined term *differential abundance* [39], [40] as a direct analogy to *differential expression* from RNA-Seq. Like differentially expressed genes, a species/OTU is considered differentially abundant if its mean proportion is significantly different between two or more sample classes in the experimental design.

### Statistical motivation

Despite its current popularity in microbiome analyses **rarefying biological count data is statistically inadmissible** because it requires the omission of available valid data. This holds even if repeated rarefying trials are compared for stability as previously suggested [17]. In this article we demonstrate the applicability of a variance stabilization technique based on a mixture model of microbiome count data. This approach simultaneously addresses both problems of (1) DNA sequencing libraries of widely different sizes, and (2) OTU (feature) count proportions that vary more than expected under a Poisson model. We utilize the most popular implementations of this approach currently used in RNA-Seq analysis, namely edgeR [41] and DESeq [13], adapted here for microbiome data. This approach allows valid comparison across OTUs while substantially improving both power and accuracy in the detection of differential abundance. We also compare the performance of the Gamma-Poisson mixture model against a method that models OTU proportions using a zero-inflated Gaussian distribution, implemented in a recently-released package called metagenomeSeq [40].

**Figure 1:**
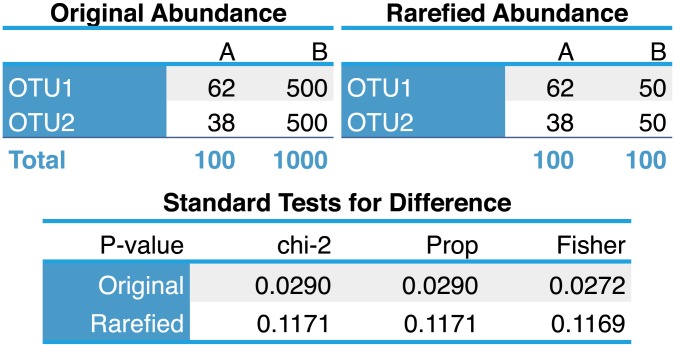
A minimal example of the effect of rarefying on statistical power. Hypothetical abundance data in its original (Top-Left) and rarefied (Top-Right) form, with corresponding formal test results for differentiation (Bottom).

A mathematical proof of the sub-optimality of the rarefying approach is presented in the supplementary material (Text S1). To help explain why rarefying is statistically inadmissible, especially with regards to variance stabilization, we start with the following minimal example. Suppose we want to compare two different samples, called *A* and *B*, comprised of 100 and 1000 DNA sequencing reads, respectively. In statistical terms, these library sizes are also equivalent to the number of trials in a sampling experiment. In practice, the library size associated with each biological sample is a random number generated by the technology, often varying from hundreds to millions. For our example, we imagine the simplest possible case where the samples can only contain two types of microbes, called *OTU1* and *OTU2*. The results of this hypothetical experiment are represented in the *Original Abundance* section of Figure 1. Formally comparing the two proportions according to a standard test could technically be done either using a *χ* ^2^ test (equivalent to a two sample proportion test here) or a Fisher exact test. By first rarefying (Figure 1, *Rarefied Abundance* section) so that both samples have the same library size before doing the tests, we are no longer able to differentiate the samples (Figure 1, tests). This loss of power is completely attributable to reducing the size of *B* by a factor of 10, which also increases the width of the confidence intervals corresponding to each proportion such that they are no longer distinguishable from those in *A* even though they are distinguishable in the original data.

The variance of the proportion's estimate 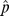 is multiplied by 10 when the total count is divided by 10. In this binomial example the variance of the proportion estimate is 
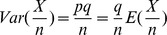
, a function of the mean. This is a common occurrence and one that is traditionally dealt with in statistics by applying variance-stabilizing transformations. We show in Text S1 that the relation between the variance and the mean for microbiome count data can be estimated and the model used to find the optimal variance-stabilizing transformation. As illustrated by this simple example, it is inappropriate to compare the proportions of OTU *i*, 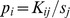, without accounting for differences in the denominator value (the library size, *s_j_*) because they have unequal variances. This problem of unequal variances is called *heteroscedasticity*. In other words, the *uncertainty* associated with each value in the table is fundamentally linked to the total number of observations (or reads), which can vary even more widely than a 10-fold difference. In practice we will be observing hundreds of different OTUs instead of two, often with dependendency between the counts. Nevertheless, the difficulty caused by unequal library sizes still pertains.

The uncertainty with which each proportion is estimated must be considered when testing for a difference between proportions (one OTU), or sets of proportions (a microbial community). Although rarefying does equalize variances, it does so only by inflating the variances in all samples to the largest (worst) value among them at the cost of discriminating power (increased uncertainty). Rarefying also adds artificial uncertainty through the random subsampling step, such that Figure 1 shows the best-case, achieved only with a sufficient number of *repeated* rarefying trials (See Protocol S1, minimal example). In this sense alone, the random step in rarefying is unnecessary. Each count value could be transformed to a *common-scale* by rounding 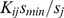. Although this common-scale approach is an improvement over the rarefying method here defined, both methods suffer from the same problems related to lost data.

## Methods

**Figure 2:**
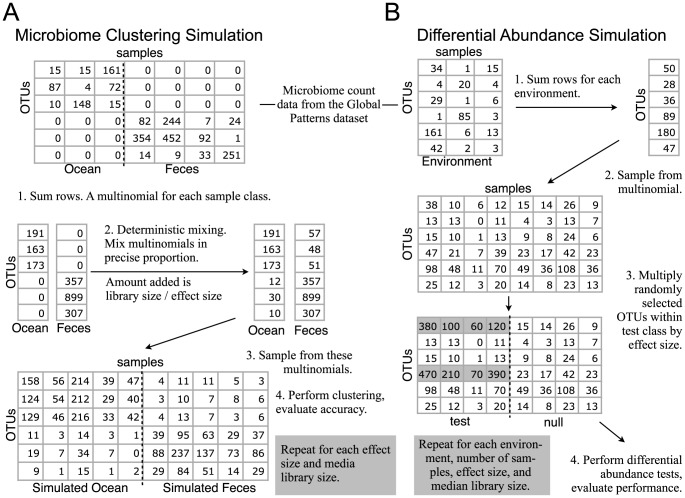
Graphical summary of the two simulation frameworks. Both *Simulation A* (clustering) and *Simulation B* (differential abundance) are represented. All simulations begin with real microbiome count data from a survey experiment referred to here as “the Global Patterns dataset” [48]. Tables of integers with multiple columns represent an abundance count matrix (“OTU table”), while a single-column of integers represents a multinomial of OTU counts/proportions. In both simulation illustrations an *effect size* is explained and given an example value of 10 for easy mental computation, but its meaning is different for each simulation. Note that *effect size* is altogether different than *library size*, the latter being equivalent to both the column sums and the number of reads per sample. A grey highlight indicates count values for which an effect has been applied in *Simulation B*. Protocol S1 includes the complete source code used to compute the example values shown here, as well as the full simulations discussed below.

In order to quantify the relative statistical costs of rarefying, and to illustrate the relative benefits of an appropriate mixture model, we created two microbiome simulation workflows based on repeated subsampling from empirical data. These workflows were organized according to Figure 2. Because the correct answer in every simulation is known, we were able to evaluate the resulting power and accuracy of each statistical method, and thus quantify the improvements one method provided over another under a given set of conditions. In both simulation types we varied the library size and effect size across a range of values that are relevant for recently-published microbiome investigations, and followed with commonly used statistical analyses from the microbiome and/or RNA-Seq literature.

### Simulation A

Simulation A is a simple example of a descriptive experiment in which the main goal is to distinguish patterns of relationships between whole microbiome samples through normalization followed by the calculation of sample-wise distances. Many early microbiome investigations are variants of Simulation A, and also used rarefying prior to calculating UniFrac distances [27]. Microbiome studies often graphically represent the results of their pairwise sample distances using multidimensional scaling [42] (also called *Principal Coordinate Analysis*, PCoA), which is useful if the desired effects are clearly evident among the first two or three ordination axes. In some cases, formal testing of sample covariates is also done using a permutation MANOVA (e.g. vegan::adonis in R [43]) with the (squared) distances and covariates as response and linear predictors, respectively [44]. However, in this case we are not interested in creating summary graphics or testing the explanatory power of sample covariates, but rather we are interested in precisely evaluating the relative discriminating capability of each combination of normalization method and distance measure. We will use clustering results as a quantitative proxy for the broad spectrum of approaches taken to interpret microbiome sample distances.

#### Normalizations in Simulation A

For each simulated experiment we used the following normalization methods prior to calculating sample-wise distances.

1. **DESeqVS**. Variance Stabilization implemented in the DESeq package [13].
2. **None**. Counts not transformed. Differences in total library size could affect the values of some distance metrics.
3. **Proportion**. Counts are divided by total library size.
4. **Rarefy**. Rarefying is performed as defined in the introduction, using rarefy_even_depth implemented in the phyloseq package [32], with 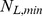 set to the 15*^th^*-percentile of library sizes within each simulated experiment.
5. **UQ-logFC**. The *Upper-Quartile Log-Fold Change* normalization implemented in the edgeR package [41], coupled with the *top-MSD* distance (see below).

#### Distances in Simulation A

For each of the previous normalizations we calculated sample-wise distance/dissimilarity matrices using the following methods, if applicable.

1. **Bray-Curtis**. The Bray-Curtis dissimilarity first defined in 1957 for forest ecology [45].
2. **Euclidean**. The euclidean distance treating each OTU as a dimension. This has the form 
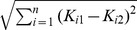
, for the distance between samples 1 and 2, with *K* and *i* as defined in the Introduction and *n* the number of distinct OTUs.
3. **PoissonDist**. Our abbreviation of PoissonDistance, a sample-wise distance implemented in the PoiClaClu package [46].
4. **top-MSD**. The mean squared difference of top OTUs, as implemented in edgeR [41].
5. **UniFrac-u**. The Unweighted UniFrac distance [23].
6. **UniFrac-w**. The Weighted UniFrac distance [47].

In order to consistently evaluate performance in this regard, we generated microbiome counts by sampling from two different multinomials that were based on either the *Ocean* or *Feces* microbiomes of the *Global Patterns* empirical dataset [48]. An equal number of simulated microbiome samples was generated from each multinomial for each simulated experiment. The *Ocean* and *Feces* sample classes were chosen because they have negligible overlapping OTUs, and mixing them by a defined proportion allows arbitrary control over the difficulty of the subsequent sample clustering task from trivial (no mixing) to impossible (both multinomials evenly mixed). See Figure 2 for a simplified example of a single simulated experiment, generated using a small subset of the empirical data with corresponding simplified code provided in the *simulation-design* section of Protocol S1.

Clustering was performed independently for each combination of simulated experiment, normalization method, and distance measure using partitioning around medoids (PAM [49], [50], an alternative to k-means that is considered more robust) with the number of clusters fixed at two. The accuracy in the clustering results was defined as the fraction of simulated samples correctly clustered; with the worst possible accuracy being 50% if all samples are clustered. Note that the rarefying procedure omits samples, so its accuracy can be below 50% under this definition. Alternative clustering methods, hierarchical clustering and k-means, were also performed and their results are included in Protocol S1.

The number of samples (40) to include for each template in Simulation A was chosen arbitrarily after some exploration of preliminary simulations. It was apparent that the classification results from Simulation A were most informative when we included enough samples per simulated experiment to achieve stable results, but not so many that it was experimentally unrealistic and prohibitively slow to compute. Conversely, preliminary trials of Simulation A that included only a few samples per experiment resulted in a large variance on each performance measure that was difficult to interpret.

### Simulation B

Simulation B is a simple example of microbiome experiments in which the goal is to detect microbes that are differentially abundant between two pre-determined classes of samples. This experimental design appears in many clinical settings (health/disease, target/control, etc.), and other settings for which there is sufficient *a priori* knowledge about the microbiological conditions, and we want to enumerate the OTUs that are different between these microbiomes, along with a measure of confidence that the proportions differ. For this form of analysis, the microbiome counts for each simulated experiment are generated by sampling from a single multinomial derived from the OTU proportions observed in one environment of the Global Patterns dataset. To create an effect, the simulated samples of an experiment were divided into two equally-sized classes, test and null, and a perturbation was applied (multiplication by a defined value) to the count values of a random subset of OTUs in the test class only. See part B of Figure 2 for a simple example. Each of the randomly perturbed OTUs is differentially abundant between the classes, and the performance of downstream tests can be evaluated on how well these OTUs are detected without falsely selecting OTUs for which no perturbation occurred (false positives). False negatives are perturbed OTUs that went undetected. This approach for generating simulated experiments with a defined effect size (in the form of multiplicative factor) was repeated for each combination of median library size, number of samples per class, and the nine microbial environments included in the Global Patterns dataset. Each simulated experiment was subjected to various approaches for normalization/noise-modeling and differential abundance testing.

#### Normalization/Modeling in Simulation B

For each simulated experiment, we used the following normalization/modeling methods prior to testing for differential abundance.

1. **Model/None**. A parametric model was applied to the data, or, in the case of the t-test, no normalization was applied (note: the t-test without normalization can only work with a high degree of balance between classes, and is provided here for comparison but is not recommended in general).
2. **Rarefied**. Rarefying is performed as defined in the introduction, using rarefy_even_depth implemented in the phyloseq package [32], with 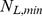 set to the 15*^th^*-percentile of library sizes within each simulated experiment.
3. **Proportion**. Counts are divided by total library size.

#### Testing in Simulation B

For each OTU of each simulated experiment we used the following to test for differential abundance.

1. **two sided Welch t-test**. A two-sided t-test with unequal variances, using the mt wrapper in phyloseq [32] of the mt.maxT method in the multtest package [51].
2. **edgeR - exactTest**. An exact binomial test (see base R's stats::binom.test) generalized for overdispersed counts [11] and implemented in the exactTest method of the edgeR package [41].
3. **DESeq - nbinomTest**. A Negative Binomial conditioned test similar to the edgeR test above, implemented in the nbinomTest method of the DESeq package [13]. See the subsection *Testing for differential expression* in Anders and Huber, 2010 [13] for the precise definition.
4. **DESeq2 - nbinomWaldTest**. A Negative Binomial Wald Test using standard maximum likelihood estimates for GLM coefficients assuming a zero-mean normal prior distribution, implemented in the nbinomWaldTest method of the DESeq2 package.
5. **metagenomeSeq - fitZig**. An Expectation-Maximization estimate of the posterior probabilities of differential abundance based on a *Zero Inflated Gaussian* model, implemented in the fitZig method of the metagenomeSeq package [40].

All tests were corrected for multiple inferences using the Benjamini-Hochberg method to control the False Discovery Rate [52]. Please note that in the context of these simulations *library size* is altogether different from *effect size*; the former being equivalent to both the column sums and the number of reads per sample. The library sizes for both categories of simulation were randomly sampled from the original distribution of library sizes in the Global Patterns dataset, and then scaled according to the prescribed median library size of each simulated experiment.

We have included in Protocol S1 the complete source code for computing the survey, simulations, normalizations, and performance assessments described in this article. This includes the code to acquire publicly available data via the phyloseq interface to the microbio.me/qiime server, a function called microbio_me_qiime [32]. Where applicable, this code includes the RNG seed so that the simulations and random resampling procedures can be reproduced *exactly*. Interested investigators can inspect and modify this code, change the random seed and other parameters, and observe the results (including figures). For ease of inspection, we have authored the source code in *R flavored markdown* [53], through which we have generated HTML5 files for each simulation that include our extensive comments interleaved with code, results, and both intermediate and final figures. Our simulation output can be optionally-modified and re-executed using the the knit2html function in the knitr package. This function will take the location of the simulation source files as input, evaluate its R code in sequence, generate graphics and markdown, and produce the complete HTML5 output file that can be viewed in any modern web browser. These simulations, analyses, and graphics rely upon the cluster [54], foreach [55], ggplot2 [56], metagenomeSeq [40], phyloseq [32], plyr [57], reshape2 [58], and ROCR [59] R packages; in addition to the DESeq(2) [13], edgeR [41], and PoiClaClu [46] R packages for RNA-Seq data, and tools available in the standard R distribution [60]. The Global Patterns [48] dataset included in phyloseq was used as empirical microbiome template data for simulations.

## Results/Discussion

**Figure 3:**
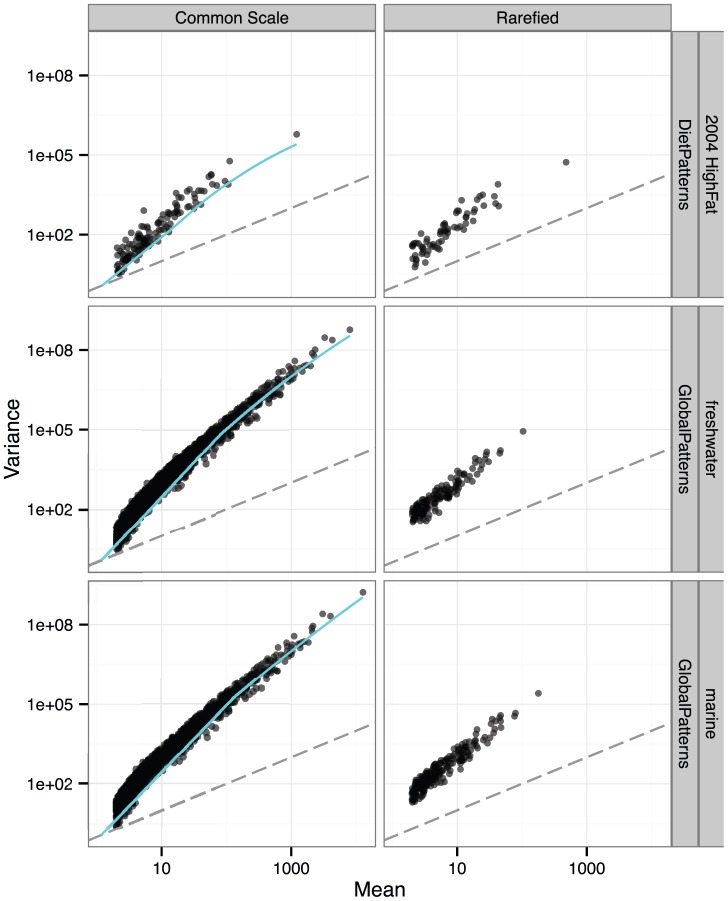
Examples of overdispersion in microbiome data. Common-Scale Variance versus Mean for Microbiome Data. Each point in each panel represents a different OTU's mean/variance estimate for a biological replicate and study. The data in this figure come from the *Global Patterns* survey [48] and the *Long-Term Dietary Patterns* study [75], with results from additional studies included in Protocol S1. (Right) Variance versus mean abundance for rarefied counts. (Left) Common-scale variances and common-scale means, estimated according to Equations 6 and 7 from Anders and Huber [13], implemented in the DESeq package (Text S1). The dashed gray line denotes the *σ* ^2^ = *μ* case (Poisson; *φ* = 0). The cyan curve denotes the fitted variance estimate using DESeq [13], with method = ‘pooled’, sharingMode = ‘fit-only’, fitType = ‘local’.

We surveyed various publicly available microbiome count data to evaluate the variance-mean relationship for OTUs among sets of biological replicates, a few examples of which are shown here (Figure 3). In every instance the variances were larger than could be expected under a Poisson model (overdispersed, *φ*>0), especially at larger values of the common-scale mean. By definition, these OTUs are the most abundant, and receive the greatest interest in many studies. For rarefied counts the absolute scales are decreased and there are many fewer OTUs that pass filtering, but overdispersion is present in both cases and follows a clear sample-wide trend. See the *dispersion-survey* section of Protocol S1 for additional examples of overdispersed microbiome counts. The consistent (though non-linear) relationship between variance and mean indicates that parameters of a NB model, especially *φ_i_*, can be adequately estimated among biological replicates of microbiome data, despite a previous weak assertion to the contrary [39].

**Figure 4:**
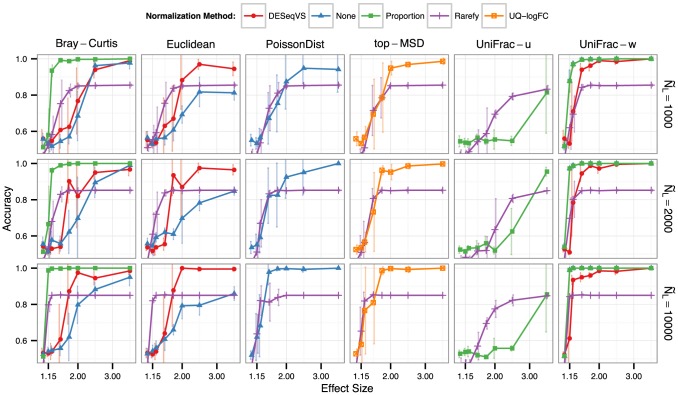
Clustering accuracy in simulated two-class mixing. *Partitioning around medoids* [49], [50] clustering accuracy (vertical axis) that results following different normalization and distance methods. Points denote the mean values of replicates, with a vertical bar representing one standard deviation above and below. Normalization method is indicated by both shade and shape, while panel columns and panel rows indicate the distance metric and median library size (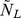), respectively. The horizontal axis is the effect size, which in this context is the ratio of target to non-target values in the multinomials that were used to simulate microbiome counts. Each multinomial is derived from two microbiomes that have negligible overlapping OTUs (Fecal and Ocean microbiomes in the Global Patterns dataset [48]). Higher values of effect size indicate an easier clustering task. For simulation details and precise definitions of abbreviations see *Simulation A* of the Methods section.

**Figure 5:**
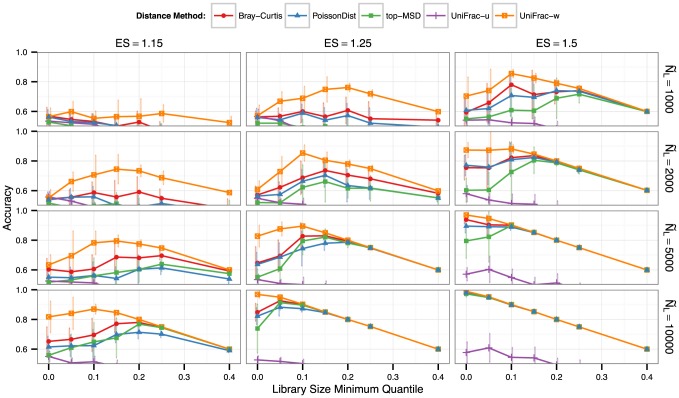
Normalization by rarefying only, dependency on library size threshold. Unlike the analytical methods represented in Figure 4, here rarefying is the only normalization method used, but at varying values of the minimum library size threshold, shown as library-size quantile (horizontal axis). Panel columns, panel rows, and point/line shading indicate effect size (ES), median library size (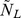), and distance method applied after rarefying, respectively. Because discarded samples cannot be accurately clustered, the line 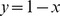 is the maximum achievable accuracy.

In simulations evaluating clustering accuracy, we found that rarefying undermined the performance of downstream clustering methods. This was the result of omitted read counts, added noise from the random sampling step in rarefying, as well as omitted microbiome samples with small library sizes that were accurately clustered by alternative procedures on the same simulated data (Figure 4). The extent to which the rarefying procedure performed worse depended on the effect-size (ease of clustering), as well as the typical library size of the samples in the simulation and the choice of threshold for the minimum library size (Figure 5). We also evaluated the performance of alternative clustering methods, k-means and hierarchical clustering, on the same tasks and found similar overall results (Protocol S1).

In additional rarefying simulations we investigated the dependency of clustering performance on the choice of minimum library threshold, 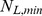. We found that samples were trivial to cluster for the largest library sizes using most distance methods, even with the threshold set to the smallest library in the simulation (no samples discarded, all correctly clustered). However, at more modest library sizes typical of highly-parallel experimental designs the optimum choice of size threshold is less clear. A small threshold implies retaining more samples but with a smaller number of reads (less information) per sample; whereas a larger threshold implies more discarded samples, but with larger libraries for the samples that remain. In our simulations the optimum choice of threshold hovered around the 15*^th^*-percentile of library sizes for most simulations and normalization/distance procedures (Figure 5), but this value is not generalizable to other data. Regions within Figure 5 in which all distances have converged to the same line (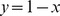) are regions for which the minimum library threshold completely controls clustering accuracy (all samples not discarded are accurately clustered). Regions to the left of this convergence indicate a compromise between discarding fewer samples and retaining enough counts per sample for accurate clustering.

**Figure 6:**
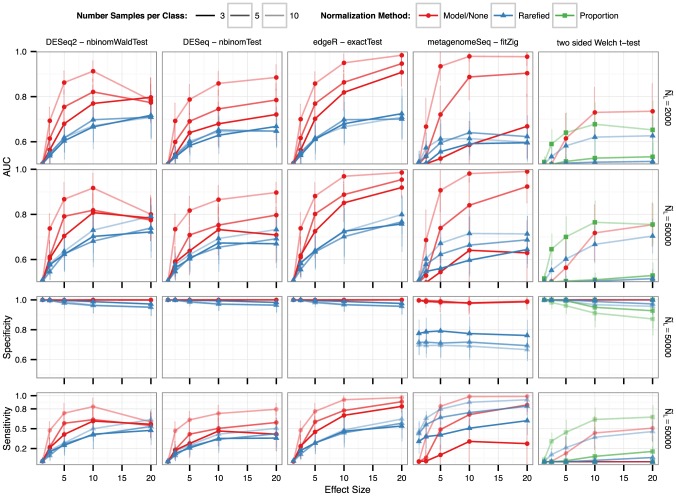
Performance of differential abundance detection with and without rarefying. Performance summarized here by the “Area Under the Curve” (AUC) metric of a Receiver Operator Curve (ROC) [59] (vertical axis). Briefly, the AUC value varies from 0.5 (random) to 1.0 (perfect), incorporating both sensitivity and specificity. The horizontal axis indicates the effect size, shown as the actual multiplication factor applied to OTU counts in the test class to simulate a differential abundance. Each curve traces the respective normalization method's mean performance of that panel, with a vertical bar indicating a standard deviation in performance across all replicates and microbiome templates. The right-hand side of the panel rows indicates the median library size, 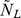, while the darkness of line shading indicates the number of samples per simulated experiment. Color shade and shape indicate the normalization method. See Methods section for the definitions of each normalization and testing method. For all methods, detection among multiple tests was defined using a False Discovery Rate (Benjamini-Hochberg [52]) significance threshold of 0.05.

In simulations evaluating performance in the detection of differential abundance, we found an improvement in sensitivity and specificity when normalization and subsequent tests are based upon a relevant mixture model (Figure 6). Multiple t-tests with correction for multiple inference did not perform well on this data, whether on rarefied counts or on proportions. A direct comparison of the performance of more sophisticated parametric methods applied to both original and rarefied counts demonstrates the strong potential of these methods and large improvements in sensitivity and specificity if rarefying is not used at all.

In general, the rate of false positives from tests based on proportions or rarefied counts was unacceptably high, and increased with the effect size. This is an undesirable phenomenon in which the increased relative abundance of the true-positive OTUs (the effect) is large enough that the null (unmodified) OTUs appear significantly more abundant in the null samples than in the test samples. This explanation is easily verified by the sign of the test statistics of the false positive OTU abundances, which was uniformly positive (Protocol S1). Importantly, this *side-effect* of a strong differential abundance was observed rarely in edgeR performance results under TMM normalization (not shown) but not with RLE normalization (shown), and was similarly absent in DESeq(2) results. The false positive rate for edgeR and DESeq(2) was near zero under most conditions, with no obvious correlation between false positive rate and effect size. Although rarefied counts and proportions both performed relatively poorly, count proportions outperformed rarefied counts in most simulations due to better sensitivity, but also suffered from a higher rate of false positives at larger values of effect size (Figure 6, Protocol S1).

The rarefying normalization procedure was associated with performance costs in both sample-clustering and differential abundance statistical evaluations, enumerated in the following.

1. Rarefied counts represent only a small fraction of the original data, implying an increase in Type-II error – often referred to as *a loss of power* or *decreased sensitivity* (Figure 1). In sample-wise comparisons, this lost power is evident through two separate phenomena, (1) samples that cannot be classified because they were discarded, (2) samples that are poorly distinguishable because of the discarded fraction of the original library (Figure 5). Differential abundance analyses that include moderate to rare OTUs are even more sensitive to this loss of power, where rarefied counts perform worse in every analysis method we attempted (Figure 6, Protocol S1).
2. Rarefied counts remain overdispersed relative to a Poisson model, implying an increase in Type-I error (decreased specificity). Overdispersion is theoretically expected for counts of this nature, and we unambiguously detected overdispersion in our survey of publicly available microbiome counts (Figure 3). Estimating overdispersion is also more difficult after rarefying because of the lost information (Figure 6). In our simulations, Type-I error was much worse for rarefied counts than original counts (Figure 6, Protocol S1).
3. Rarefying counts requires an arbitrary selection of a library size minimum threshold that affects downstream inference (Figure 5), but for which an optimal value cannot be known for new empirical data [17].
4. The random step in rarefying is unnecessary and adds artificial uncertainty (Protocol S1, minimal example, bottom). A superior transformation (though still inadmissible) is to instead round the expected value of each count at the new smaller library size, that is 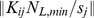, avoiding the additional sampling error as well as the need to repeat the random step [24] and publish the random seed/process.

Due to these demonstrated limitations and proven sub-optimality, we advocate that **rarefying should not be used**. In special cases the costs listed above may be acceptable for sample-comparison experiments in which the effect-size(s) and the original library sizes are large enough to withstand the loss of data. Many early descriptive studies fall into this category – for example comparing functionally distinct human body sites or environments [48] – and the ability to accurately distinguish those vastly-different microbiome samples is not in question, even with rarefied counts. However, for new empirical data the effect size(s) are unknown and may be subtle; and consequently, rarefying may undermine downstream analyses.

In the case of differential abundance detection, it seems unlikely that the cost of rarefying is ever acceptable. In our simulations, both rarefied counts and sample proportions resulted in an unacceptably high rate of false positive OTUs. As we described theoretically in the introduction, this is explained by differences among biological replicates that manifest as overdispersion, leading to a subsequent underestimate of the true variance if a relevant mixture model is not used. We detected overdispersion among biological replicates in all publicly available microbiome count datasets that we surveyed (Figure 3, Protocol S1). Failure to account for this overdispersion – by using proportions or rarefied counts – results in a systematic bias that increases the Type-I error rate even after correcting for multiple-hypotheses (*e.g.* Benjamini-Hochberg [52]). In other words, if overdispersion has not been addressed, we predict many of the reported differentially abundant OTUs are false positives attributable to an underestimate of uncertainty.

In our simulations this propensity for Type-I error *increased* with the effect size, *e.g.* the fold-change in OTU abundance among the true-positive OTUs. For rarefied counts, we also detected a simultaneous increase in Type-II error attributable to the forfeited data. It may be tempting to imagine that the increased variance estimate due to rarefying could be counterbalanced by the variance underestimate that results from omitting a relevant mixture model. However, such a scenario constitutes an unlikely special case, and false positives will not compensate for the false negatives in general. In our simulations both Type-I and Type-II error increased for rarefied counts (Figure 6, Protocol S1).

Fortunately, we have demonstrated that strongly-performing alternative methods for normalization and inference are already available. In particular, an analysis that models counts with the Negative Binomial – as implemented in DESeq2 [13] or in edgeR [41] with RLE normalization – was able to accurately and specifically detect differential abundance over the full range of effect sizes, replicate numbers, and library sizes that we simulated (Figure 6). DESeq-based analyses are routinely applied to more complex tests and experimental designs using the generalized linear model interface in R [61], and so are not limited to a simple two-class design. We also verified an improvement in differential abundance performance over rarefied counts or proportions by using an alternative mixture model based on the zero-inflated Gaussian, as implemented in the metagenomeSeq package [40]. However, we did not find that metagenomeSeq's AUC values were uniformly highest, as Negative Binomial methods had higher AUC values when biological replicate samples were low. Furthermore, while metagenomeSeq's AUC values were marginally higher than Negative Binomial methods at larger numbers of biological replicates, this was generally accompanied with a much higher rate of false positives (Figure 6, Protocol S1).

Based on our simulation results and the widely enjoyed success for highly similar RNA-Seq data, we recommend using DESeq2 or edgeR to perform analysis of differential abundance in microbiome experiments. It should be noted that we did not comprehensively explore all available RNA-Seq analysis methods, which is an active area of research. Comparisons of many of these methods on empirical [62], [63] and simulated [14], [64], [65] data find consistently effective performance for detection of differential expression. One minor exception is an increased Type-I error for edgeR compared to later methods [62], which was also detected in our results relative to DESeq and DESeq2 when TMM normalization was used (not shown) – but not after switching to RLE normalization (Figure 6, Protocol S1). Generally speaking, the reported performance improvements between these methods are incremental relative to the large gains attributable to applying a relevant mixture model of the noise with shared-strength across OTUs. However, some of these alternatives from the RNA-Seq community may outperform DESeq2 on microbiome data meeting special conditions, for example a large proportion of true positives and sufficient replicates [66], small sample sizes [14], or extreme values [67].

Although we did not explore the topic in the simulations here described, a procedure for further improving differential expression detection performance, called *Independent Filtering* [68], also applies to microbial differential abundance. Some heuristics for filtering low-abundance OTUs are already described in the documentation of various microbiome analysis workflows [29], [30], and in many cases these can be classified as forms of Independent Filtering. More effort is needed to optimize Independent Filtering for differential abundance detection, and rigorously define the theoretical basis and heuristics applicable to microbiome data. Ideally a formal application of Independent Filtering of OTUs would replace many of the current *ad hoc* approaches that often include poor reproducibility, poor justification, and the opportunity to introduce bias.

Some of the justification for the rarefying procedure has originated from exploratory sample-wise comparisons of microbiomes for which it was observed that a larger library size also results in additional observations of rare species, leading to a library size dependent increase in estimates of both alpha- and beta-diversity [24], [69], especially UniFrac [70]. It should be emphasized that this represents a failure of the implementation of these methods to properly account for rare species and not evidence that diversity depends on library size. Rarefying is far from the optimal method for addressing rare species, even when analysis is restricted solely to sample-wise comparisons. As we demonstrate here, it is more data-efficient to model the noise and address extra species using statistical normalization methods based on variance stabilization and robustification/filtering. Though beyond the scope of this work, a Bayesian approach to species abundance estimation would allow the inclusion of pseudo-counts from a Dirichlet prior that should also substantially increase robustness to rare species.

Our results have substantial implications for past and future microbiome analyses, particularly regarding the interpretation of differential abundance. Most microbiome studies utilizing high-throughput DNA sequencing to acquire culture-independent counts of species/OTUs have used either proportions or rarefied counts to address widely varying library sizes. Left alone, both of these approaches suffer from a failure to address overdispersion among biological replicates, with rarefied counts also suffering from a loss of power, and proportions failing to account for heteroscedasticity. Previous reports of differential abundance based on rarefied counts or proportions bear a strong risk of bias toward false positives, and may warrant re-evaluation. Current and future investigations into microbial differential abundance should instead model uncertainty using a hierarchical mixture, such as the Poisson-Gamma or Binomial-Beta models, and normalization should be done using the relevant variance-stabilizing transformations. This can easily be put into practice using powerful implementations in R, like DESeq2 and edgeR, that performed well on our simulated microbiome data. We have provided wrappers for edgeR, DESeq, DESeq2, and metagenomeSeq that are tailored for microbiome count data and can take common microbiome file formats through the relevant interfaces in the phyloseq package [32]. These wrappers are included with the complete code and documentation necessary to exactly reproduce the simulations, analyses, surveys, and examples shown here, including all figures (Protocol S1). This example of fully reproducible research can and should be applied to future publication of microbiome analyses [71]–[73].

## Supporting Information

Protocol S1 **A zip file containing all supplementary source files.** This includes the Rmd source code, HTML output, and all related documentation and code to completely and exactly recreate every results figure in this article.
(ZIP)

Text S1 **A supplemental appendix of the statistical mathematics described in the article.**
(PDF)
