# Supplementary material for: Waste Not, Want Not: Why Rarefying Microbiome Data Is Inadmissible
Source: PLoS Comput Biol. 2014 Apr 3;10(4):e1003531. doi: 10.1371/journal.pcbi.1003531 (PMC3974642; doi:10.1371/journal.pcbi.1003531)
Supplement: Protocol S1 — A zip file containing all supplementary source files. This includes the Rmd source code, HTML output, and all related documentation and code to completely and exactly recreate every results figure in this article. (ZIP) [file pcbi.1003531.s001.zip › norarefy-source/dispersion-survey/dispersion.html]

Dispersion Estimates from real data


# Dispersion Estimates from real data

As part of the demonstration that microbiome count data from amplicon sequencing experiments can be treated with the same procedures as for differential expression data from RNA-Seq experiments, we must demonstrate that microbiome data is overdispersed, that is, has an effectively non-zero value for dispersion parameter in the Negative Binomial generalization of Poisson discrete counts.

Now load packages, define themes.

```
# Load packages
library("phyloseq")
packageVersion("phyloseq")
```

```
## [1] '1.7.17'
```

```
library("DESeq")
packageVersion("DESeq")
```

```
## [1] '1.14.0'
```

```
library("ggplot2")
packageVersion("ggplot2")
```

```
## [1] '0.9.3.1'
```

```
library("plyr")
packageVersion("plyr")
```

```
## [1] '1.8'
```

```
# Set parameters
theme_set(theme_bw())
pal = "Dark2"
scale_colour_discrete <- function(palname = pal, ...) {
    scale_colour_brewer(palette = palname, ...)
}
scale_fill_discrete <- function(palname = pal, ...) {
    scale_fill_brewer(palette = palname, ...)
}
```

Now load data.

```
data(GlobalPatterns)
```

---

# Publicly Available Microbioime Count Data

The following describes publicly available microbiome count datasets available for direct download from the microbio.me/qiime web server. The phyloseq package includes a function, `microbio_me_qiime`, that downloads and imports data directly from this server by providing either the study ID, or file connection as argument. In some cases of larger data files, the studies were downloaded locally and imported from their local location, if present.

---

## Global Patterns - 3-prime

Study ID: `721`
Project Name: `CaporasoIlluminaPNAS2011_3prime`
Study Title: Global patterns of 16S rRNA diversity at a depth of millions of sequences per sample.
Study Abstract: The ongoing revolution in high-throughput sequencing continues to democratize the ability of small groups of investigators to map the microbial component of the biosphere. In particular, the coevolution of new sequencing platforms and new software tools allows data acquisition and analysis on an unprecedented scale. Here we report the next stage in this coevolutionary arms race, using the Illumina GAIIx platform to sequence a diverse array of 25 environmental samples and three known “mock communities” at a depth averaging 3.1 million reads per sample. We demonstrate excellent consistency in taxonomic recovery and recapture diversity patterns that were previously reported on the basis of metaanalysis of many studies from the literature (notably, the saline/nonsaline split in environmental samples and the split between host-associated and free-living communities). We also demonstrate that 2,000 Illumina single-end reads are sufficient to recapture the same relationships among samples that we observe with the full dataset. The results thus open up the possibility of conducting large-scale studies analyzing thousands of samples simultaneously to survey microbial communities at an unprecedented spatial and temporal resolution.
Pubmed ID (pmid): `20534432`

```
GPpath = "ftp://thebeast.colorado.edu/pub/QIIME_DB_Public_Studies/study_721_split_library_seqs_and_mapping.tgz"
GP = microbio_me_qiime(GPpath)
```

```
## Found biom-format file, now parsing it... 
## Done parsing biom... 
## Importing Sample Metdadata from mapping file...
## Merging the imported objects... 
## Successfully merged, phyloseq-class created. 
##  Returning...
```

```
GP
```

```
## phyloseq-class experiment-level object
## otu_table()   OTU Table:         [ 15809 taxa and 196 samples ]
## sample_data() Sample Data:       [ 196 samples by 59 sample variables ]
## tax_table()   Taxonomy Table:    [ 15809 taxa by 7 taxonomic ranks ]
```

```
gpbioreps = paste0(as(sample_data(GP)$ENV_BIOME, "character"), "--", as(sample_data(GP)$BODY_HABITAT, 
    "character"))
gpbioreps = gsub("ENVO:", "", gpbioreps, fixed = TRUE)
gpbioreps = gsub("UBERON:", "", gpbioreps, fixed = TRUE)
sample_data(GP)$BioReps <- gpbioreps
```

## Cigarette Smokers

Study ID: `524`
Project Name: `Charlson_cigarette_smokers`
Study Title: Disordered Microbial Communities in the Upper Respiratory Tract of Cigarette Smokers
Study Abstract: Cigarette smokers have an increased risk of infectious diseases involving the respiratory tract. Some effects of smoking on specific respiratory tract bacteria have been described, but the consequences for global airway microbial community composition have not been determined. Here, we used culture-independent high-density sequencing to analyze the microbiota from the right and left nasopharynx and oropharynx of 29 smoking and 33 nonsmoking healthy asymptomatic adults to assess microbial composition and effects of cigarette smoking. Bacterial communities were profiled using 454 pyrosequencing of 16S sequence tags (803,391 total reads), aligned to 16S rRNA databases, and communities compared using the UniFrac distance metric. A Random Forest machine-learning algorithm was used to predict smoking status and identify taxa that best distinguished between smokers and nonsmokers. Community composition was primarily determined by airway site, with individuals exhibiting minimal side-of-body or temporal variation. Within airway habitats, microbiota from smokers were significantly more diverse than nonsmokers and clustered separately. The distributions of several genera were systematically altered by smoking in both the oro- and nasopharynx, and there was an enrichment of anaerobic lineages associated with periodontal disease in the oropharynx. These results indicate that distinct regions of the human upper respiratory tract contain characteristic microbial communities that exhibit disordered patterns in cigarette smokers, both in individual components and global structure, which may contribute to the prevalence of respiratory tract complications in this population.
Pubmed ID (pmid): `21188149`

```
smokers = microbio_me_qiime(524)
```

```
## Found biom-format file, now parsing it... 
## Done parsing biom... 
## Importing Sample Metdadata from mapping file...
## Merging the imported objects... 
## Successfully merged, phyloseq-class created. 
##  Returning...
```

```
smokers
```

```
## phyloseq-class experiment-level object
## otu_table()   OTU Table:         [ 2156 taxa and 290 samples ]
## sample_data() Sample Data:       [ 290 samples by 53 sample variables ]
## tax_table()   Taxonomy Table:    [ 2156 taxa by 7 taxonomic ranks ]
```

```
sample_variables(smokers)
```

```
##  [1] "X.SampleID"                    "BarcodeSequence"              
##  [3] "LinkerPrimerSequence"          "TARGET_SUBFRAGMENT"           
##  [5] "AIRWAYSITE"                    "ASSIGNED_FROM_GEO"            
##  [7] "EXPERIMENT_CENTER"             "TITLE"                        
##  [9] "RUN_PREFIX"                    "AGE"                          
## [11] "SAMPLETYPE"                    "HOST_COMMON_NAME"             
## [13] "DEPTH"                         "HOST_TAXID"                   
## [15] "COMMON_NAME"                   "LONGITUDE"                    
## [17] "BODY_SITE"                     "ELEVATION"                    
## [19] "RUN_DATE"                      "COLLECTION_DATE"              
## [21] "ALTITUDE"                      "ENV_BIOME"                    
## [23] "SEX"                           "PLATFORM"                     
## [25] "COUNTRY"                       "ANTIBIOTICUSEPAST3MONTHS"     
## [27] "HOST_SUBJECT_ID"               "ANONYMIZED_NAME"              
## [29] "RESPIRATORYDISEASESTATUS"      "SAMPLE_CENTER"                
## [31] "SAMP_SIZE"                     "SIDEOFBODY"                   
## [33] "AGE_UNIT"                      "STUDY_ID"                     
## [35] "EXPERIMENT_DESIGN_DESCRIPTION" "Description_duplicate"        
## [37] "BODY_HABITAT"                  "SEQUENCING_METH"              
## [39] "TAXON_ID"                      "ENV_MATTER"                   
## [41] "TARGET_GENE"                   "ENV_FEATURE"                  
## [43] "KEY_SEQ"                       "BODY_PRODUCT"                 
## [45] "AGE_IN_YEARS"                  "RUN_CENTER"                   
## [47] "SMOKER"                        "PCR_PRIMERS"                  
## [49] "LIBRARY_CONSTRUCTION_PROTOCOL" "LATITUDE"                     
## [51] "REGION"                        "STUDY_CENTER"                 
## [53] "Description"
```

```
sample_data(smokers)$body_habitat <- gsub("UBERON:", "", sample_data(smokers)$BODY_HABITAT, 
    fixed = TRUE)
sample_data(smokers)$smoker <- ifelse(sample_data(smokers)$SMOKER == "y", "smoker", 
    "nonsmoker")
sample_data(smokers)$BioReps <- paste0(get_variable(smokers, "body_habitat"), 
    "-", get_variable(smokers, "smoker"))
# sample_data(smokers)$RESPIRATORYDISEASESTATUS
# sample_data(smokers)$HOST_SUBJECT_ID
```

## Long-Term Diet Patterns

Study ID: `1010`
Project Name: `Bushman_enterotypes_cafe`
Study Title: “Linking long-term dietary patterns with gut microbial enterotypes.”
Study Abstract: Diet strongly affects human health, partly by modulating gut microbiome composition. We used diet inventories and 16S rDNA sequencing to characterize fecal samples from 98 individuals. Fecal communities clustered into enterotypes distinguished primarily by levels of Bacteroides and Prevotella. Enterotypes were strongly associated with long-term diets, particularly protein and animal fat (Bacteroides) versus carbohydrates (Prevotella). A controlled-feeding study of 10 subjects showed that microbiome composition changed detectably within 24 hours of initiating a high-fat/low-fiber or low-fat/high-fiber diet, but that enterotype identity remained stable during the 10-day study. Thus, alternative enterotype states are associated with long-term diet.
Pubmed ID (pmid): `21885731`

```
# download/import
dipa = microbio_me_qiime(1010)
```

```
## Found biom-format file, now parsing it... 
## Done parsing biom... 
## Importing Sample Metdadata from mapping file...
## Merging the imported objects... 
## Successfully merged, phyloseq-class created. 
##  Returning...
```

```
dipa
```

```
## phyloseq-class experiment-level object
## otu_table()   OTU Table:         [ 1170 taxa and 95 samples ]
## sample_data() Sample Data:       [ 95 samples by 53 sample variables ]
## tax_table()   Taxonomy Table:    [ 1170 taxa by 7 taxonomic ranks ]
```

```
sample_sums(dipa)
```

```
## B.2008.10.S1.405544 B.2008.05.S1.405541 B.2011.01.S1.405525 
##                3379                3709                3796 
## B.2016.03.S1.405618 B.2009.07.S1.405524 B.2016.04.S1.405579 
##                3893                2985                4528 
## B.2016.06.S1.405581 B.2011.09.S1.405577 B.2004.07.S1.405529 
##                4693                3518                3178 
## B.2009.09.S1.405547 B.2009.02.S1.405570 B.2009.08.S1.405578 
##                2735                3322                2856 
## B.2009.10.S1.405585 B.2004.04.S1.405549 B.2016.05.S1.405583 
##                2980                3380                4419 
## B.2016.07.S1.405551 B.2016.02.S1.405600 B.2008.02.S1.405555 
##                4773                4408                4607 
## B.2011.10.S1.405602 B.2004.05.S1.405536 B.2004.08.S1.405610 
##                3384                3136                3069 
## B.2004.06.S1.405567 B.2016.08.S1.405590 B.2004.03.S1.405563 
##                3370                4732                3816 
## B.2009.03.S1.405564 B.2009.05.S1.405596 B.2009.04.S1.405531 
##                3316                3372                3118 
## B.2008.01.S1.405595 B.2009.06.S1.405554 B.2009.01.S1.405540 
##                4016                3391                3691 
## B.2004.02.S1.405533 B.2008.06.S1.405543 B.2008.08.S1.405550 
##                3121                3518                3464 
## B.2011.08.S1.405530 B.2004.01.S1.405609 B.2016.01.S1.405584 
##                3465                3182                4328 
## B.2008.07.S1.405599 B.2008.03.S1.405528 B.2011.03.S1.405538 
##                3290                3481                3574 
## B.2008.04.S1.405565 B.2011.05.S1.405539 B.2011.06.S1.405532 
##                4395                3683                2495 
## B.2011.07.S1.405534 B.2011.04.S1.405598 B.2008.09.S1.405553 
##                3264                3199                3072 
## B.2011.02.S1.405575 B.2006.02.S1.405606 B.2006.09.S1.405526 
##                3841                4125                4124 
## B.2006.01.S1.405568 B.2006.04.S1.405572 B.2006.03.S1.405589 
##                4967                4781                4419 
## B.2006.06.S1.405542 B.2006.07.S1.405582 B.2006.08.S1.405615 
##                3866                3591                3910 
## B.2006.05.S1.405597 B.2019.08.S1.405613 B.2012.04.S1.405562 
##                4062                3151                3002 
## B.2019.04.S1.405614 B.2012.02.S1.405552 B.2005.09.S1.405592 
##                3875                3436                3272 
## B.2005.02.S1.405604 B.2005.05.S1.405611 B.2005.04.S1.405545 
##                3034                3611                3308 
## B.2005.10.S1.405573 B.2019.09.S1.405605 B.2005.03.S1.405591 
##                3030                2953                2593 
## B.2019.07.S1.405558 B.2019.02.S1.405607 B.2005.06.S1.405537 
##                3394                4016                3617 
## B.2005.07.S1.405586 B.2019.01.S1.405535 B.2019.03.S1.405560 
##                3235                4056                3506 
## B.2012.08.S1.405556 B.2019.05.S1.405612 B.2005.08.S1.405559 
##                3540                3451                2888 
## B.2012.06.S1.405601 B.2005.01.S1.405588 B.2012.09.S1.405527 
##                3115                3166                3535 
## B.2012.03.S1.405546 B.2012.07.S1.405580 B.2012.10.S1.405569 
##                3760                3027                3376 
## B.2019.10.S1.405561 B.2019.06.S1.405608 B.2012.01.S1.405593 
##                2966                2840                3240 
## B.2012.05.S1.405616 B.2020.05.S1.405574 B.2020.04.S1.405587 
##                3597                3894                3626 
## B.2020.06.S1.405557 B.2020.01.S1.405576 B.2020.08.S1.405603 
##                3612                3994                3676 
## B.2020.10.S1.405617 B.2020.09.S1.405594 B.2020.02.S1.405548 
##                3148                3640                4348 
## B.2020.03.S1.405571 B.2020.07.S1.405566 
##                3826                3221
```

```
sample_data(dipa)$BioReps <- paste0(get_variable(dipa, "HOST_SUBJECT_ID"), "--", 
    get_variable(dipa, "GROUP"))
sample_data(dipa)$BioReps
```

```
##  [1] "2008--LowFat"  "2008--LowFat"  "2011--Before"  "2016--HighFat"
##  [5] "2009--HighFat" "2016--HighFat" "2016--HighFat" "2011--LowFat" 
##  [9] "2004--HighFat" "2009--HighFat" "2009--HighFat" "2009--HighFat"
## [13] "2009--HighFat" "2004--HighFat" "2016--HighFat" "2016--HighFat"
## [17] "2016--HighFat" "2008--LowFat"  "2011--LowFat"  "2004--HighFat"
## [21] "2004--HighFat" "2004--HighFat" "2016--HighFat" "2004--HighFat"
## [25] "2009--HighFat" "2009--HighFat" "2009--HighFat" "2008--Before" 
## [29] "2009--HighFat" "2009--Before"  "2004--HighFat" "2008--LowFat" 
## [33] "2008--LowFat"  "2011--LowFat"  "2004--Before"  "2016--Before" 
## [37] "2008--LowFat"  "2008--LowFat"  "2011--LowFat"  "2008--LowFat" 
## [41] "2011--LowFat"  "2011--LowFat"  "2011--LowFat"  "2011--LowFat" 
## [45] "2008--LowFat"  "2011--LowFat"  "2006--HighFat" "2006--HighFat"
## [49] "2006--Before"  "2006--HighFat" "2006--HighFat" "2006--HighFat"
## [53] "2006--HighFat" "2006--HighFat" "2006--HighFat" "2019--HighFat"
## [57] "2012--LowFat"  "2019--HighFat" "2012--LowFat"  "2005--LowFat" 
## [61] "2005--LowFat"  "2005--LowFat"  "2005--LowFat"  "2005--LowFat" 
## [65] "2019--HighFat" "2005--LowFat"  "2019--HighFat" "2019--HighFat"
## [69] "2005--LowFat"  "2005--LowFat"  "2019--Before"  "2019--HighFat"
## [73] "2012--LowFat"  "2019--HighFat" "2005--LowFat"  "2012--LowFat" 
## [77] "2005--Before"  "2012--LowFat"  "2012--LowFat"  "2012--LowFat" 
## [81] "2012--LowFat"  "2019--HighFat" "2019--HighFat" "2012--Before" 
## [85] "2012--LowFat"  "2020--LowFat"  "2020--LowFat"  "2020--LowFat" 
## [89] "2020--Before"  "2020--LowFat"  "2020--LowFat"  "2020--LowFat" 
## [93] "2020--LowFat"  "2020--LowFat"  "2020--LowFat"
```

## Quality filtering

Study ID: `1689`
Project Name: `bokulich_quality_dataset9`
(Actually appears to have multiple entries with 2, 3, 6, 9, 10 at the end)
Study Title: Quality-filtering vastly improves diversity estimates from Illumina amplicon sequencing
Study Abstract: High-throughput sequencing has revolutionized microbial ecology, but read quality remains a considerable barrier to accurate taxonomy assignment and alpha-diversity assessment for microbial communities. We demonstrate that high-quality read length and abundance are the primary factors differentiating correct from erroneous reads produced by Illumina GAIIx, HiSeq and MiSeq instruments. We present guidelines for user-defined quality-filtering strategies, enabling efficient extraction of high-quality data and facilitating interpretation of Illumina sequencing results.
Pubmed ID (pmid): `23202435`

A few of these datasets I have downloaded, in case I want to try.

## Human Microbiome Project

Human Microbiome Project (2007)

The NIH Human Microbiome Project (2009)

human microbiome community profiling (2009)

Study ID: `969`
Project Name: `HMP_v35`
Variable Region v3-v5 data.
Study ID: `968`
Project Name: `HMP_v13`
Variable Region v1-v3 data.

Study Title: `HMP_production_phase_1`
Study Abstract: This HMP production phase represents pyrosequencing of 16S rRNA genes amplified from multiple body sites across hundreds of human subjects. There are two time points represented for a subset of these subjects. Using default protocol v4.2., data for the 16S window spanning V3-V5 was generated for all samples, with a second 16S window spanning V1-V3 generated for a majority of the samples. 16S rRNA sequencing is being used to characterize the complexity of microbial communities at individual body sites, and to determine whether there is a core microbiome at each site. Several body sites will be studied, including the gastrointestinal and female urogenital tracts, oral cavity, nasal and pharyngeal tract, and skin.
Pubmed ID (pmid): (multiple articles based on this and/or corresponding metagenome data)

```
# hmpv35 =
# microbio_me_qiime('~/Downloads/study_969_split_library_seqs_and_mapping.tgz')
# hmpv35 hmpv13 =
# microbio_me_qiime('~/Downloads/study_968_split_library_seqs_and_mapping.tgz')
# hist(sample_sums(hmpv35), xlim=c(0, 4e4), breaks=50)
# hist(sample_sums(hmpv13), xlim=c(0, 4e4), breaks=50)
```

## Turnbaugh Humanized Mice

The effect of diet on the human gut microbiome: a metagenomic analysis in humanized gnotobiotic mice

Study ID: `453`, and others
Project Name: `Turnbaugh_hmice_biogeography`
Study Title: “The effect of diet on the human gut microbiome: a metagenomic analysis in humanized gnotobiotic mice”
Study Abstract: Diet and nutritional status are among the most important modifiable determinants of human health. The nutritional value of food is influenced in part by a persons gut microbial community (microbiota) and its component genes (microbiome). Unraveling the interrelations among diet, the structure and operations of the gut microbiota, and nutrient and energy harvest is confounded by variations in human environmental exposures, microbial ecology, and genotype. To help overcome these problems, we created a well-defined, representative animal model of the human gut ecosystem by transplanting fresh or frozen adult human fecal microbial communities into germ-free C57BL/6J mice. Culture-independent metagenomic analysis of the temporal, spatial, and intergenerational patterns of bacterial colonization showed that these humanized mice were stably and heritably colonized and reproduced much of the bacterial diversity of the donors microbiota. Switching from a low-fat, plant polysaccharide-rich diet to a high-fat, high-sugar Western diet shifted the structure of the microbiota within a single day, changed the representation of metabolic pathways in the microbiome, and altered microbiome gene expression. Reciprocal transplants involving various combinations of donor and recipient diets revealed that colonization history influences the initial structure of the microbial community but that these effects can be rapidly altered by diet. Humanized mice fed the Western diet have increased adiposity; this trait is transmissible via microbiota transplantation. Humanized gnotobiotic mice will be useful for conducting proof-of-principle clinical trials that test the effects of environmental and genetic factors on the gut microbiota and host physiology. Nearly full-length 16S rRNA gene sequences are deposited in GenBank under the accession numbers GQ491120 to GQ493997.
Pubmed ID (pmid): `20368178`

## Soil Bacteria and Archaea

Study ID: `928`
Project Name: `Bates_global_dist_archaea_soils`
Study Title: Examining the global dominant archaeal populations in soil
Study Abstract: Archaea, primarily Crenarchaeota, are common in soil; however, the structure of soil archaeal communities and the factors regulating their diversity and abundance remain poorly understood. Here, we used barcoded pyrosequencing to comprehensively survey archaeal and bacterial communities in 146 soils, representing a multitude of soil and ecosystem types from across the globe. Relative archaeal abundance, the percentage of all 16S rRNA gene sequences recovered that were archaeal, averaged 2 percent across all soils and ranged from 0 percent to greater than 10 percent in individual soils. Soil C:N ratio was the only factor consistently correlated with archaeal relative abundances, being higher in soils with lower C:N ratios. Soil archaea communities were dominated by just two phylotypes from a constrained clade within the Crenarchaeota, which together accounted for greater than 70 percent of all archaeal sequences obtained in the survey. As one of these phylotypes was closely related to a previously identified putative ammonia oxidizer, we sampled from two long-term nitrogen (N) addition experiments to determine if this taxon responds to experimental manipulations of N availability. Contrary to expectations, the abundance of this dominant taxon, as well as archaea overall, tended to decline with increasing N. This trend was coupled with a concurrent increase in known N-oxidizing bacteria, suggesting competitive interactions between these groups.
Pubmed ID (pmid): `21085198`

```
archsoil = microbio_me_qiime(928)
```

```
## Found biom-format file, now parsing it... 
## Done parsing biom... 
## Importing Sample Metdadata from mapping file...
## Merging the imported objects... 
## Successfully merged, phyloseq-class created. 
##  Returning...
```

```
archsoil
```

```
## phyloseq-class experiment-level object
## otu_table()   OTU Table:         [ 4631 taxa and 145 samples ]
## sample_data() Sample Data:       [ 145 samples by 46 sample variables ]
## tax_table()   Taxonomy Table:    [ 4631 taxa by 7 taxonomic ranks ]
```

```
sample_variables(archsoil)
```

```
##  [1] "X.SampleID"                    "BarcodeSequence"              
##  [3] "LinkerPrimerSequence"          "TARGET_SUBFRAGMENT"           
##  [5] "ASSIGNED_FROM_GEO"             "EXPERIMENT_CENTER"            
##  [7] "TITLE"                         "RUN_PREFIX"                   
##  [9] "TEXTURE"                       "TAXON_ID"                     
## [11] "DEPTH"                         "TOT_ORG_CARB"                 
## [13] "COMMON_NAME"                   "ELEVATION"                    
## [15] "RUN_DATE"                      "TOT_NITRO"                    
## [17] "COLLECTION_DATE"               "ALTITUDE"                     
## [19] "ENV_BIOME"                     "PLATFORM"                     
## [21] "CARB_NITRO_RATIO"              "ANNUAL_SEASON_TEMP"           
## [23] "COUNTRY"                       "PH"                           
## [25] "ANONYMIZED_NAME"               "SAMPLE_CENTER"                
## [27] "SAMP_SIZE"                     "LONGITUDE"                    
## [29] "STUDY_ID"                      "EXPERIMENT_DESIGN_DESCRIPTION"
## [31] "Description_duplicate"         "SEQUENCING_METH"              
## [33] "ENV_MATTER"                    "TARGET_GENE"                  
## [35] "ENV_FEATURE"                   "KEY_SEQ"                      
## [37] "AGE_IN_YEARS"                  "RUN_CENTER"                   
## [39] "PCR_PRIMERS"                   "LIBRARY_CONSTRUCTION_PROTOCOL"
## [41] "ANNUAL_SEASON_PRECPT"          "LATITUDE"                     
## [43] "SOIL_MOISTURE_DEFICIT"         "REGION"                       
## [45] "STUDY_CENTER"                  "Description"
```

```
sample_data(archsoil)$LONGITUDE
```

```
##   [1]  -85.40  -85.40  -85.40  -85.40  -85.40  -85.40  -85.40  -85.40
##   [9]  -85.40  -85.40  -85.40  -85.40  -85.40  -85.40  -85.40  -85.40
##  [17]  -93.20  -93.20  -93.20  -93.20  -93.20  -93.20  -54.68  -54.68
##  [25]  -54.68  -80.05 -102.38 -148.25 -111.77 -111.77  -74.26  -74.10
##  [33]  -81.67  -81.67  -96.23 -105.33 -104.83  -97.23  -81.67  -81.67
##  [41]  -81.67  -81.67  -81.67  -81.67  -81.67  -81.67  -81.67  -81.67
##  [49]  -93.20  -93.20  -93.20  -93.20  -93.20  -93.20  -79.08  -79.08
##  [57]  163.88  162.31  162.25  162.89  162.89  162.88  161.70  161.31
##  [65]  -93.20  -93.20  -93.20  -93.20  -93.20  -93.20 -111.45 -111.48
##  [73] -155.70 -155.70 -122.15  -73.75  -73.75  -95.17  -96.60  -96.60
##  [81]  -65.83  -65.83 -115.60 -115.65 -115.87 -115.87 -105.48 -105.48
##  [89] -105.48 -105.48 -105.48 -105.48 -105.48 -105.48  -96.88  -96.86
##  [97]  -96.81  -95.28  -97.11  -97.27  -94.56  -95.58  -96.23  -92.38
## [105]  -93.65  -96.97  -92.85  -96.78  -96.15  -96.71  -95.70  -96.57
## [113]  -96.60  -96.32  -97.36  -96.42  -96.43  -96.82  -96.49  -95.27
## [121]  -95.27  -95.20  -95.54  -96.18  -96.53  -71.27  -71.23  -71.23
## [129]  -96.87 -111.55 -105.93 -105.93 -104.70 -105.20 -106.20 -118.17
## [137] -118.70 -118.63 -120.05 -120.03 -120.05 -106.73 -106.73 -149.58
## [145] -149.58
```

```
sample_data(archsoil)$BioReps <- factor(paste0(round(sample_data(archsoil)$LONGITUDE, 
    1), "--", round(sample_data(archsoil)$LATITUDE), 1))
```

Probably not enough biological replicates to include this one for variance estimates.

## Restroom Biogeography

Study ID: `1335`
Project Name: `Flores_restroom_surface_biogeography`
Study Title: Microbial biogeography of public restroom surfaces
Study Abstract: We spend the majority of our lives indoors where we are constantly exposed to bacteria residing on surfaces. However, the diversity of these surface-associated communities is largely unknown. We explored the biogeographical patterns exhibited by bacteria across ten surfaces within each of twelve public restrooms. Using high-throughput barcoded pyrosequencing of the 16S rRNA gene, we identified 19 bacterial phyla across all surfaces. Most sequences belonged to four phyla: Actinobacteria, Bacteriodetes, Firmicutes and Proteobacteria. The communities clustered into three general categories: those found on surfaces associated with toilets, those on the restroom floor, and those found on surfaces routinely touched with hands. On toilet surfaces, gut-associated taxa were more prevalent, suggesting fecal contamination of these surfaces. Floor surfaces were the most diverse of all communities and contained several taxa commonly found in soils. Skin-associated bacteria, especially the Propionibacteriaceae, dominated surfaces routinely touched with our hands. Certain taxa were more common in female than in male restrooms as vagina-associated Lactobacillaceae were widely distributed in female restrooms, likely from urine contamination. Use of the SourceTracker algorithm confirmed many of our taxonomic observations as human skin was the primary source of bacteria on restroom surfaces. Overall, these results demonstrate that restroom surfaces host relatively diverse microbial communities dominated by human-associated bacteria with clear linkages between communities on or in different body sites and those communities found on restroom surfaces. More generally, this work is relevant to the public health field as we show that human associated microbes are commonly found on restroom surfaces suggesting that bacterial pathogens could readily be transmitted between individuals by the touching of surfaces. Furthermore, we demonstrate that we can use high-throughput analyses of bacterial communities to determine sources of bacteria on indoor surfaces, an approach which could be used to track pathogen transmission and test the efficacy of hygiene practices.
Pubmed ID (pmid): `22132229`

```
restrooms = microbio_me_qiime(1335)
```

```
## Found biom-format file, now parsing it... 
## Done parsing biom... 
## Importing Sample Metdadata from mapping file...
```

```
## Warning: EOF within quoted string
## Warning: number of items read is not a multiple of the number of columns
```

```
## Merging the imported objects... 
## Successfully merged, phyloseq-class created. 
##  Returning...
```

```
restrooms
```

```
## phyloseq-class experiment-level object
## otu_table()   OTU Table:         [ 4467 taxa and 52 samples ]
## sample_data() Sample Data:       [ 52 samples by 42 sample variables ]
## tax_table()   Taxonomy Table:    [ 4467 taxa by 7 taxonomic ranks ]
```

```
sample_variables(restrooms)
```

```
##  [1] "X.SampleID"                    "BarcodeSequence"              
##  [3] "LinkerPrimerSequence"          "TARGET_SUBFRAGMENT"           
##  [5] "ASSIGNED_FROM_GEO"             "EXPERIMENT_CENTER"            
##  [7] "TITLE"                         "RUN_PREFIX"                   
##  [9] "TAXON_ID"                      "DEPTH"                        
## [11] "COMMON_NAME"                   "ELEVATION"                    
## [13] "RUN_DATE"                      "COLLECTION_DATE"              
## [15] "GENDER"                        "ALTITUDE"                     
## [17] "LEVEL"                         "ENV_BIOME"                    
## [19] "PLATFORM"                      "COUNTRY"                      
## [21] "SAMPLE_CENTER"                 "SAMP_SIZE"                    
## [23] "FLOOR"                         "LONGITUDE"                    
## [25] "STUDY_ID"                      "EXPERIMENT_DESIGN_DESCRIPTION"
## [27] "Description_duplicate"         "SEQUENCING_METH"              
## [29] "ENV_MATTER"                    "TARGET_GENE"                  
## [31] "BUILDING"                      "ENV_FEATURE"                  
## [33] "KEY_SEQ"                       "AGE_IN_YEARS"                 
## [35] "RUN_CENTER"                    "SURFACE"                      
## [37] "PCR_PRIMERS"                   "LIBRARY_CONSTRUCTION_PROTOCOL"
## [39] "LATITUDE"                      "REGION"                       
## [41] "STUDY_CENTER"                  "Description"
```

```
sample_data(restrooms)$BioReps <- factor(paste0(sample_data(restrooms)$SURFACE, 
    "--", sample_data(restrooms)$GENDER))
```

---

Zinger et al. 2011 PLoS ONE
Global patterns of bacterial beta-diversity in seafloor and seawater ecosystems

---

# Function for estimating common-scale mean, variance

We will use this to calculate the common-scale mean/variance for each OTU of each dataset without any data subsampling.

```
deseq_var = function(physeq, bioreps) {
    require("DESeq")
    require("plyr")
    # physeq = GP bioreps = 'BioReps'
    biorep_levels = levels(factor(get_variable(physeq, bioreps)))
    ldfs = lapply(biorep_levels, function(L, physeq, bioreps) {
        # L = biorep_levels[1]
        whsa = as(get_variable(physeq, bioreps), "character") %in% L
        # Skip if there are 2 or fewer instances among BioReps Prob don't want to
        # assume very much from estimates of variance for which N<=2
        if (sum(whsa) <= 2) {
            return(NULL)
        }
        ps1 = prune_samples(whsa, physeq)
        # Prune the 'zero' OTUs from this sample set.
        ps1 = prune_taxa(taxa_sums(ps1) > 5, physeq)
        # Coerce to matrix, round up to nearest integer just in case
        x = ceiling(as(otu_table(ps1), "matrix"))
        # Add 1 to everything to avoid log-0 problems
        x = x + 1
        cds = newCountDataSet(countData = x, conditions = get_variable(physeq, 
            bioreps))
        # Estimate Size factors
        cds = estimateSizeFactors(cds, locfunc = median)
        # Estimate Dispersions, DESeq
        cds = estimateDispersions(cds, method = "pooled", sharingMode = "fit-only", 
            fitType = "local")
        # get the scale-normalized mean and variance
        df = getBaseMeansAndVariances(counts(cds), sizeFactors(cds))
        colnames(df) <- c("Mean", "Variance")
        ## Calculate Mean and Variance for each OTU after rarefying Use a non-random
        ## definition of 'rarefying'
        ps1r = transform_sample_counts(ps1, function(x, Smin = min(sample_sums(ps1), 
            na.rm = TRUE)) {
            round(x * Smin/sum(x), digits = 0)
        })
        ps1r = prune_taxa(taxa_sums(ps1r) > 5, ps1r)
        rdf = data.frame(Mean = apply(otu_table(ps1r), 1, mean), Variance = apply(otu_table(ps1r), 
            1, var))
        # Return results, including cds and 'rarefied' mean-var
        return(list(df = df, cds = cds, rdf = rdf))
    }, physeq, bioreps)
    names(ldfs) <- biorep_levels
    # Remove the elements that were skipped.
    ldfs <- ldfs[!sapply(ldfs, is.null)]
    # Group into data.frames of variance estimates
    df = ldply(ldfs, function(L) {
        data.frame(L$df, OTUID = rownames(L$df))
    })
    rdf = ldply(ldfs, function(L) {
        data.frame(L$rdf, OTUID = rownames(L$rdf))
    })
    colnames(df)[1] <- bioreps
    colnames(rdf)[1] <- bioreps
    # Remove values that are zero for both
    df <- df[!(df$Mean == 0 & df$Variance == 0), ]
    rdf <- rdf[!(rdf$Mean == 0 & rdf$Variance == 0), ]
    # Create a `data.frame` of the DESeq predicted.
    deseqfitdf = ldply(ldfs, function(x) {
        df = x$df
        xg = seq(range(df$Mean)[1], range(df$Mean)[2], length.out = 100)
        fitVar = xg + fitInfo(x$cds)$dispFun(xg) * xg^2
        return(data.frame(Mean = xg, Variance = fitVar))
    })
    colnames(deseqfitdf)[1] <- bioreps
    return(list(datadf = df, fitdf = deseqfitdf, raredf = rdf))
}
```

Define a function for plotting this data.frame

```
plot_dispersion <- function(datadf, fitdf, title = "Microbiome Counts, Common-Scale", 
    xbreaks = c(10, 1000, 1e+05)) {
    require("ggplot2")
    p = ggplot(datadf, aes(Mean, Variance)) + geom_point(alpha = 0.25, size = 1.5) + 
        facet_wrap(~BioReps)
    p = p + scale_y_log10()
    p = p + scale_x_log10(breaks = xbreaks, labels = xbreaks)
    p = p + geom_abline(intercept = 0, slope = 1, linetype = 1, size = 0.5, 
        color = "gray60")
    p = p + geom_line(data = fitdf, color = "red", linetype = 1, size = 0.5)
    p = p + ggtitle(title)
    return(p)
}
```

---

# Create Figure 1 for article.

```
# Restrooms
restrovar = deseq_var(restrooms, "BioReps")
plot_dispersion(restrovar$datadf, restrovar$fitdf)
```

```
# Smoker
smokervar = deseq_var(smokers, "BioReps")
plot_dispersion(smokervar$datadf, smokervar$fitdf)
```

```
# GP
GPvar = deseq_var(GP, "BioReps")
plot_dispersion(GPvar$datadf, GPvar$fitdf)
```

```
# Diet Patterns (Bushman)
dipavar = deseq_var(dipa, "BioReps")
plot_dispersion(dipavar$datadf, dipavar$fitdf)
```

Plot Variance-Mean of rarefied counts among biological replicates. (Note: this uses the expected-value subsampling transformation, rather than random subsampling).

```
GPvar$raredf$BioReps <- substr(GPvar$raredf$BioReps, 1, 30)
ggplot(GPvar$raredf, aes(Mean, Variance)) + geom_point() + scale_y_log10() + 
    scale_x_log10() + facet_wrap(~BioReps) + geom_abline(intercept = 0, slope = 1, 
    linetype = 1, size = 0.5, color = "gray60")
```

```
# Smokers
ggplot(smokervar$raredf, aes(Mean, Variance)) + geom_point() + scale_y_log10() + 
    scale_x_log10() + facet_wrap(~BioReps) + geom_abline(intercept = 0, slope = 1, 
    linetype = 1, size = 0.5, color = "gray60")
```

```
# dipa
ggplot(dipavar$raredf, aes(Mean, Variance)) + geom_point() + scale_y_log10() + 
    scale_x_log10() + facet_wrap(~BioReps) + geom_abline(intercept = 0, slope = 1, 
    linetype = 1, size = 0.5, color = "gray60")
```

```
# Restroom Surfaces
ggplot(restrovar$raredf, aes(Mean, Variance)) + geom_point() + scale_y_log10() + 
    scale_x_log10() + facet_wrap(~BioReps) + geom_abline(intercept = 0, slope = 1, 
    linetype = 1, size = 0.5, color = "gray60")
```

---

## Combined figure.

```
varlist = list(DietPatterns = dipavar, Smokers = smokervar, GlobalPatterns = GPvar, 
    RestroomSurfaces = restrovar)
# Organize the three data.frames for plotting
comvardf = ldply(varlist, function(x) {
    x$datadf
})
fitvardf = ldply(varlist, function(x) {
    x$fitdf
})
rarvardf = ldply(varlist, function(x) {
    x$raredf
})
# Shorten the panel names (Biological Replicates)
rarvardf$BioReps <- substr(rarvardf$BioReps, 1, 30)
fitvardf$BioReps <- substr(fitvardf$BioReps, 1, 30)
comvardf$BioReps <- substr(comvardf$BioReps, 1, 30)
# Remove the pointlessly small subsets of data within a bio replicate
rarvardf = ddply(rarvardf, "BioReps", function(df) {
    if (nrow(df) > 10) {
        return(df)
    }
})
fitvardf = ddply(fitvardf, "BioReps", function(df) {
    if (nrow(df) > 10) {
        return(df)
    }
})
comvardf = ddply(comvardf, "BioReps", function(df) {
    if (nrow(df) > 10) {
        return(df)
    }
})
# Label the study name variable
colnames(comvardf)[1] = colnames(fitvardf)[1] = (colnames(rarvardf)[1] <- "Study")
# Make combined plot from rarefied counts
prare <- ggplot(rarvardf, aes(Mean, Variance)) + geom_point() + scale_y_log10() + 
    scale_x_log10() + facet_wrap(~Study + BioReps, scales = "free") + geom_abline(intercept = 0, 
    slope = 1, linetype = 1, size = 0.5, color = "gray60") + ggtitle("Variance versus Mean on Rarefied Counts")
prare
```

```
# Make combined plot of deseq counts
p = ggplot(comvardf, aes(Mean, Variance)) + geom_point(alpha = 0.25, size = 1.5) + 
    facet_wrap(~Study + BioReps, scales = "free")
p = p + scale_y_log10()
p = p + scale_x_log10()
p = p + geom_abline(intercept = 0, slope = 1, linetype = 1, size = 0.5, color = "gray60")
p = p + geom_line(data = fitvardf, color = "red", linetype = 1, size = 0.5)
p = p + ggtitle("Common-Scale Variance versus Mean, multiple studies/replicates")
p
```

```
bigdf = ldply(list(rarefied = rarvardf, common_scale = comvardf))
colnames(bigdf)[1] <- "Type"
# Make combined plot
pbig = ggplot(bigdf, aes(Mean, Variance)) + geom_point(alpha = 0.25, size = 1.5) + 
    facet_grid(Study + BioReps ~ Type)  #, scales='free')
pbig = pbig + scale_y_log10()
pbig = pbig + scale_x_log10()
pbig = pbig + geom_abline(intercept = 0, slope = 1, linetype = 1, size = 0.5, 
    color = "gray60")
pbig = pbig + geom_line(data = fitvardf, color = "red", linetype = 1, size = 0.5)
pbig = pbig + ggtitle("Common-Scale Variance versus Mean, multiple studies/replicates")
pbig = pbig + theme(text = element_text(size = 5))
```

## Figure 1 rev 2

```
# First, subset
fig1st = c("GlobalPatterns", "DietPatterns")
# fig1br = c('2004--HighFat', '2008--LowFat', 'marine biome--None',
# 'freshwater biome--None', 'human-associated habitat--oral')
fig1br = c("2004--HighFat", "marine biome--None", "freshwater biome--None")
subsetdf = subset(bigdf, BioReps %in% fig1br & Study %in% fig1st)
# Define subfitvardf, the data.frame to show the DESeq 'fit'.
subfitvardf = fitvardf
subfitvardf$Type <- "common_scale"
subfitvardf = subset(subfitvardf, BioReps %in% fig1br & Study %in% fig1st)
# Trim points for which mean is small.
subsetdf = subsetdf[subsetdf$Mean >= 2, ]
# Format the Type labels for plotting.
subsetdf$Type <- gsub("common_scale", "Common Scale", subsetdf$Type, fixed = TRUE)
subfitvardf$Type <- gsub("common_scale", "Common Scale", subfitvardf$Type, fixed = TRUE)
subsetdf$Type <- gsub("rarefied", "Rarefied", subsetdf$Type, fixed = TRUE)
# Replace bioreplicate names, as needed.
subsetdf$BioReps <- gsub(" biome--None", "", subsetdf$BioReps, fixed = TRUE)
subfitvardf$BioReps <- gsub(" biome--None", "", subfitvardf$BioReps, fixed = TRUE)
subsetdf$BioReps <- gsub("--", " ", subsetdf$BioReps, fixed = TRUE)
subfitvardf$BioReps <- gsub("--", " ", subfitvardf$BioReps, fixed = TRUE)
```

Now plot commands

```
# pF1r2 = ggplot(subsetdf, aes(Mean, Variance, color=Type))
pF1r2 = ggplot(subsetdf, aes(Mean, Variance))
pF1r2 = pF1r2 + geom_point(alpha = 0.5, size = 1)
pF1r2 = pF1r2 + facet_grid(Study + BioReps ~ Type)
# pF1r2 = pF1r2 + facet_wrap(~ Study + BioReps, ncol=1)
pF1r2 = pF1r2 + scale_y_log10()
pF1r2 = pF1r2 + scale_x_log10()
pF1r2 = pF1r2 + geom_abline(intercept = 0, slope = 1, linetype = 5, size = 0.5, 
    color = "gray60")
pF1r2 = pF1r2 + geom_line(data = subfitvardf, color = "cyan", linetype = 1, 
    size = 0.5)
# pF1r2 = pF1r2 + ggtitle('Common-Scale Variance versus Mean\n multiple
# studies/replicates')
require("grid")  # For unit()
```

```
## Loading required package: grid
```

```
pF1r2 = pF1r2 + theme(text = element_text(size = 8), legend.position = "top", 
    plot.margin = unit(c(0, 0, 0, 0), "cm"))
pF1r2
```

```
dev.off()
```

```
## null device 
##           1
```

```
# One-column figure width according to PLoS Comp Biol instructions:
# width=3.27
# http://www.ploscompbiol.org/static/figureGuidelines.action#dimensions
ggsave("microbiome-dispersion-survey.pdf", plot = pF1r2, width = 3.27)
```

```
## Saving 3.27 x 7 in image
```

Additional plot showing the relative change in variance before and after rarefying.

```
# Reshape to plot common-scale versus rarefied.
require("reshape2")
```

```
## Loading required package: reshape2
```

```
submeandf = dcast(subsetdf, BioReps + Study + OTUID ~ Type, value.var = "Mean")
subvardf = dcast(subsetdf, BioReps + Study + OTUID ~ Type, value.var = "Variance")
bothdf = ldply(list(Mean = submeandf, Variance = subvardf))
colnames(bothdf)[1] <- "MV"
bothdf$StudyBiorep <- paste(bothdf$Study, bothdf$BioReps, sep = ", ")
# rm NAs
bothdf = bothdf[!is.na(bothdf$Rarefied), ]
# Plot them together
pVvV = ggplot(bothdf, aes(`Common Scale`, Rarefied, color = StudyBiorep))
pVvV = pVvV + geom_point(alpha = 0.65, size = 2)
pVvV = pVvV + scale_y_log10()
pVvV = pVvV + scale_x_log10()
pVvV = pVvV + ylab("Rarefied") + xlab("Common Scale")
pVvV = pVvV + facet_wrap(~MV)
require("grid")  # For unit()
pVvV = pVvV + theme(text = element_text(size = 8), plot.margin = unit(c(0, 0, 
    -0.12, 0), "cm"), legend.key.size = unit(0.54, "lines"), legend.position = c(0.24, 
    0.75), legend.text = element_text(size = 5))
pVvV
```

## Conclusion

As you can see, overdispersion is an important feature of this count data before and after rarefying. We retain much more information about the dependency of overdispersion on the mean if we do not rarefy.

## Save image of R workspace after run.

```
save.image("microbiome-dispersion-survey.RData")
```
