# Supplementary material for: Waste Not, Want Not: Why Rarefying Microbiome Data Is Inadmissible
Source: PLoS Comput Biol. 2014 Apr 3;10(4):e1003531. doi: 10.1371/journal.pcbi.1003531 (PMC3974642; doi:10.1371/journal.pcbi.1003531)
Supplement: Protocol S1 — A zip file containing all supplementary source files. This includes the Rmd source code, HTML output, and all related documentation and code to completely and exactly recreate every results figure in this article. (ZIP) [file pcbi.1003531.s001.zip › norarefy-source/minimal-rarefy-example/minimal-rarefy-example.html]

A minimal example of rarefying


# A minimal example of rarefying

In order to provide the statistical framework for the variance stabilizing transformations performed in this paper, we provide a simple example. When comparing two different samples called `A` and `B` for which we have 100 and 1000 reads respectively. Suppose there are only two types of taxa present, `OTU1` and `OTU2`, according to the following contingency table:

```
tab2 <- structure(c(62, 38, 500, 500), .Dim = c(2L, 2L), .Dimnames = list(c("OTU1", 
    "OTU2"), c("A", "B")))
tab2
```

```
##       A   B
## OTU1 62 500
## OTU2 38 500
```

Suppose now that we performed a rarefying procedure, which transforms the counts in sample `B` so that their sum is now the same as sample `A`. Further assume that our rarefying procedure perfectly reflects the original proportions. In practice it won't (see section on **Rarefying introduces additional sampling noise** below).

```
tab2r <- matrix(c(62, 38, 50, 50), 2, 2, dimnames = list(c("OTU1", "OTU2"), 
    c("A", "B")))
tab2r
```

```
##       A  B
## OTU1 62 50
## OTU2 38 50
```

Testing the two proportions according to a standard test is done either using a \( \chi^2 \) test or a Fisher exact test. Here are the results of both tests, for both the original and “rarefied” data.

```
chisq.test(tab2)
```

```
## 
##  Pearson's Chi-squared test with Yates' continuity correction
## 
## data:  tab2
## X-squared = 4.77, df = 1, p-value = 0.02897
```

```
fisher.test(tab2)
```

```
## 
##  Fisher's Exact Test for Count Data
## 
## data:  tab2
## p-value = 0.0272
## alternative hypothesis: true odds ratio is not equal to 1
## 95 percent confidence interval:
##  1.050 2.561
## sample estimates:
## odds ratio 
##      1.631
```

```
chisq.test(tab2r)
```

```
## 
##  Pearson's Chi-squared test with Yates' continuity correction
## 
## data:  tab2r
## X-squared = 2.455, df = 1, p-value = 0.1171
```

```
fisher.test(tab2r)
```

```
## 
##  Fisher's Exact Test for Count Data
## 
## data:  tab2r
## p-value = 0.1169
## alternative hypothesis: true odds ratio is not equal to 1
## 95 percent confidence interval:
##  0.8949 2.9792
## sample estimates:
## odds ratio 
##      1.628
```

Here is how to store the results of each test in a matrix.

```
chi0 = chisq.test(tab2)
fis0 = fisher.test(tab2)
chir = chisq.test(tab2r)
fisr = fisher.test(tab2r)
pvals = c(chi0$p.value, chir$p.value, fis0$p.value, fisr$p.value)
Ptab = matrix(round(pvals, 4), 2, 2, dimnames = list(c("original", "rarefied"), 
    c("Chi-Squared", "Fisher")))
Ptab
```

```
##          Chi-Squared Fisher
## original      0.0290 0.0272
## rarefied      0.1171 0.1169
```

And here is how to translate that matrix into LaTeX table format:

```
library("xtable")
xtable(Ptab, digits = 4)
```

```
## % latex table generated in R 3.0.0 by xtable 1.7-1 package
## % Thu Aug 22 18:18:11 2013
## \begin{table}[ht]
## \centering
## \begin{tabular}{rrr}
##   \hline
##  & Chi-Squared & Fisher \\ 
##   \hline
## original & 0.0290 & 0.0272 \\ 
##   rarefied & 0.1171 & 0.1169 \\ 
##    \hline
## \end{tabular}
## \end{table}
```

Regardless of the formatting, you can see from this table that the rarefied version of the data is no longer able to differentiate between the two samples. This is a loss of power induced solely by decreasing the sample size, which in-turn increases the confidence intervals corresponding to each proportion.

## Rarefying introduces additional sampling noise

The simplest way to show this from our minimal example above is to repeat the rarefying procedure many times, rather than assume (as we did above) that the resulting proportions after rarefying will take their expected value. In practice, rarefying is a random procedure that is usually not repeated. There is no reason to expect that the proportions that resulted from rarefying will exactly match the originals, even in our simplest-case example of a community consisting of just two species in a 50/50 proportion.

```
# The number of times to repeat rarefying.
nreplicates = 100
rarefied = matrix(NA_integer_, nrow = nreplicates, ncol = 2, dimnames = list(1:100, 
    c("OTU1", "OTU2")))
for (i in 1:nreplicates) {
    rarefied[i, ] <- table(sample(c(rep(1, 500), rep(2, 500)), 100, replace = FALSE))
}
hist(rarefied[, "OTU1"])
```

```
hist(rarefied[, "OTU2"])
```

Of course, the average of many repeated rarefying steps approaches the original proportions. However, as you can see in the histogram, there were many trials in which the result of rarefying deviated substantially from the original proportion, and this impacts our statistical inference downstream. Effectively this is added noise that we are artificially adding to the count values.
