# Supplementary material for: Waste Not, Want Not: Why Rarefying Microbiome Data Is Inadmissible
Source: PLoS Comput Biol. 2014 Apr 3;10(4):e1003531. doi: 10.1371/journal.pcbi.1003531 (PMC3974642; doi:10.1371/journal.pcbi.1003531)
Supplement: Protocol S1 — A zip file containing all supplementary source files. This includes the Rmd source code, HTML output, and all related documentation and code to completely and exactly recreate every results figure in this article. (ZIP) [file pcbi.1003531.s001.zip › norarefy-source/simulation-cluster-accuracy/simulation-cluster-accuracy-server.html]

Simulation by Random Subsampling, Comparison of Normalization


# Simulation by Random Subsampling, Comparison of Normalization

---

## Load Required Packages

Clear workspace prior to run.

```
rm(list = ls())
```

```
# The required package list:
reqpkg = c("edgeR", "PoiClaClu", "phyloseq", "DESeq", "foreach", "doParallel", 
    "plyr", "reshape2", "ggplot2", "grid", "scales", "cluster")
# Check against installed packages:
inpkg = installed.packages()[, "Package"]
neededpkg = reqpkg[!reqpkg %in% inpkg]
if (length(neededpkg) > 0) {
    stop(paste("Need to install the following package:", neededpkg))
}
# Load all required packages and show version
for (i in reqpkg) {
    print(i)
    library(i, quietly = TRUE, verbose = FALSE, warn.conflicts = FALSE, character.only = TRUE)
    packageVersion(i)
}
```

```
## [1] "edgeR"
## [1] "PoiClaClu"
## [1] "phyloseq"
## [1] "DESeq"
## [1] "foreach"
## [1] "doParallel"
## [1] "plyr"
## [1] "reshape2"
## [1] "ggplot2"
## [1] "grid"
## [1] "scales"
## [1] "cluster"
```

```
# Show session info
sessionInfo()
```

```
## R version 3.0.2 (2013-09-25)
## Platform: x86_64-unknown-linux-gnu (64-bit)
## 
## locale:
##  [1] LC_CTYPE=en_US.iso885915       LC_NUMERIC=C                  
##  [3] LC_TIME=en_US.iso885915        LC_COLLATE=en_US.iso885915    
##  [5] LC_MONETARY=en_US.iso885915    LC_MESSAGES=en_US.iso885915   
##  [7] LC_PAPER=en_US.iso885915       LC_NAME=C                     
##  [9] LC_ADDRESS=C                   LC_TELEPHONE=C                
## [11] LC_MEASUREMENT=en_US.iso885915 LC_IDENTIFICATION=C           
## 
## attached base packages:
## [1] grid      parallel  methods   stats     graphics  grDevices utils    
## [8] datasets  base     
## 
## other attached packages:
##  [1] cluster_1.14.4     scales_0.2.3       ggplot2_0.9.3.1   
##  [4] reshape2_1.2.2     plyr_1.8           doParallel_1.0.6  
##  [7] iterators_1.0.6    foreach_1.4.1      DESeq_1.14.0      
## [10] locfit_1.5-9.1     Biobase_2.22.0     BiocGenerics_0.8.0
## [13] phyloseq_1.6.0     picante_1.6-1      nlme_3.1-113      
## [16] vegan_2.0-10       lattice_0.20-24    permute_0.8-0     
## [19] ape_3.0-11         ade4_1.6-2         PoiClaClu_1.0.2   
## [22] edgeR_3.4.2        limma_3.18.3       knitr_1.5         
## 
## loaded via a namespace (and not attached):
##  [1] annotate_1.40.0      AnnotationDbi_1.24.0 biom_0.3.11         
##  [4] Biostrings_2.30.1    codetools_0.2-8      colorspace_1.2-4    
##  [7] DBI_0.2-7            dichromat_2.0-0      digest_0.6.3        
## [10] evaluate_0.5.1       formatR_0.10         genefilter_1.44.0   
## [13] geneplotter_1.40.0   gtable_0.1.2         igraph_0.6.6        
## [16] IRanges_1.20.5       labeling_0.2         MASS_7.3-29         
## [19] Matrix_1.1-0         multtest_2.18.0      munsell_0.4.2       
## [22] proto_0.3-10         RColorBrewer_1.0-5   RJSONIO_1.0-3       
## [25] RSQLite_0.11.4       splines_3.0.2        stats4_3.0.2        
## [28] stringr_0.6.2        survival_2.37-4      tools_3.0.2         
## [31] XML_3.98-1.1         xtable_1.7-1         XVector_0.2.0
```

Load some theme parameters for ggplot2.

```
theme_set(theme_bw())
pal = "Set1"
scale_colour_discrete <- function(palname = pal, ...) {
    scale_colour_brewer(palette = palname, ...)
}
scale_fill_discrete <- function(palname = pal, ...) {
    scale_fill_brewer(palette = palname, ...)
}
```

---

## Set parameters for this simulation

```
set.seed(20140206)
savedatafile = "simulation-cluster-accuracy"
# load(savedatafile)
sampletypes = c("Feces", "Ocean")

# Number of OTUs in simulation.  This is the maximum.  For less-diverse
# template, or simulated samples with fewer reads, the actual number of OTUs
# will be much less.
nOTUs = 2000L
# nOTUs = 603L

# Minimum number of reads to consider an OTU 'observed' in a sample
minobs = 1L

# Samples per simulation
J = 40L
# J = 7L

# Effect Size. The artificial mix fraction.
mixfacs = c(1, 1.15, 1.25, 1.5, 1.75, 2, 2.5, 3.5)
# mixfacs = c(1, 1.25, 2)

ns = c(1000, 2000, 5000, 10000)
# ns = c(1000, 50000)

# Vector of the replicate numbers to repeat for each comb of simulation
# parameters (n, etc)
reps = 1:5
# reps=1:2

# The delimiter for command parameters
comdelim = "_"

# Number of cores. Only relevent for certain types of back-end registration.
Ncores = 8
# Ncores = 7

# rarefying power params
rarefy_fracs = c(0, seq(0.05, 0.25, 0.05), 0.4)
# rarefy_fracs = c(0, 0.4)

rarereps = 1:2
# rarereps = 1

# The combinations of simulation parameters, used later in the script.
simparams = apply(expand.grid(ns, reps, mixfacs), 1, paste0, collapse = comdelim)
# Define date-stamp for file names
datestamp = gsub(":", "_", gsub("[[:space:]]+", "-", date()), fixed = TRUE)
print(datestamp)
```

```
## [1] "Thu-Feb-6-11_42_48-2014"
```

## Parameter definitions

The major factors contributing to the computation cost in this simulation example are the number of OTUs retained from the template `GlobalPatterns` dataset, which ultimately is used to dictate the length of the multinomials and their corresponding proportion vectors specified by \(\pi\) (R variable `pi` or `pis`); and the number of samples being simulated.

- `nOTUs` – the number of most-prevalent OTUs to keep in simulation template, 2000.
- `minobs` – The minimum abundance value, 1, for which an OTU is “counted” as having been observed in a given sample. This is used for ranking OTUs according to the number of samples in which they appeared.
- `J` – The number of samples per simulated table. For multitable analysis this needs to be consistent across tables, so included here in the beginning as a global parameter. Value for this simulation is 40.
- `mixfacs` – The fold-weighting of the template multinomial compared with its mixed-in counterpart. Same in both directions (symetric mixing).
- `ns` – A vector of the expected values for the sampling depth that are nevertheless subject to the random sampling from the original template totals. The read numbers should not exceed the total number of reads in the template, so this is checked in the “simulation” (subsampling) module, and a ceiling used. The values for this vector of sampling depth means in this particular simulation is: 1000, 2000, 5000, 104.

---

# Define template

Trim to just those samples that are intended as template in this binary effect.

```
data("GlobalPatterns")
sampsums = sample_sums(GlobalPatterns)
keepsamples = sample_data(GlobalPatterns)$SampleType %in% sampletypes
template = prune_samples(keepsamples, GlobalPatterns)
```

Trim OTUs that do not appear in very many samples in the template. Sort by prevalance (number of samples appeared) and then by abundance value.

```
samobs = apply(otu_table(template), 1, function(x, m) sum(x > m), m = minobs)
otudf = data.frame(prev = samobs, sums = taxa_sums(template))
otudf = otudf[order(-otudf$prev, -otudf$sums), ]
```

Trim all but the first nOTUs

```
template = prune_taxa(rownames(otudf)[1:nOTUs], template)
template
```

```
## phyloseq-class experiment-level object
## otu_table()   OTU Table:         [ 2000 taxa and 7 samples ]
## sample_data() Sample Data:       [ 7 samples by 7 sample variables ]
## tax_table()   Taxonomy Table:    [ 2000 taxa by 7 taxonomic ranks ]
## phy_tree()    Phylogenetic Tree: [ 2000 tips and 1999 internal nodes ]
```

---

# Simulate microbiome census

In a previous version I used the `simPop` function from the dirmult package.

Instead, here I am randomly subsampling from the original templates at different numbers of total samples.

## Define templates

```
template1 = subset_samples(template, SampleType == sampletypes[1])
template2 = subset_samples(template, SampleType == sampletypes[2])
```

## Forced mixing.

In the original version 4 result, it appeared that there was very little overlap between the two template types. This could easily be the case, even though they were both body habitats. One biologically intuitive approach would be to choose a different pair of template sample types that are more similar. However, this doesn't afford much additional intuition about the extent that they are similar (without a bunch of extra exploratory analysis), and doesn't give us very much control over the extent that they are similar… we'd be stuck with whatever similarities are “available” among the sample types. Note that it might also help to increase the number of OTUs being included in template and simulation. At the moment it is 2000.

Instead, we will intentionally mix two templates that otherwise have very few OTUs in common, now labeled `template1` and `template2`, by adding a known but small proportion of each template to the other.

```
# Define function for mixing
mix_microbes = function(template1, template2, unmixfac) {
    require("phyloseq")
    # Check that the number of taxa are equal
    if (!identical(ntaxa(template1), ntaxa(template2))) {
        stop("number of OTUs b/w template1 and template2 must be equal")
    }
    # Expects templates to be a 1-sample dataset.
    if (!identical(nsamples(template1), 1L)) {
        stop("`template1` should have only 1 sample")
    }
    if (!identical(nsamples(template2), 1L)) {
        stop("`template2` should have only 1 sample")
    }
    # Enforce taxa_are_rows to FALSE
    if (taxa_are_rows(template1)) {
        template1 <- t(template1)
    }
    if (taxa_are_rows(template2)) {
        template2 <- t(template2)
    }
    # Define a vector version of each template for subsampling
    x1 = as(otu_table(template1), "numeric")
    x2 = as(otu_table(template2), "numeric")
    # Create 'dirty' multinomial.  Defined artificial mixing.  Create mixed
    # multinomial by adding counts from the other in precise proportion, a total
    # of Library Size / Effect Size
    addToTemplate1 = round((sum(x1) * x2/(sum(x2) * unmixfac)), 0)
    # Add them together to create 'dirty' multinomial This adds the fractional
    # subsampled abundances to the template
    mat1 = matrix((addToTemplate1 + x1), nrow = 1)
    rownames(mat1) <- sample_names(template1)
    colnames(mat1) <- taxa_names(template1)
    otu_table(template1) <- otu_table(mat1, taxa_are_rows = FALSE)
    return(template1)
}
# merge the template components together into one sample.
template1 = merge_samples(template1, "SampleType")
template2 = merge_samples(template2, "SampleType")
```

---

## Simulation Function

- Input
  - `postfix`, `template`, `J`, `n` (number or reads)
- Output
  - phyloseq object, incorporating simulation results and inputs

```
microbesim = function(postfix = "sim", template, templatex, unmixfac, J, n = 10000) {
    # Generate `J` simulated microbiomes with `n` total reads each (all the
    # same, or n has length equal to the value of `J`), with subsamples drawn
    # from `template`.  `postfix` is a dummy idenitifer added to help
    # distinguish simulated samples in downstream code.
    require("phyloseq")
    # Perform the mixing here, so each replicate is a separate random mix
    template = mix_microbes(template, templatex, unmixfac)
    # call the proporitions vector `pi`, similar to nomenclature from DMN
    pi = taxa_sums(template)
    # n must be a scalar (recycled as the number of reads for every simulation)
    # or it can be vector of length equal to J, the number of samples being
    # simulated.
    if (length(J) != 1) {
        stop("Length of J should be 1.")
    }
    if (length(n) != 1 & length(n) != J) {
        stop("n should be length 1, or length J.")
    }
    # Actually create the simulated abundance table
    simat = mapply(function(i, x, sample.size) {
        if (FALSE) 
            {
                print(i)
            }  # i is a dummy iterator
        phyloseq:::rarefaction_subsample(x, sample.size)
    }, i = 1:J, sample.size = n, MoreArgs = list(x = pi), SIMPLIFY = TRUE)
    simat = t(simat)
    # Add the OTU names to the OTU (column) indices
    colnames(simat) <- names(pi)
    # Add new simulated sample_names to the row (sample) indices
    rownames(simat) <- paste(i, "::", 1:nrow(simat), postfix, sep = "")
    # Put simulated abundances together with metadata as a phyloseq object
    OTU = otu_table(simat, taxa_are_rows = FALSE)
    # Define data.frame that will become sample_data
    SDF = data.frame(sample = sample_names(OTU), TableNumber = i, type = "simulated", 
        stringsAsFactors = FALSE)
    SDF$postfix <- postfix
    rownames(SDF) <- sample_names(OTU)
    SD = sample_data(SDF)
    # Return a phyloseq object
    return(phyloseq(OTU, SD))
}
```

Define a function for sampling from the library sizes as a way of including the “noise” derived from this aspect of the data as well.

```
# rescale the sum of reads in the original raw(-ish) template data to the
# expected library size being requested here
sumsim = function(n, sumtemplate, J) {
    # `n` - expected size target `sumtemplate` - the template vector of library
    # sizes observed in template `J` - The number of sample sizes to return
    # sumtemplate = sampsums n = 2000 J = 103
    scaledSums = round(n * (sumtemplate/median(sumtemplate)))
    return(sample(scaledSums, size = J, replace = TRUE))
}
```

---

## Register parallel backend for computing

Register parallel clusters for parallel calculations (e.g. UniFrac, etc.).

```
# makeCluster makeForkCluster()

# The parallel single-node way
cl <- makeCluster(Ncores)

# The multi-node way for PSOCK cluster, from:
# http://www.bioconductor.org/help/bioconductor-cloud-ami/#parallel
# library(parallel) lines <- readLines('/usr/local/Rmpi/hostfile.plain')
# hosts <- character() for (line in lines){ x <- (strsplit(line[[1]], ' '))
# hosts <- c(hosts, rep.int(x[[1]][1], as.integer(x[[1]][2]))) } cl <-
# makePSOCKcluster(hosts, master=system('hostname -i', intern=TRUE)) #
# system.time(clusterCall(cl, Sys.sleep, 1)) # A test

# Register the parallel cluster
registerDoParallel(cl)

# The Rmpi/doMPI way (doMPI package should have been loaded already at the
# beginning) cl <- startMPIcluster(verbose=TRUE) registerDoMPI(cl)
```

---

## Simulate microbiome samples from different 'dirty' templates

Repeat the simulation many times, with different values for the number of reads per sample.

```
# Parallelized simulations
simlist <- foreach(i = simparams, .packages = c("phyloseq")) %dopar% {
    # i = simparams[4] Initialize
    n = sim = sim1 = sim2 = n1 = n2 = NULL
    # cat(i, '\n')
    n = as.numeric(strsplit(i, comdelim)[[1]][1])
    mixfac = as.numeric(strsplit(i, comdelim)[[1]][3])
    # Rarely a simulation has a weird value and fails.  Catch these with `try`,
    # and repeat the simulation call if error (it will be a new seed)
    tryAgain = TRUE
    infiniteloopcounter = 1
    while (tryAgain & infiniteloopcounter < 5) {
        n1 = sumsim(n, sampsums, J)
        n2 = sumsim(n, sampsums, J)
        sim1 = microbesim(sampletypes[1], template1, template2, mixfac, J, n1)
        sim2 = microbesim(sampletypes[2], template2, template1, mixfac, J, n2)
        if (is.null(sim1) | is.null(sim2) | is.null(n1) | is.null(n2) | inherits(sim1, 
            "try-error") | inherits(sim2, "try-error")) {
            tryAgain = TRUE
            infiniteloopcounter = infiniteloopcounter + 1
        } else {
            tryAgain = FALSE
        }
    }
    if (infiniteloopcounter >= 5) {
        stop("Consistent error found during simulation. Need to investigate cause.")
    }
    # Merge the two simulated datasets together into one phyloseq object and add
    # back tree.
    sim = merge_phyloseq(sim1, sim2)
    sim = merge_phyloseq(sim, tax_table(GlobalPatterns), phy_tree(GlobalPatterns))
    return(sim)
}
names(simlist) <- simparams
```

---

# Evaluate clustering after different normalizations, metrics

This is the point of this analysis. Clustering method must be the same (and a good method) for all versions of the simulated dataset.

The following sections define functions that normalize the samples to equal numbers of reads per sample in different ways.

## Naïve proportions

Need to “preprocess” the simulated microbiome data. Adapted code on standardizing/normalizing/centering(not supported) data. **Rm zeros** Remove the zeros. That is, remove any OTUs with a zero-sum across all samples. Hopefully not many. Also do the same for simulated samples. Both could cause problems later. **Normalize**. Normalize sampling effort of each sample. This won't correct for unobserved OTUs, which will still be zero, but an OTU trimming step helps correct that. **Scale**. Scale by dividing each variable by its standard deviation. **Center**. Center by subtracting the median of each sample from each element of that sample. Skip this for now. The phyloseq package currently forbids negative abundance values. Maybe this should be changed to a warning rather than an error in the package (or get rid of that condition)…

We can take as a given that samples or OTUs that are not reprsented in datasets (did not get any simulated reads), would be cut in any preprocessing/normalization method.

```
# Rm any samples or OTUs that don't have any reads.  Also remove singletons
# (only 1 count as sample or OTU)
remove_singletons_empties = function(physeq) {
    require("phyloseq")
    physeq = prune_taxa(taxa_sums(physeq) > 2.5, physeq)
    physeq = prune_samples(sample_sums(physeq) > 2.5, physeq)
    # Remove OTUs not appearing in at least 3 samples
    if (taxa_are_rows(physeq)) {
        y = otu_table(physeq)
        wh1 = apply(aaply(y, 1, function(x) {
            x >= 1
        }), MARGIN = 1, sum) >= 3
    } else {
        y = as(t(otu_table(physeq)), "matrix")
        wh1 = apply(aaply(y, 1, function(x) {
            x >= 1
        }), MARGIN = 1, sum) >= 3
    }
    physeq = prune_taxa(wh1, physeq)
    return(physeq)
}
# Remove singletons and empties
simnames = names(simlist)
simlist = foreach(physeq = simlist, .packages = c("phyloseq", "plyr")) %dopar% 
    {
        remove_singletons_empties(physeq)
    }
names(simlist) <- simnames
```

Define the naïve (simple proportion) normalization function.

```
proportion = function(physeq) {
    # Normalize total sequences represented
    normf = function(x, tot = max(sample_sums(physeq))) {
        tot * x/sum(x)
    }
    physeq = transform_sample_counts(physeq, normf)
    # Scale by dividing each variable by its standard deviation. physeq =
    # transform_sample_counts(physeq, function(x) x/sd(x)) Center by subtracting
    # the median physeq = transform_sample_counts(physeq, function(x)
    # (x-median(x)))
    return(physeq)
}
```

### Random subsampling

Remove some of the lowest-abundance samples (e.g. poorest-represented quartile of samples) prior to random subsampling.

```
randomsubsample = function(physeq, smalltrim = 0.15, replace = TRUE) {
    require("phyloseq")
    # Set the minimum value as the smallest library quantile, n`smalltrim`
    samplemin = sort(sample_sums(physeq))[-(1:floor(smalltrim * nsamples(physeq)))][1]
    physeqr = rarefy_even_depth(physeq, samplemin, rngseed = FALSE, replace = replace, 
        trimOTUs = TRUE)
    return(physeqr)
}
```

### edgeR normalization

Normalizations provided by the edgeR pacakge. Most notably, “TMM” normalization.

```
edgeRnorm = function(physeq, ...) {
    require("edgeR")
    require("phyloseq")
    # physeq = simlist[['55000_1e-04']] z0 = simlisttmm[['55000_1e-04']] physeq
    # = simlist[['1000_0.2']] z0 = simlisttmm[['1000_0.2']] Enforce orientation.
    if (!taxa_are_rows(physeq)) {
        physeq <- t(physeq)
    }
    x = as(otu_table(physeq), "matrix")
    # See if adding a single observation, 1, everywhere (so not zeros) prevents
    # errors without needing to borrow and modify calcNormFactors (and its
    # dependent functions) It did. This fixed all problems.  Can the 1 be
    # reduced to something smaller and still work?
    x = x + 1
    # Now turn into a DGEList
    y = edgeR::DGEList(counts = x, remove.zeros = TRUE)
    # Perform edgeR-encoded normalization, using the specified method (...)
    z = edgeR::calcNormFactors(y, ...)
    # A check that we didn't divide by zero inside `calcNormFactors`
    if (!all(is.finite(z$samples$norm.factors))) {
        stop("Something wrong with edgeR::calcNormFactors on this data, non-finite $norm.factors")
    }
    # Don't need the following additional steps, which are also built-in to some
    # of the downstream distance methods. z1 = estimateCommonDisp(z) z2 =
    # estimateTagwiseDisp(z1)
    return(z)
}
```

### DESeq normalization

From DESeq:
“The inference in DESeq relies on an estimation of the typical relationship between the data’s variance and their mean, or, equivalently, between the data’s dispersion and their mean. The dispersion can be understood as the square of the coefficient of biological variation. So, if a gene’s expression typically differs from replicate to replicate sample by 20%, this gene’s dispersion is 0.22 = .04. Note that the variance seen between counts is the sum of two components: the sample-to-sample variation just mentioned, and the uncertainty in measuring a concentration by counting reads. The latter, known as shot noise or Poisson noise, is the dominating noise source for lowly expressed genes. The former dominates for highly expressed genes. The sum of both, shot noise and dispersion, is considered in the differential expression inference. Hence, the variance v of count values is modelled as:

v = sμ + αs2μ2

\[
\upsilon = s\mu + \alpha{s}^2{\mu}^2
\]

where $\mu is the expected normalized count value (estimated by the average normalized count value), \(s\) is the size factor for the sample under consideration, and \(\alpha\) is the dispersion value for the gene under consideration.
”

To estimate the dispersions, use the `estimateDispersions` function. Note that there are `method`, `sharingMode`, and `fitType` arguments to `estimateDispersions`, the choice of which are presumed to affect power. For the sample clustering simulation here, we have no “replicate” samples from a particular treatment or technical step.

```
deseq_varstab = function(physeq, sampleConditions = rep("A", nsamples(physeq)), 
    ...) {
    require("DESeq")
    # Enforce orientation.
    if (!taxa_are_rows(physeq)) {
        physeq <- t(physeq)
    }
    x = as(otu_table(physeq), "matrix")
    # The same tweak as for edgeR to avoid NaN problems that cause the workflow
    # to stall/crash.
    x = x + 1
    # Create annotated data.frame with the taxonomy table, in case it is useful
    # later
    taxADF = as(data.frame(as(tax_table(physeq), "matrix"), stringsAsFactors = FALSE), 
        "AnnotatedDataFrame")
    cds = newCountDataSet(x, sampleConditions, featureData = taxADF)
    # First estimate library size factors
    cds = estimateSizeFactors(cds)
    # Variance estimation, passing along additional options
    cds = estimateDispersions(cds, ...)
    # Determine which column(s) have the dispersion estimates
    dispcol = grep("disp\\_", colnames(fData(cds)))
    # Enforce that there are no infinite values in the dispersion estimates
    if (any(!is.finite(fData(cds)[, dispcol]))) {
        fData(cds)[which(!is.finite(fData(cds)[, dispcol])), dispcol] <- 0
    }
    vsmat = exprs(varianceStabilizingTransformation(cds))
    otu_table(physeq) <- otu_table(vsmat, taxa_are_rows = TRUE)
    return(physeq)
}
```

From DESeq vignette:

“As we estimated the dispersions from a small number of replicates, the estimates scatter with quite some sampling variance around their true values. An initial assumption that one could make is that the regression line shown in Figure 1 models the true underlying dispersions, and that the variation of the point estimates around simply reflects sampling variance. This is the assumption that we put forward in the first paper on DESeq. However, subsequent experience with larger data sets indicates that not all of the variability of the points around the regression line seen in Figure 1 is sampling variance: some of it reflects differences of the true, underlying variance between different genes. Hence, the default behaviour of DESeq now uses a more prudent or conservative approach: if a per-gene estimates lies below the regression line, we assume that this might indeed be sampling variance, and shift the estimate upwards to the value predicted by the regression line. If, however, the per-gene estimate lies above the line, we do not shift it downwards to the line, but rather keep it as is.

The option `sharingMode` of the function estimateDispersions can be used to control this behaviour. The value `sharingMode="maximum"` corresponds to the default. If you have many replicates (where many starts at around 7), the choice `sharingMode="gene-est-only"` will typically be more adequate. If you would like to use the behaviour described in [the original DESeq article], this is achieved by specifying `sharingMode="fit-only"`.

Another difference of the current DESeq version to the original method described in the paper is the way how the mean-dispersion relation is fitted. By default, estimateDispersions now performs a parametric fit: Using a gamma-family GLM, two coefficients \(\alpha\_{0}\), \(\alpha\_{1}\) are found to parametrize the fit as \(\alpha = \alpha\_{0} + \alpha\_{1}/\mu\). (The values of the two coefficients can be found in the `fitInfo` object, as attribute coefficients to `dispFunc`.) For some data sets, the parametric fit may give bad results [or just fail], in which case one should try a local fit (the method described in the paper), which is available via the option `fitType="local"` to `estimateDispersions`.

In any case, the dispersion values which will be used by the subsequent testing are stored in the feature data slot of cds [use `fData`].”

To adjust the dispersion estimates, fix infinite values, use the `fData` accessor prior to calling the testing function.

### Perform normalizations

```
simpletrim = function(physeq, J) {
    if (taxa_are_rows(physeq)) {
        physeq = t(physeq)
    }
    # `prevalence` is the fraction of total samples in which an OTU is observed
    # at least `minobs` times.
    prevalence = apply(as(otu_table(physeq), "matrix"), 2, function(x, minobs) {
        sum(x > minobs)
    }, minobs)/(2 * J)
    # Will only keep OTUs that appear in more than X% of samples
    keepOTUs = prevalence > 0.05
    # Keep only those OTUs with total reads greater than 0.5x the number of
    # samples.
    keepOTUs = keepOTUs & taxa_sums(physeq) > (0.5 * J)
    return(prune_taxa(keepOTUs, physeq))
}
```

Perform initializations, and trimming

```
# Initialize the simulation lists simlist0 and simlist
simlist0 = simlistuntrim = simlist
# checks apply(as(otu_table(simlistuntrim[[1]]), 'matrix'), 2, function(x,
# tot){sum(x>tot)}, minobs)/(2*J) sapply(lapply(simlistuntrim, taxa_sums),
# function(x, J){sum(x < (0.5*J))}, J)/nOTUs Trim simlist0
simlist0 = lapply(simlistuntrim, simpletrim, J)
```

Now perform the normalizations.

```
# Replace simlist with empty list. Will add different norm types to it.
simlist = vector("list", 0)
simlist$none <- simlist0

# Define the 'naive proportion' list
simlist$naiveProp <- foreach(physeq = simlist0, .packages = "phyloseq") %dopar% 
    {
        proportion(physeq)
    }
names(simlist$naiveProp) <- names(simlist0)

# rarefying.
simlist$rarefy <- foreach(physeq = simlist0, .packages = "phyloseq") %dopar% 
    {
        randomsubsample(physeq, smalltrim = 0.15, replace = FALSE)
    }
names(simlist$rarefy) <- names(simlist0)

# edgeR norms
simlist$edgeRTMM <- foreach(physeq = simlist0, .packages = c("phyloseq", "edgeR")) %dopar% 
    {
        edgeRnorm(physeq, method = "TMM")
    }
names(simlist$edgeRTMM) <- names(simlist0)
simlist$edgeRRLE <- foreach(physeq = simlist0, .packages = c("phyloseq", "edgeR")) %dopar% 
    {
        edgeRnorm(physeq, method = "RLE")
    }
names(simlist$edgeRRLE) <- names(simlist0)
simlist$edgeRupqua <- foreach(physeq = simlist0, .packages = c("phyloseq", "edgeR")) %dopar% 
    {
        edgeRnorm(physeq, method = "upperquartile")
    }
names(simlist$edgeRupqua) <- names(simlist0)

# DESeq variance stabilizing transformations
simlist$DESeqVS <- foreach(physeq = simlist0, .packages = c("phyloseq", "DESeq")) %dopar% 
    {
        deseq_varstab(physeq, method = "blind", sharingMode = "maximum", fitType = "local")
    }
names(simlist$DESeqVS) <- names(simlist0)
```

## Save list of normalized simulations

```
save.image(paste0("simulations-", datestamp, ".RData"))
```

---

## Calculate and Evaluate distances

Use `cluster::pam` (or some other distance clustering algorithm) on each distance matrix with `k=2`, and then evaluate results. Repeat for each value of `n` (number of reads) and each normalization approach. Plot the results (error rate versus number of reads).

### Leading Log-Fold change

Define a sample-wise distance function from tools in limma and edgeR packages, especially the `plotMDS` variants in those two packages. This includes the “leading log fold-change” distance used in Witten et al., and others.

```
################################################################################ Distance function adapted for this analysis Expects a DGEList as main
################################################################################ input.  Can calculate sample-wise distances for both 'biological
################################################################################ coefficient of variation' or the lead log fold change ('logFC') distance
################################################################################ using 'pairwise'' or 'common' `gene.selection` methods.  From
################################################################################ edgeR::plotMDS.DGEList: 'Distances on the plot represent coefficient of
################################################################################ variation of expression between samples for the top genes that best
################################################################################ distinguish the samples' root mean squared difference for top features.
################################################################################ (of the logFC transformed counts, via edgeR::cpm) Root mean squared
################################################################################ difference of the logFC transformed counts of the top OTUs only.  Adapted
################################################################################ from edgeR::plotMDS.DGEList Multidimensional scaling plot of digital gene
################################################################################ expression profiles Yunshun Chen, Mark Robinson and Gordon Smyth 23 May
################################################################################ 2011.  Last modified 7 Feb 2013.
edgeRdist <- function(x, top = 500, method = "logFC", gene.selection = "pairwise", 
    prior.count = 2) {
    require("edgeR")
    require("phyloseq")
    if (inherits(x, "phyloseq")) {
        # Convert phyloseq object to an otherwise untransformed abundance matrix in
        # DGEList form
        x = edgeRnorm(x, method = "none")
        if (!inherits(x, "DGEList")) {
            stop("x not converted to DGEList for some reason. Dispatch now flawed.")
        }
    }
    # Remove rows with missing or Inf values
    ok <- is.finite(x$counts)
    if (!all(ok)) {
        x <- x[rowSums(ok) > 0, ]
        x$samples$lib.size <- rowSums(x$counts)
    }
    nprobes <- nrow(x)
    nsamples <- ncol(x)
    # Check that `top` exceeds `nprobes` by at least a small margin.  Give
    # warning if you have to change it.
    if (top > (0.8 * nprobes)) {
        warning("The value of `top` was ", top, " for a dataset with ", nprobes, 
            "probes. \n Decreasing `top` to ", round(0.8 * nprobes), " probes.")
        top = 0.8 * nprobes
    }

    # Initialize distance object, dd
    cn = colnames(x)
    dd = matrix(0, nrow = nsamples, ncol = nsamples, dimnames = list(cn, cn))
    ######################################## Default method is to convert to moderated logCPM and call limma plotMDS
    method <- match.arg(method, c("logFC", "bcv", "BCV"))
    if (method == "logFC") {
        y <- edgeR::cpm(x, log = TRUE, prior.count = prior.count)
        # Here we're essentially dispatching to code derived from the limma::plotMDS
        # function, but only the dist-calculating portion.

        # Check top. `top` is handled differently between logFC and bcv
        top <- min(top, nprobes)
        # Check gene.selection
        gene.selection <- match.arg(gene.selection, c("pairwise", "common"))
        # Distance matrix from pairwise leading fold changes
        dd <- matrix(0, nrow = nsamples, ncol = nsamples, dimnames = list(cn, 
            cn))
        topindex <- nprobes - top + 1
        if (gene.selection == "pairwise") {
            # Distance measure is mean of top squared deviations for each pair of arrays
            for (i in 2:(nsamples)) for (j in 1:(i - 1)) {
                dd[i, j] <- sqrt(mean(sort.int((y[, i] - y[, j])^2, partial = topindex)[topindex:nprobes]))
            }
        } else {
            # gene.selection == 'common'.  Same genes used for all comparisons
            s <- rowMeans((y - rowMeans(y))^2)
            q <- quantile(s, p = (topindex - 1.5)/(nprobes - 1))
            y <- y[s >= q, ]
            for (i in 2:(nsamples)) {
                dd[i, 1:(i - 1)] <- sqrt(colMeans((y[, i] - y[, 1:(i - 1), drop = FALSE])^2))
            }
        }
        return(as.dist(dd))
    }
    ######################################## Code past this point is for method='bcv'
    x$samples$group <- factor(rep.int(1, nsamples))
    # Check value for top
    if (top < nprobes) {
        twd <- edgeR::estimateTagwiseDisp(edgeR::estimateCommonDisp(x), grid.length = 100)
        o <- order(twd$tagwise.dispersion, decreasing = TRUE)[1:top]
        subdata <- x$counts[o, , drop = FALSE]
    } else {
        subdata <- x$counts
    }

    lib.size <- x$samples$lib.size * x$samples$norm.factors
    myFun <- function(delta, y, der = 1) {
        return(sum(condLogLikDerDelta(y, delta, der)))
    }

    for (i in 2:(nsamples)) {
        for (j in 1:(i - 1)) {
            mm <- subdata[, c(i, j)]
            rs5 <- rowSums(mm) > 5
            lib <- lib.size[c(i, j)]
            norm <- t(t(mm)/lib) * exp(mean(log(lib)))
            delta <- optimize(myFun, interval = c(1e-04, 0.99), tol = 1e-06, 
                maximum = TRUE, y = norm[rs5, ], der = 0)
            dd[i, j] = sqrt(delta$maximum/(1 - delta$maximum))
        }
    }
    return(as.dist(dd))
}
################################################################################
```

The above function requires a `"DGEList"` class, which you will have already created during your TMM normalization step.

Add small tweak to `PoissonDistance`, so that it handles phyloseq data.

```
PoissonDist = function(physeq, ...) {
    # PoiClaClu expects samples as rows and taxa/variables as columns
    if (taxa_are_rows(physeq)) {
        physeq <- t(physeq)
    }
    x = as(otu_table(physeq), "matrix")
    dd = PoiClaClu::PoissonDistance(x, ...)$dd
    attributes(dd)$Labels <- sample_names(physeq)
    return(dd)
}
```

### DESeq variance stabilization and distance calculation

```
deseqdistwrap = function(physeq, ...) {
    require("phylosq")
    require("DESeq")
    physeqVS = deseq_varstab(physeq, method = "blind", sharingMode = "maximum", 
        fitType = "local")
    return(distance(physeqVS, ...))
}
```

### Distance commands

Calculate UniFrac and other distances. This works by defining a large vector of complete commands for calculating sample-wise distances. This large vector will be split up as “embarassingly parallelizable” jobs for `foreach() %dopar% {}`.

```
# naive proportion
distcommands = c(
  paste("simlist$naiveProp", names(simlist$naiveProp), 
        "distance", "'unifrac'", sep=comdelim),
  paste("simlist$naiveProp", names(simlist$naiveProp), 
        "distance", "'unifrac'", "weighted=TRUE", sep=comdelim),
    paste("simlist$naiveProp", names(simlist$naiveProp), 
        "distance", "'bray'", sep=comdelim)
)
# rarefy - random subsample
distcommands = c(distcommands,
    paste("simlist$rarefy", names(simlist$rarefy), 
        "distance", "'unifrac'", sep=comdelim),
    paste("simlist$rarefy", names(simlist$rarefy), 
        "distance", "'unifrac'", "weighted=TRUE", sep=comdelim),
    paste("simlist$rarefy", names(simlist$rarefy), "distance", "'bray'", sep=comdelim)
)
# edgeR normalized
# Define groups of normalizations/parameters to use
edgeRnormtypes = c("simlist$edgeRTMM", "simlist$edgeRRLE", 
                   "simlist$edgeRupqua", #"simlist$edgeRnone",
                                     "simlist$rarefy")
edgeRmethtypes = c("method='logFC'", "method='bcv'")
#edgeRgenetypes = c("gene.selection='pairwise'", "gene.selection='common'")
edgeRgenetypes = c("gene.selection='pairwise'")
edgeRtopvalues = c("top=2000")
edgeRmatcoms = expand.grid(edgeRnormtypes, names(simlist$edgeRTMM), "edgeRdist",
                           edgeRmethtypes, edgeRgenetypes, edgeRtopvalues)
edgedistcoms = apply(edgeRmatcoms, 1, paste0, collapse=comdelim)
distcommands = c(distcommands, edgedistcoms)

# DESeq. Using DESeq directly, not via PoiClaClu
# Define groups of normalizations/parameters to expand
#### "simlist$DESeqFit", "simlist$DESeqGene")
DESeqnormtypes = c("simlist$none", "simlist$DESeqVS", "simlist$rarefy")
DESeqdisttypes = c("method='euclidean'", "method='unifrac', weighted=TRUE", "method='bray'")
DESeqmatcoms = expand.grid(DESeqnormtypes, names(simlist$DESeqVS),
                                                     "distance", DESeqdisttypes)
DESeqdistcoms = apply(DESeqmatcoms, 1, paste0, collapse=comdelim)
distcommands = c(distcommands, DESeqdistcoms)

# PoiClaClu "normalized"... built-in to the model used in the distance function:
PoiClaCluNorms = c("simlist$none", "simlist$rarefy")
PoiClaCluDists = c("type='mle'", "type='deseq'", "type='quantile'")
PoiClaClumatcoms = expand.grid(PoiClaCluNorms, names(simlist$none),
                                                 "PoissonDist", PoiClaCluDists)
PoiClaCludistcoms = apply(PoiClaClumatcoms, 1, paste0, collapse=comdelim)
distcommands = c(distcommands, PoiClaCludistcoms)
```

Calculate distances using the vector of `"_"`-delimited commands above.

```
require("foreach")
dlist <- foreach(dcom = distcommands, .packages = c("phyloseq", "PoiClaClu", 
    "edgeR"), .export = c("simlist", "edgeRnorm", "edgeRdist", "PoissonDist", 
    "deseq_varstab")) %dopar% {

    cmdsplit = strsplit(dcom, split = comdelim, fixed = TRUE)[[1]]
    physeq = eval(parse(text = cmdsplit[1]))[[paste0(cmdsplit[2:4], collapse = comdelim)]]
    if (length(cmdsplit) == 5) {
        dd = do.call(cmdsplit[5], physeq)
    } else if (length(cmdsplit) > 5) {
        fcall = paste0(cmdsplit[5], "(physeq, ", paste(cmdsplit[6:length(cmdsplit)], 
            collapse = ", "), ")")
        dd = eval(parse(text = fcall))
    } else {
        stop("split distance command string is unexpected length")
    }
    # Check that dd has Labels, or it will screw up clustering eval downstream
    if (is.null(attributes(dd)$Labels)) {
        attributes(dd)$Labels <- sample_names(physeq)
    }
    return(dd)
}
names(dlist) <- distcommands
all(sapply(dlist, class) == "dist")
```

```
## [1] TRUE
```

`NA` distances. So far, it looks like it is very rare for any distance matrices to have `NA` values, but if it does happen it makes `pam` fail. The reason for this happening is unclear right now, but appears constrained to the `PoissonDistance` results. My first guess is that these simulated samples have nothing in common, and so `PoissonDistance` gives them a value of `NA` rather than infinity (or 1, if it is normalized distance). Instead of removing these distance matrices (which is one option for this simulation), since this is a rare event, I will instead give these entries the max value that otherwise occurred in the distance matrix.

```
# Define function to Replace infinite or NA values in distance matrices with
# 1.25x the max value
fixbadDs = function(dlist) {
    lapply(dlist, function(dd) {
        mx = max(dd, na.rm = TRUE)
        mx = 1.25 * mx
        if (any(is.infinite(dd))) {
            dd[is.infinite(dd)] <- mx
        }
        if (any(is.na(dd))) {
            dd[is.na(dd)] <- mx
        }
        return(dd)
    })
}
dlist = fixbadDs(dlist)
```

Binary clustering using `limma::pam` (partitioning around medoids) with `k==2`, and alternatively `stats::kmeans` and `stats::hclust`.

```
# pam
CLlist <- foreach(dd = dlist, .packages = "cluster") %dopar% {
    pam(dd, k = 2, cluster.only = TRUE)
}
names(CLlist) <- names(dlist)
# kmeans
CLlist_kmeans <- foreach(dd = dlist, .packages = "stats") %dopar% {
    kmeans(cmdscale(dd, 2), 2)$cluster
}
names(CLlist_kmeans) <- names(dlist)
# hclust
CLlist_hclust <- foreach(dd = dlist, .packages = "stats") %dopar% {
    cutree(hclust(dd), k = 2)
}
names(CLlist_hclust) <- names(dlist)
```

Evaluate the results of each clustering.

```
# Evaluate each clustering result in CLlist
binaryclusteval = function(x, tot) {
    # Assumes argument, x, is a named cluster vector with two components, and
    # that the 'true' clustering can be inferred from the name of each element,
    # namely, the non-digit, non-delimiter word in each name.  x = CLlist[[1]]
    # tot = 2*J truth =
    # as(factor(gsub('[[:digit:]]{1}\\:\\:[[:digit:]]{1,}', '', names(x))),
    # 'numeric')
    truth = as(factor(gsub("[[:print:]]+\\:\\:[[:digit:]]{1,}", "", names(x))), 
        "numeric")
    xinv = ifelse(x == 1, 2, 1)
    correct = max(sum(x == truth), sum(xinv == truth))
    return(c(fracCorrectPred = (correct/length(x)), fracCorrect = (correct/tot)))
}
edf = ldply(CLlist, function(x, binaryclusteval, tot) {
    clres = binaryclusteval(x, tot)
    return(data.frame(fracCorrectPred = clres["fracCorrectPred"], fracCorrect = clres["fracCorrect"]))
}, binaryclusteval, tot = (2 * J))
# Add the commands to the first column of edf, with name `fcall`
edf = data.frame(fcall = names(CLlist), edf, stringsAsFactors = FALSE)
# colnames(edf)[1] <- 'fcall'
```

Now parse the clustering results table, `edf`.

```
# Define the normalization type.
edf$normtype <- gsub("(simlist\\$)([[:alnum:]]+)(\\_[[:print:]]+$)", "\\2", 
    edf$fcall)
# Define the expected number of reads included.
edf$nreads <- as(sapply(strsplit(edf$fcall, comdelim, fixed = TRUE), function(x) x[2]), 
    "integer")
# The replicate number
edf$replicate <- as(sapply(strsplit(edf$fcall, comdelim, fixed = TRUE), function(x) x[3]), 
    "numeric")
# The mix factor
edf$mix_factor <- as(sapply(strsplit(edf$fcall, comdelim, fixed = TRUE), function(x) x[4]), 
    "numeric")
# use the remaining info to define the function call
edf$distmethod <- sapply(strsplit(edf$fcall, comdelim, fixed = TRUE), function(x) paste(x[-(1:4)], 
    collapse = ", "))
# Melt on fractionPredicted/fractionTotal so that
require("reshape2")
edf = melt(edf, measure.vars = c("fracCorrectPred", "fracCorrect"))
colnames(edf)[colnames(edf) == "variable"] <- "AccuracyMeasure"
colnames(edf)[colnames(edf) == "value"] <- "Accuracy"
colnames(edf)[colnames(edf) == "normtype"] <- "Normalization"
# Relabel for plotting
colnames(edf)[colnames(edf) == "distmethod"] <- "Distance"
colnames(edf)[colnames(edf) == "mix_factor"] <- "Effect_Size"
# Relabel Distance measure names
edf$Distance <- gsub("distance, 'unifrac', weighted=TRUE", "UniFrac-w", edf$Distance, 
    fixed = TRUE)
edf$Distance <- gsub("distance, method='unifrac', weighted=TRUE", "UniFrac-w", 
    edf$Distance, fixed = TRUE)
edf$Distance <- gsub("distance, method='euclidean'", "Euclidean", edf$Distance, 
    fixed = TRUE)
edf$Distance[grep("distance, 'unifrac'", edf$Distance)] <- "UniFrac-u"
edf$Distance[grep("edgeRdist", edf$Distance)] <- "top-MSD"
edf$Distance[grep("bray", edf$Distance)] <- "Bray-Curtis"
edf$Distance[grep("PoissonDist", edf$Distance)] <- "PoissonDist"
# Relabel normalization methods
edf$Normalization[edf$Normalization == "naiveProp"] <- "Proportion"
edf$Normalization[edf$Normalization == "edgeRupqua"] <- "UQ-logFC"
edf$Normalization[edf$Normalization == "rarefy"] <- "Rarefy"
edf$Normalization[edf$Normalization == "none"] <- "None"
# Add a distance options variable, Options
edf$DistCall <- sapply(strsplit(edf$fcall, comdelim, fixed = TRUE), function(x) paste(x[-(1:4)], 
    collapse = ", "))
edf$Options <- sapply(strsplit(edf$fcall, comdelim, fixed = TRUE), function(x) paste(x[-(1:5)], 
    collapse = ", "))
```

Create main plot. Summarize clustering accuracy performance at different effect sizes, library sizes using different normalization and distance measures. Try comparing across methods and down library size before any subsetting of the results. Select the best subset of parameters to capture the behavior of the different normalization/distance methods in a graphic size that can fit as a main figure. The complete results will be shown in the supplemental version.

```
edfmain = subset(edf, AccuracyMeasure == "fracCorrect")
edfmain = subset(edfmain, nreads %in% c(1000, 5000))
edfmain = subset(edfmain, !Distance %in% c("UniFrac-u"))
# Subset to the best for each panel (Distance, nreads, Normalization)
edfmain = ddply(edfmain, c("Distance", "nreads", "Normalization"), function(df) {
    panelmeans = daply(df, "Options", function(x) {
        mean(x$Accuracy)
    })
    if (length(panelmeans) > 1) {
        df = df[df$Options == names(panelmeans[which.max(panelmeans)]), ]
    }
    # else leave as-is
    return(df)
})
```

Graphically summarize error between different normalization/distance types using the same clustering method.

#### Main figure clustering

Calculate means, store as separate `dfmean` table.

```
nonrepvars = c("nreads", "Effect_Size", "Distance", "Normalization")
edfmean = ddply(subset(edf, AccuracyMeasure == "fracCorrect"), .variables = nonrepvars, 
    .fun = function(x, vars) {
        data.frame(x[1, vars, drop = FALSE], Accuracy = mean(x$Accuracy), sd.Acc = sd(x$Accuracy), 
            stringsAsFactors = FALSE)
    }, nonrepvars)
# Subset to just the panels you want to show in the figure.
edfsmean = subset(edfmean, !Normalization %in% c("edgeRTMM", "edgeRRLE") & !Distance %in% 
    c("PoissonDist, type='mle'", "PoissonDist, type='deseq'") & !nreads %in% 
    c(5000))
edfsmean$Distance[edfsmean$Distance == "PoissonDist, type='quantile'"] <- "PoissonDist"
```

---

## Figure 3

Further refine data in `edfsmean` for better plotting.

```
# Rename the library size to include plotmath symbol for clarity
lib_levels = paste0("tilde(N)[L]==", sort(unique(as.integer(edfsmean$nreads))))
edfsmean$nreads <- factor(paste0("tilde(N)[L]==", edfsmean$nreads), levels = lib_levels)
```

```
pathsize = 0.5
pointsize = 1.5
errorwidth = 0.1
erroralpha = 0.35
errorsize = 0.4
normlegtitle = "Normalization Method:"
pclust0 = ggplot(edfsmean, aes(Effect_Size, Accuracy, color = Normalization)) + 
    geom_errorbar(aes(ymax = Accuracy + sd.Acc, ymin = Accuracy - sd.Acc), width = errorwidth, 
        alpha = erroralpha, size = errorsize, position = "dodge") + geom_path(size = pathsize) + 
    geom_point(aes(shape = Normalization), size = pointsize) + facet_grid(nreads ~ 
    Distance, labeller = label_parsed) + scale_colour_discrete(guide = guide_legend(title = normlegtitle)) + 
    scale_shape_discrete(guide = guide_legend(title = normlegtitle)) + scale_y_continuous(breaks = c(0.6, 
    0.8, 1), limits = c(0.5, 1), oob = function(x, limits) {
    x
}) + scale_x_continuous(breaks = c(1.15, 2, 3), minor_breaks = c(1.5, 2.5, 3.5)) + 
    xlab("Effect Size") + theme(plot.margin = unit(c(0, 0, 0, 0), "cm")) + theme(text = element_text(size = 8), 
    plot.title = element_text(size = 10), strip.text = element_text(size = 8)) + 
    theme(legend.position = "top", legend.box = "horizontal", legend.margin = unit(-0.5, 
        "cm")) + theme(strip.background = element_blank())  # + ggtitle('Clustering Accuracy') 
print(pclust0)
```

Save to file

```
ht = 4
wi = 6.83
dev.off()
```

```
## null device 
##           1
```

```
ggsave("Figure_3.pdf", pclust0, width = wi, height = ht)
```

Add back the subsets for supplemental doc.

```
pclust0 %+% edfmean
```

---

# Save prior to additional rarefying analysis

```
save.image(paste0(savedatafile, "-", datestamp, ".RData"))
# Call garbage collection, clear out any available RAM
gc()
```

```
##             used   (Mb) gc trigger (Mb)  max used (Mb)
## Ncells   3358805  179.4    5241317  280   5241317  280
## Vcells 137055634 1045.7  340485753 2598 340484987 2598
```

---

# The effect of minimum-library size threshold on clustering accuracy

For each “rarefied” simulation above, we chose the minimum library size to retain in the simulation based on an arbitrary quantile of the total library sizes in each simulated experiment. In practice, this choice is made subjectively based on the distribution of library sizes and other considerations that typically vary among researchers. The robustness of this choice very likely depends on the microbiomes being investigated and the way the data is being used in analysis downstream. Here we will evaluate the performance (clustering accuracy) as a function of this choice, library-size-minimum, over a range of expected library sizes and classification difficulties (effect size, aritifical mixing parameter, respectively).

```
rareparams = apply(expand.grid(simparams, rarefy_fracs, rarereps), 1, paste0, 
    collapse = comdelim)
# rarefying in parallel.
rarelist <- foreach(parami = rareparams, .packages = "phyloseq") %dopar% {
    # parami=rareparams[39]
    smalltrim = as.numeric(strsplit(parami, split = comdelim)[[1]][4])
    physeqname = paste(strsplit(parami, split = comdelim)[[1]][1:3], collapse = comdelim)
    physeq = simlist0[[physeqname]]
    physeq = randomsubsample(physeq, smalltrim, replace = FALSE)
    # Make sure to `simpletrim` the new subsampled data before use.
    physeq = simpletrim(physeq, J)
    return(physeq)
}
names(rarelist) <- rareparams
```

### Re-perform evaluations on “rarefied” counts

```
# UniFrac dist methods
raredistcoms = c(paste(names(rarelist), "distance", "'unifrac'", sep = comdelim), 
    paste(names(rarelist), "distance", "'unifrac'", "weighted=TRUE", sep = comdelim), 
    paste(names(rarelist), "distance", "'bray'", sep = comdelim))
# edgeR raredistcoms
edgeRmethtypes = c("method='bcv'")
edgeRgenetypes = c("gene.selection='pairwise'")
edgeRtopvalues = c("top=2000")
edgeRmatcoms = expand.grid(names(rarelist), "edgeRdist", edgeRmethtypes, edgeRgenetypes, 
    edgeRtopvalues)
edgedistcoms = apply(edgeRmatcoms, 1, paste0, collapse = comdelim)
raredistcoms = c(raredistcoms, edgedistcoms)
# DESeq. raredistcoms
DESeqdisttypes = c("method='euclidean'", "method='unifrac', weighted=TRUE")
DESeqmatcoms = expand.grid(names(rarelist), "deseqdistwrap", DESeqdisttypes)
DESeqdistcoms = apply(DESeqmatcoms, 1, paste0, collapse = comdelim)
raredistcoms = c(raredistcoms, DESeqdistcoms)
# PoiClaClu 'normalized'... built-in to the model used in the distance
# function:
raredistcoms = c(raredistcoms, paste(names(rarelist), "PoissonDist", "type='deseq'", 
    sep = comdelim))
```

## Calculate distances

Calculate distances using the vector of `"_"`-delimited commands above.

```
require("foreach")
require("doParallel")
# If need to re-register cluster. registerDoParallel(cl) Calculate distances
# on rarefied counts.
rlist <- foreach(dcom = raredistcoms, .packages = c("phyloseq", "PoiClaClu", 
    "edgeR", "DESeq"), .export = c("edgeRnorm", "edgeRdist", "PoissonDist", 
    "deseq_varstab", "deseqdistwrap")) %dopar% {

    cmdsplit = strsplit(dcom, split = comdelim, fixed = TRUE)[[1]]
    physeq = rarelist[[paste0(cmdsplit[1:5], collapse = comdelim)]]
    if (length(cmdsplit) == 6) {
        dd = do.call(cmdsplit[6], physeq)
    } else if (length(cmdsplit) > 6) {
        fcall = paste0(cmdsplit[6], "(physeq, ", paste(cmdsplit[7:length(cmdsplit)], 
            collapse = ", "), ")")
        dd = eval(parse(text = fcall))
    } else {
        stop("split distance command string is unexpected length")
    }
    # Check that dd has Labels, or it will screw up clustering eval downstream
    if (is.null(attributes(dd)$Labels)) {
        attributes(dd)$Labels <- sample_names(physeq)
    }
    return(dd)
}
names(rlist) <- raredistcoms
all(sapply(rlist, class) == "dist")
```

```
## [1] TRUE
```

```
# Fix bad (NA, infinite) values in rlist
rlist = fixbadDs(rlist)
```

Binary clustering using `limma::pam` (partitioning around medoids) with `k==2`, and alternatively `stats::kmeans` and `stats::hclust`.

```
# pam
RClist <- foreach(dd = rlist, .packages = "cluster") %dopar% {
    pam(dd, k = 2, cluster.only = TRUE)
}
names(RClist) <- names(rlist)
# kmeans
RClist_kmeans <- foreach(dd = rlist, .packages = "stats") %dopar% {
    kmeans(cmdscale(dd, 2), 2)$cluster
}
names(RClist_kmeans) <- names(rlist)
# hclust
RClist_hclust <- foreach(dd = rlist, .packages = "stats") %dopar% {
    cutree(hclust(dd), k = 2)
}
names(RClist_hclust) <- names(rlist)
```

## Evaluate

PAM is the default clustering algorithm for this simulation. We'll tally its results as its results as `rdf`, and we'll tally the others in the next section with name-qualifiers, just like for the `RClist` above.

```
# pam
rdf = ldply(RClist, function(x, binaryclusteval, tot) {
    clres = binaryclusteval(x, tot)
    return(data.frame(Accuracy = clres["fracCorrect"]))
}, binaryclusteval, tot = (2 * J))
colnames(rdf)[1] <- "fcall"
# Define the expected number of reads included.
rdf$nreads <- as(sapply(strsplit(rdf$fcall, comdelim, fixed = TRUE), function(x) x[1]), 
    "integer")
# The replicate number
rdf$replicate <- as(sapply(strsplit(rdf$fcall, comdelim, fixed = TRUE), function(x) x[2]), 
    "numeric")
# The mix factor
rdf$mix_factor <- as(sapply(strsplit(rdf$fcall, comdelim, fixed = TRUE), function(x) x[3]), 
    "numeric")
# The library minimum factor
rdf$library_min <- as(sapply(strsplit(rdf$fcall, comdelim, fixed = TRUE), function(x) x[4]), 
    "numeric")
# use the remaining info to define the function call
rdf$Distance <- sapply(strsplit(rdf$fcall, comdelim, fixed = TRUE), function(x) {
    paste(x[-(1:5)][1:3], collapse = ", ")
})
```

---

## Figure 4

#### Main figure rarefy min

Calculate means, store as separate `rdfmean` table.

```
nonrepvars = c("nreads", "mix_factor", "Distance", "library_min")
rdfmean = ddply(rdf, .variables = nonrepvars, .fun = function(x, vars) {
    data.frame(x[1, vars, drop = FALSE], Accuracy = mean(x$Accuracy), sd.Acc = sd(x$Accuracy), 
        stringsAsFactors = FALSE)
}, nonrepvars)
rdfmean2 = rdfmean
# Re-label the effect-size parameter (mix_factor) to include 'ES='
rdfmean$effect_size <- paste0("ES==", rdfmean$mix_factor)
rdfmean2$effect_size <- paste0("ES==", rdfmean2$mix_factor)
# Rename the library size to include plotmath symbol for clarity
lib_levels = paste0("tilde(N)[L]==", sort(unique(as.integer(rdfmean2$nreads))))
rdfmean2$nreads <- factor(paste0("tilde(N)[L]==", rdfmean2$nreads), levels = lib_levels)
# Remove the variance stabilized versions
rdfmean2 = rdfmean2[grep("deseqdistwrap, method='unifrac', weighted=TRUE", rdfmean2$Distance, 
    fixed = TRUE, invert = TRUE), ]
rdfmean2 = rdfmean2[grep("deseqdistwrap, method='euclidean'", rdfmean2$Distance, 
    fixed = TRUE, invert = TRUE), ]
# Relabel Distance measure names
rdfmean2$Distance <- gsub("distance, 'unifrac', weighted=TRUE", "UniFrac-w", 
    rdfmean2$Distance, fixed = TRUE)
rdfmean2$Distance[grep("distance, 'unifrac'", rdfmean2$Distance)] <- "UniFrac-u"
rdfmean2$Distance[grep("edgeRdist", rdfmean2$Distance)] <- "top-MSD"
rdfmean2$Distance[grep("bray", rdfmean2$Distance)] <- "Bray-Curtis"
rdfmean2$Distance[grep("PoissonDist", rdfmean2$Distance)] <- "PoissonDist"
# rdfmean2$Distance[grep('deseqdistwrap, method='unifrac', weighted=TRUE',
# rdfmean2$Distance, fixed=TRUE)] <- 'DESeq-VS-UniFrac-w'
# rdfmean2$Distance[grep('deseqdistwrap, method='euclidean'',
# rdfmean2$Distance, fixed=TRUE)] <- 'DESeq-VS-Euclidean'
```

Define Figure 4 graphic.

```
pathsize = 0.5
pointsize = 1.5
errorwidth = 0.01
erroralpha = 0.35
errorsize = 0.45
distlegtitle = "Distance Method:"
# The ggplot2 plot definition.
pclustr = ggplot(rdfmean2, aes(library_min, Accuracy, color = Distance)) + geom_errorbar(aes(ymax = Accuracy + 
    sd.Acc, ymin = Accuracy - sd.Acc), width = errorwidth, alpha = erroralpha, 
    size = errorsize, position = "dodge") + geom_path(size = pathsize) + geom_point(aes(shape = Distance), 
    size = pointsize) + facet_grid(nreads ~ effect_size, labeller = label_parsed) + 
    scale_colour_discrete(guide = guide_legend(title = distlegtitle)) + scale_shape_discrete(guide = guide_legend(title = distlegtitle)) + 
    scale_y_continuous(breaks = c(0.6, 0.8, 1), limits = c(-5, 1.1), oob = function(x, 
        limits) {
        x
    }) + xlab("Library Size Minimum Quantile") + coord_cartesian(ylim = c(0.5, 
    1)) + theme(plot.margin = unit(c(0, 0, 0, 0), "cm")) + theme(text = element_text(size = 8), 
    plot.title = element_text(size = 10), strip.text = element_text(size = 8)) + 
    theme(legend.position = "top", legend.box = "horizontal", legend.margin = unit(-0.5, 
        "cm")) + theme(strip.background = element_blank())  #+ ggtitle('Clustering Accuracy, rarefied counts only') 
print(pclustr)
```

```
# Subset to just the panels you want to show in the main figure.
pclustr2 = pclustr
pclustr2$data <- subset(pclustr2$data, mix_factor %in% c(1.15, 1.25, 1.5))  #, 1.75, 2.00, 2.50, 3.50))
# Subset to just the distances you want to show in the main figure.
pclustr2$data <- pclustr2$data[grep("DESeq-VS", pclustr2$data$Distance, fixed = TRUE, 
    invert = TRUE), ]
ht = 4
wi = 6.83
dev.off()
```

```
## null device 
##           1
```

```
ggsave("Figure_4.pdf", pclustr2, width = wi, height = ht)
```

Add back the subsets for supplemental documentation.

```
pclustr2 %+% rdfmean
```

###### Shows u-UniFrac eventually converge at larger effect-sizes. Shows that there wasn't some artificial ceiling built into the template datasets that precluded u-UniFrac from discriminating them. It just didn't do a good job until they were very clearly separated (many zeros) between the two sample types. Somewhere around a mixing parameter of `2.5` in this simulation results:

---

## Alternatives to partitioning around medoids for clustering (supplemental only)

To save development time, this contains a lot of code repeated from above, but with `_hclust` or `_kmeans` appended to variable names.

### Hierarchical clustering (hclust)

```
rdf_hclust = ldply(RClist_hclust, function(x, binaryclusteval, tot) {
    clres = binaryclusteval(x, tot)
    return(data.frame(Accuracy = clres["fracCorrect"]))
}, binaryclusteval, tot = (2 * J))
colnames(rdf_hclust)[1] <- "fcall"
# Define the expected number of reads included.
rdf_hclust$nreads <- as(sapply(strsplit(rdf_hclust$fcall, comdelim, fixed = TRUE), 
    function(x) x[1]), "integer")
# The replicate number
rdf_hclust$replicate <- as(sapply(strsplit(rdf_hclust$fcall, comdelim, fixed = TRUE), 
    function(x) x[2]), "numeric")
# The mix factor
rdf_hclust$mix_factor <- as(sapply(strsplit(rdf_hclust$fcall, comdelim, fixed = TRUE), 
    function(x) x[3]), "numeric")
# The library minimum factor
rdf_hclust$library_min <- as(sapply(strsplit(rdf_hclust$fcall, comdelim, fixed = TRUE), 
    function(x) x[4]), "numeric")
# use the remaining info to define the function call
rdf_hclust$Distance <- sapply(strsplit(rdf_hclust$fcall, comdelim, fixed = TRUE), 
    function(x) {
        paste(x[-(1:5)][1:3], collapse = ", ")
    })
# Calculate means, store as separate `rdf_hclustmean` table.
nonrepvars = c("nreads", "mix_factor", "Distance", "library_min")
rdf_hclustmean = ddply(rdf_hclust, .variables = nonrepvars, .fun = function(x, 
    vars) {
    data.frame(x[1, vars, drop = FALSE], Accuracy = mean(x$Accuracy), sd.Acc = sd(x$Accuracy), 
        stringsAsFactors = FALSE)
}, nonrepvars)
rdf_hclustmean2 = rdf_hclustmean
# Rename the library size to include plotmath symbol for clarity
lib_levels = paste0("tilde(N)[L]==", sort(unique(as.integer(rdf_hclustmean2$nreads))))
rdf_hclustmean2$nreads <- factor(paste0("tilde(N)[L]==", rdf_hclustmean2$nreads), 
    levels = lib_levels)
# Re-label the effect-size parameter (mix_factor) to include 'ES='
rdf_hclustmean2$effect_size <- paste0("ES==", rdf_hclustmean2$mix_factor)
# Remove the variance stabilized versions
rdf_hclustmean2 = rdf_hclustmean2[grep("deseqdistwrap, method='unifrac', weighted=TRUE", 
    rdf_hclustmean2$Distance, fixed = TRUE, invert = TRUE), ]
rdf_hclustmean2 = rdf_hclustmean2[grep("deseqdistwrap, method='euclidean'", 
    rdf_hclustmean2$Distance, fixed = TRUE, invert = TRUE), ]
# Relabel Distance measure names
rdf_hclustmean2$Distance <- gsub("distance, 'unifrac', weighted=TRUE", "UniFrac-w", 
    rdf_hclustmean2$Distance, fixed = TRUE)
rdf_hclustmean2$Distance[grep("distance, 'unifrac'", rdf_hclustmean2$Distance)] <- "UniFrac-u"
rdf_hclustmean2$Distance[grep("edgeRdist", rdf_hclustmean2$Distance)] <- "top-MSD"
rdf_hclustmean2$Distance[grep("bray", rdf_hclustmean2$Distance)] <- "Bray-Curtis"
rdf_hclustmean2$Distance[grep("PoissonDist", rdf_hclustmean2$Distance)] <- "PoissonDist"
```

Define Figure 4 (hclust) graphic.

```
pathsize = 0.5
pointsize = 1.5
errorwidth = 0.01
erroralpha = 0.35
errorsize = 0.45
distlegtitle = "Distance Method:"
# The ggplot2 plot definition.
pclustr_hclust = ggplot(rdf_hclustmean2, aes(library_min, Accuracy, color = Distance)) + 
    geom_errorbar(aes(ymax = Accuracy + sd.Acc, ymin = Accuracy - sd.Acc), width = errorwidth, 
        alpha = erroralpha, size = errorsize, position = "dodge") + geom_path(size = pathsize) + 
    geom_point(aes(shape = Distance), size = pointsize) + facet_grid(nreads ~ 
    effect_size, labeller = label_parsed) + scale_colour_discrete(guide = guide_legend(title = distlegtitle)) + 
    scale_shape_discrete(guide = guide_legend(title = distlegtitle)) + scale_y_continuous(breaks = c(0.6, 
    0.8, 1), limits = c(-5, 1.1), oob = function(x, limits) {
    x
}) + xlab("Library Size Minimum Quantile") + coord_cartesian(ylim = c(0.5, 1)) + 
    theme(text = element_text(size = 8), plot.title = element_text(size = 10), 
        strip.text = element_text(size = 8)) + theme(legend.position = "top", 
    legend.box = "horizontal", legend.margin = unit(-0.5, "cm")) + theme(strip.background = element_blank())  #+ ggtitle('Clustering Accuracy, rarefied counts only') 
print(pclustr_hclust)
```

```
# Subset to just the panels you want to show in the main figure.
pclustr_hclust2 = pclustr_hclust
pclustr_hclust2$data <- subset(pclustr_hclust2$data, mix_factor %in% c(1.15, 
    1.25, 1.5, 1.75, 2, 2.5, 3.5))
# Subset to just the distances you want to show in the main figure.
pclustr_hclust2$data <- pclustr_hclust2$data[grep("DESeq-VS", pclustr_hclust2$data$Distance, 
    fixed = TRUE, invert = TRUE), ]
ht = 4
wi = 6.83
dev.off()
```

```
## null device 
##           1
```

```
ggsave("Figure_4_hclust.pdf", pclustr_hclust2, width = wi, height = ht)
```

### k-means clustering (kmeans)

```
rdf_kmeans = ldply(RClist_kmeans, function(x, binaryclusteval, tot) {
    clres = binaryclusteval(x, tot)
    return(data.frame(Accuracy = clres["fracCorrect"]))
}, binaryclusteval, tot = (2 * J))
colnames(rdf_kmeans)[1] <- "fcall"
# Define the expected number of reads included.
rdf_kmeans$nreads <- as(sapply(strsplit(rdf_kmeans$fcall, comdelim, fixed = TRUE), 
    function(x) x[1]), "integer")
# The replicate number
rdf_kmeans$replicate <- as(sapply(strsplit(rdf_kmeans$fcall, comdelim, fixed = TRUE), 
    function(x) x[2]), "numeric")
# The mix factor
rdf_kmeans$mix_factor <- as(sapply(strsplit(rdf_kmeans$fcall, comdelim, fixed = TRUE), 
    function(x) x[3]), "numeric")
# The library minimum factor
rdf_kmeans$library_min <- as(sapply(strsplit(rdf_kmeans$fcall, comdelim, fixed = TRUE), 
    function(x) x[4]), "numeric")
# use the remaining info to define the function call
rdf_kmeans$Distance <- sapply(strsplit(rdf_kmeans$fcall, comdelim, fixed = TRUE), 
    function(x) {
        paste(x[-(1:5)][1:3], collapse = ", ")
    })
# Calculate means, store as separate `rdf_kmeansmean` table.
nonrepvars = c("nreads", "mix_factor", "Distance", "library_min")
rdf_kmeansmean = ddply(rdf_kmeans, .variables = nonrepvars, .fun = function(x, 
    vars) {
    data.frame(x[1, vars, drop = FALSE], Accuracy = mean(x$Accuracy), sd.Acc = sd(x$Accuracy), 
        stringsAsFactors = FALSE)
}, nonrepvars)
rdf_kmeansmean2 = rdf_kmeansmean
# Rename the library size to include plotmath symbol for clarity
lib_levels = paste0("tilde(N)[L]==", sort(unique(as.integer(rdf_kmeansmean2$nreads))))
rdf_kmeansmean2$nreads <- factor(paste0("tilde(N)[L]==", rdf_kmeansmean2$nreads), 
    levels = lib_levels)
# Re-label the effect-size parameter (mix_factor) to include 'ES='
rdf_kmeansmean2$effect_size <- paste0("ES==", rdf_kmeansmean2$mix_factor)
# Remove the variance stabilized versions
rdf_kmeansmean2 = rdf_kmeansmean2[grep("deseqdistwrap, method='unifrac', weighted=TRUE", 
    rdf_kmeansmean2$Distance, fixed = TRUE, invert = TRUE), ]
rdf_kmeansmean2 = rdf_kmeansmean2[grep("deseqdistwrap, method='euclidean'", 
    rdf_kmeansmean2$Distance, fixed = TRUE, invert = TRUE), ]
# Relabel Distance measure names
rdf_kmeansmean2$Distance <- gsub("distance, 'unifrac', weighted=TRUE", "UniFrac-w", 
    rdf_kmeansmean2$Distance, fixed = TRUE)
rdf_kmeansmean2$Distance[grep("distance, 'unifrac'", rdf_kmeansmean2$Distance)] <- "UniFrac-u"
rdf_kmeansmean2$Distance[grep("edgeRdist", rdf_kmeansmean2$Distance)] <- "top-MSD"
rdf_kmeansmean2$Distance[grep("bray", rdf_kmeansmean2$Distance)] <- "Bray-Curtis"
rdf_kmeansmean2$Distance[grep("PoissonDist", rdf_kmeansmean2$Distance)] <- "PoissonDist"
```

Define Figure 4 (kmeans) graphic.

```
pathsize = 0.5
pointsize = 1.5
errorwidth = 0.01
erroralpha = 0.35
errorsize = 0.45
distlegtitle = "Distance Method:"
# The ggplot2 plot definition.
pclustr_kmeans = ggplot(rdf_kmeansmean2, aes(library_min, Accuracy, color = Distance)) + 
    geom_errorbar(aes(ymax = Accuracy + sd.Acc, ymin = Accuracy - sd.Acc), width = errorwidth, 
        alpha = erroralpha, size = errorsize, position = "dodge") + geom_path(size = pathsize) + 
    geom_point(aes(shape = Distance), size = pointsize) + facet_grid(nreads ~ 
    effect_size, labeller = label_parsed) + scale_colour_discrete(guide = guide_legend(title = distlegtitle)) + 
    scale_shape_discrete(guide = guide_legend(title = distlegtitle)) + scale_y_continuous(breaks = c(0.6, 
    0.8, 1), limits = c(-5, 1.1), oob = function(x, limits) {
    x
}) + xlab("Library Size Minimum Quantile") + coord_cartesian(ylim = c(0.5, 1)) + 
    theme(text = element_text(size = 8), plot.title = element_text(size = 10), 
        strip.text = element_text(size = 8)) + theme(legend.position = "top", 
    legend.box = "horizontal", legend.margin = unit(-0.5, "cm")) + theme(strip.background = element_blank())  #+ ggtitle('Clustering Accuracy, rarefied counts only') 
print(pclustr_kmeans)
```

```
# Subset to just the panels you want to show in the main figure.
pclustr_kmeans2 = pclustr_kmeans
pclustr_kmeans2$data <- subset(pclustr_kmeans2$data, mix_factor %in% c(1.15, 
    1.25, 1.5, 1.75, 2, 2.5, 3.5))
# Subset to just the distances you want to show in the main figure.
pclustr_kmeans2$data <- pclustr_kmeans2$data[grep("DESeq-VS", pclustr_kmeans2$data$Distance, 
    fixed = TRUE, invert = TRUE), ]
ht = 4
wi = 6.83
dev.off()
```

```
## null device 
##           1
```

```
ggsave("Figure_4_kmeans.pdf", pclustr_kmeans2, width = wi, height = ht)
```

---

# Final Save

```
# Stop the cluster (parallel)
stopCluster(cl)
# Stop the cluster (Rmpi/doMPI) closeCluster(cl) mpi.finalize() Save
save.image(paste0(savedatafile, "-", datestamp, ".RData"))
```
