# Supplementary material for: Waste Not, Want Not: Why Rarefying Microbiome Data Is Inadmissible
Source: PLoS Comput Biol. 2014 Apr 3;10(4):e1003531. doi: 10.1371/journal.pcbi.1003531 (PMC3974642; doi:10.1371/journal.pcbi.1003531)
Supplement: Protocol S1 — A zip file containing all supplementary source files. This includes the Rmd source code, HTML output, and all related documentation and code to completely and exactly recreate every results figure in this article. (ZIP) [file pcbi.1003531.s001.zip › norarefy-source/simulation-differential-abundance/simulation-differential-abundance-server.html]

Simulation for Differential Abundance Detection Performance


# Simulation for Differential Abundance Detection Performance

---

## Load/Install requisite packages

```
# The required package list:
reqpkg = c("cluster", "doParallel", "edgeR", "DESeq", "DESeq2", "foreach", "ggplot2", 
    "grid", "scales", "metagenomeSeq", "phyloseq", "plyr", "reshape2", "ROCR")
# Load all required packages and show version
for (i in reqpkg) {
    print(i)
    print(packageVersion(i))
    library(i, quietly = TRUE, verbose = FALSE, warn.conflicts = FALSE, character.only = TRUE)
}
```

```
## [1] "cluster"
## [1] '1.14.4'
## [1] "doParallel"
## [1] '1.0.6'
## [1] "edgeR"
## [1] '3.4.0'
## [1] "DESeq"
## [1] '1.14.0'
## [1] "DESeq2"
## [1] '1.2.6'
## [1] "foreach"
## [1] '1.4.1'
## [1] "ggplot2"
## [1] '0.9.3.1'
## [1] "grid"
## [1] '3.0.2'
## [1] "scales"
## [1] '0.2.3'
## [1] "metagenomeSeq"
## [1] '1.4.0'
## [1] "phyloseq"
## [1] '1.7.10'
## [1] "plyr"
## [1] '1.8'
## [1] "reshape2"
## [1] '1.2.2'
## [1] "ROCR"
## [1] '1.0.5'
```

Load some theme parameters for ggplot2.

```
theme_set(theme_bw())
pal = "Set1"
scale_colour_discrete <- function(palname = pal, ...) {
    scale_colour_brewer(palette = palname, ...)
}
scale_fill_discrete <- function(palname = pal, ...) {
    scale_fill_brewer(palette = palname, ...)
}
```

---

## Set parameters for this simulation

```
set.seed(20131205)
savedatafile = "simulation-differential-abundance"
data("GlobalPatterns")
# load(savedatafile)

# Define the number of true-positives per sample
nTP = 30

# Minimum number of reads to consider an OTU 'observed' in a sample
minobs = 1L

# The delimiter in the command parameter string
comdelim = "_"

# Define the different biological source templates to use sampletypes =
# c('Ocean', 'Soil')
sampletypes = levels(get_variable(GlobalPatterns, "SampleType"))

# Define the ceiling in the number of OTUs to consider in the template
# multinomial nOTUs = 353L
nOTUs = 2000L

# Define the number of samples in each class of a simulated experiment J =
# c(3, 10)
J = c(3, 5, 10)

# The different values of effect size to apply foldeffect = c(1.5, 5, 20)
foldeffect = c(1.25, 2.5, 5, 10, 20)

## The number of reads per sample, ns ns = c(2000, 5E4)
ns = c(2000, 10000, 50000)

# Vector of the replicate numbers to repeat for each comb of simulation
# parameters (n, etc) reps=1:2
reps = 1:3

# The number of cores to use in this simulation Ncores = 7
Ncores = 30

# Define the simulation parameters combinations
simparams = apply(expand.grid(ns, sampletypes, reps, foldeffect, J), 1, paste0, 
    collapse = comdelim)
# Define the labels to go with each element of the simulation parameter
# after splitting on the delimiter
simparamslabels = c("nreads", "SampleType", "Replicate", "EffectSize", "nsamples")

# Define date-stamp for file names
datestamp = gsub(":", "_", gsub("[[:space:]]+", "-", date()), fixed = TRUE)
print(datestamp)
```

```
## [1] "Thu-Dec-5-11_31_17-2013"
```

## Parameter definitions

The major factors contributing to the computation cost in this simulation example are the number of OTUs retained from the template `GlobalPatterns` dataset, which ultimately is used to dictate the length of the multinomials and their corresponding proportion vectors specified by \(\pi\) (R variable `pi` or `pis`); and the number of samples being simulated.

- `nOTUs` – the number of most-prevalent OTUs to keep in simulation template, 2000.
- `minobs` – The minimum abundance value, 1, for which an OTU is “counted” as having been observed in a given sample. This is used for ranking OTUs according to the number of samples in which they appeared.
- `J` – The number of samples per simulated table. For multitable analysis this needs to be consistent across tables, so included here in the beginning as a global parameter. Value for this simulation is 3, 5, 10.
- `foldeffect` – The fold-weighting of the values in the “test” samples relative to the control samples. Current value is 1.25, 2.5, 5, 10, 20.
- `ns` – A vector of the expected values for the sampling depth that are nevertheless subject to the random sampling from the original template totals. The read numbers should not exceed the total number of reads in the template, so this is checked in the “simulation” (subsampling) module, and a ceiling used. The values for this vector of sampling depth means in this particular simulation is: 2000, 104, 5 × 10^4^.

---

# Define template

Trim to just those samples that are used in this analysis.

```
data("GlobalPatterns")
sampsums = sample_sums(GlobalPatterns)
keepsamples = sample_data(GlobalPatterns)$SampleType %in% sampletypes
```

Trim OTUs that do not appear in very many samples in the template. Sort by prevalance (number of samples appeared) and then by abundance value.

```
template = prune_samples(keepsamples, GlobalPatterns)
# Make a list of source templates
templatelist = lapply(sampletypes, function(i, tempall, minobs, nOTUs) {
    cat(i, "\n")
    whtemp = (get_variable(tempall, "SampleType") %in% i)
    templatei = prune_samples(whtemp, tempall)
    samobs = apply(otu_table(templatei), 1, function(x, m) sum(x > m), m = minobs)
    otudf = data.frame(prev = samobs, sums = taxa_sums(templatei))
    otudf = otudf[order(-otudf$prev, -otudf$sums), ]
    # Trim all but the first nOTUs
    return(prune_taxa(rownames(otudf)[1:nOTUs], templatei))
}, template, minobs, nOTUs)
```

```
## Feces 
## Freshwater 
## Freshwater (creek) 
## Mock 
## Ocean 
## Sediment (estuary) 
## Skin 
## Soil 
## Tongue
```

```
names(templatelist) <- sampletypes
```

---

# Simulate microbiome data from templates

## Simulation Function

- Input
  - `postfix`, `template`, `J`, `n` (number or reads)
- Output
  - phyloseq object, incorporating simulation results and inputs

```
microbesim = function(postfix = "sim", template, J, n = 10000) {
    # Generate `J` simulated microbiomes with `n` total reads each (all the
    # same, or n has length equal to the value of `J`), with subsamples drawn
    # from `template`.  `postfix` is a dummy idenitifer added to help
    # distinguish simulated samples in downstream code.
    require("phyloseq")
    # call the proporitions vector `pi`, similar to nomenclature from DMN
    pi = taxa_sums(template)
    # n must be a scalar (recycled as the number of reads for every simulation)
    # or it can be vector of length equal to J, the number of samples being
    # simulated.
    if (length(J) != 1) {
        stop("Length of J should be 1.")
    }
    if (length(n) != 1 & length(n) != J) {
        stop("n should be length 1, or length J.")
    }
    # Actually create the simulated abundance table
    simat = mapply(function(i, x, sample.size) {
        if (FALSE) 
            {
                print(i)
            }  # i is a dummy iterator
        phyloseq:::rarefaction_subsample(x, sample.size)
    }, i = 1:J, sample.size = n, MoreArgs = list(x = pi), SIMPLIFY = TRUE)
    simat = t(simat)
    # Add the OTU names to the OTU (column) indices
    colnames(simat) <- names(pi)
    # Add new simulated sample_names to the row (sample) indices
    rownames(simat) <- paste(i, "::", 1:nrow(simat), postfix, sep = "")
    # Put simulated abundances together with metadata as a phyloseq object
    OTU = otu_table(simat, taxa_are_rows = FALSE)
    # Define data.frame that will become sample_data
    SDF = data.frame(sample = sample_names(OTU), TableNumber = i, type = "simulated")
    SDF$postfix <- postfix
    rownames(SDF) <- sample_names(OTU)
    SD = sample_data(SDF)
    # Return a phyloseq object
    return(phyloseq(OTU, SD))
}
```

Define a function for sampling from the library sizes as a way of including the “noise” derived from this aspect of the data as well.

```
# rescale the sum of reads in the original raw(-ish) template data to the
# expected library size being requested here
sumsim = function(n, sumtemplate, J) {
    # `n` - expected size target `sumtemplate` - the template vector of library
    # sizes observed in template `J` - The number of sample sizes to return
    scaledSums = round(n * (sumtemplate/median(sumtemplate)))
    return(sample(scaledSums, size = J, replace = TRUE))
}
```

## Use new functions to simulate from different templates

Register parallel clusters for parallel calculations (e.g. UniFrac, etc.).

```
# makeCluster makeForkCluster() The parallel-package, usually single-node
# way
cl <- makeCluster(Ncores)
# # The multi-node way, from: #
# http://www.bioconductor.org/help/bioconductor-cloud-ami/#parallel
# library('parallel') lines <- readLines('/usr/local/Rmpi/hostfile.plain') #
# Skip the master node: lines <- lines[-1] # Last one causing problems lines
# <- lines[-15] hosts <- character() for (line in lines){ x <-
# (strsplit(line[[1]], ' ')) hosts <- c(hosts, rep.int(x[[1]][1],
# as.integer(x[[1]][2]))) } cl <- makePSOCKcluster(hosts,
# master=system('hostname -i', intern=TRUE)) system.time(clusterCall(cl,
# Sys.sleep, 1)) # A test
registerDoParallel(cl)
```

Repeat the subsampling simulation many times, with different values for the number of reads per sample.

```
# Parallelized simulations
simlist <- foreach(i = simparams, .packages = c("phyloseq")) %dopar% {
    # i = simparams[1]
    params = strsplit(i, comdelim)[[1]]
    names(params) <- simparamslabels
    # Initialize
    n = sim = sim1 = sim2 = n1 = n2 = NULL
    # cat(i, '\n')
    n = as.numeric(params["nreads"])
    sampletypei = params["SampleType"]
    # The number of samples to use for each class in this simulation
    Ji = as.integer(params["nsamples"])
    templatei = templatelist[[sampletypei]]
    # Rarely a simulation has a weird value and fails.  Catch these with `try`,
    # and repeat the simulation call if error (it will be a new seed)
    tryAgain = TRUE
    infiniteloopcounter = 1
    while (tryAgain & infiniteloopcounter < 5) {
        n1 = sumsim(n, sampsums, Ji)
        n2 = sumsim(n, sampsums, Ji)
        sim1 = microbesim(paste0(sampletypei, ";grp1"), templatei, Ji, n1)
        sim2 = microbesim(paste0(sampletypei, ";grp2"), templatei, Ji, n2)
        if (is.null(sim1) | is.null(sim2) | is.null(n1) | is.null(n2) | inherits(sim1, 
            "try-error") | inherits(sim2, "try-error")) {
            tryAgain = TRUE
            infiniteloopcounter = infiniteloopcounter + 1
        } else {
            tryAgain = FALSE
        }
    }
    if (infiniteloopcounter >= 5) {
        stop("Consistent error found during simulation. Need to investigate cause.")
    }
    # Merge the two simulated datasets together into one phyloseq object and add
    # back tree.
    sim = merge_phyloseq(sim1, sim2)
    sim = merge_phyloseq(sim, tax_table(GlobalPatterns), phy_tree(GlobalPatterns))
    return(sim)
}
names(simlist) <- simparams
```

Define a simple function for minimal trimming, removing all-zero or mostly-zero OTUs.

```
simpletrim = function(physeq, minobs) {
    Ji = nsamples(physeq)
    # Force orientation to be sample-by-OTU
    if (taxa_are_rows(physeq)) {
        physeq <- t(physeq)
    }
    # `prevalence` is the fraction of total samples in which an OTU is observed
    # at least `minobs` times.
    prevalence = apply(as(otu_table(physeq), "matrix"), 2, function(x, minobs) {
        return(sum(x > minobs))
    }, minobs)/(Ji)
    # Will only keep OTUs that appear in more than X% of samples and have total
    # reads greater than half the number of samples.
    keepOTUs = prevalence > 0.05 & taxa_sums(physeq) > (0.5 * Ji)
    return(prune_taxa(keepOTUs, physeq))
}
# Simple prune
simlist0 <- lapply(simlist, simpletrim, minobs)
```

---

## Simulate microbiome samples for power analysis

In the sample clustering/classification analysis we pretended to not know the identity of the template of each simulated sample, and then evaluated our ability to accruately cluster the samples using different normalization and distance measures. In this simulation, we use the source template information as if it were an experimental design variable, with a control and test group. Here we are interested in determining which OTUs are *differentially abundant* between the two sample groups. In pracice the two sample groups would come from different sources according to an experimental design. Here we will simulate all samples from the same template, such that every OTU is a true null for differential abundance. We will then randomly modify a subset of the OTUs with a known effect size (fold change) and evaluate how effective we were at detecting these *True Positives*.

```
# Archive the simlist before adding an effect
simlistnoeffect = simlist0
# Apply the specified effect-size to randomly chosen OTUs
simlist0 <- foreach(i = simparams, .packages = c("phyloseq")) %dopar% {
    # physeq = simlist0[[3]]
    physeq = simlist0[[i]]
    params = strsplit(i, comdelim)[[1]]
    names(params) <- simparamslabels
    effectsize = as.numeric(params["EffectSize"])
    # Randomly sample from the available OTU names in physeq and assign them as
    # TP
    TPOTUs = sample(taxa_names(physeq), nTP, replace = FALSE)
    # Define the samples that will have effect applied
    effectsamples = grep(";grp1", sample_names(physeq), fixed = TRUE)
    # Apply effect (multiply abundances by effectsize scalar)
    otu_table(physeq)[effectsamples, TPOTUs] <- effectsize * otu_table(physeq)[effectsamples, 
        TPOTUs]
    # Rename these new 'true positive' OTUs, with 'TP'
    wh.TP = taxa_names(physeq) %in% TPOTUs
    newname = paste0(taxa_names(physeq)[wh.TP], "-TP")
    colnames(physeq@otu_table)[wh.TP] <- newname
    physeq@phy_tree$tip.label[wh.TP] <- newname
    rownames(physeq@tax_table)[wh.TP] <- newname
    return(physeq)
}
names(simlist0) <- names(simlist)
```

Must normalize data for comparison tests. Rarefied, naiive proportion, etc. For rarified, stick with the min lib size for now. Will want to show the effect of this choice later on, similar to the results from the clustering/classification section.

```
# Create rarified data
rngseeds = sample(20130921, size = length(simlist0), replace = TRUE)
rarelist = foreach(rngseedi = rngseeds, physeq = simlist0, .packages = "phyloseq") %dopar% 
    {
        # physeq = simlist0[[1]] rngseedi = rngseeds[[1]]
        physeq = rarefy_even_depth(physeq, rngseed = rngseedi)
        return(physeq)
    }
names(rarelist) <- names(simlist)
```

Save a version of the simulated dataset transformed to sample proportions.

```
proportion = function(physeq) {
    # Normalize total sequences represented
    normf = function(x, tot = max(sample_sums(physeq))) {
        tot * x/sum(x)
    }
    physeq = transform_sample_counts(physeq, normf)
    return(physeq)
}
proplist = foreach(physeq = simlist0, .packages = "phyloseq") %dopar% {
    return(proportion(physeq))
}
```

---

## Detect Differential Abundance, testing and validation

---

### mt on original, rarefied, and proportional simulated counts.

Detect differential abundance using t-test and BH correction, using the `mt` wrapper in phyloseq, and the `p.adjust` function in base R. Make a list of `res` tables with the same labels as `nbinomTest`.

```
physeq2mt_res = function(physeq, varname = "postfix") {
    resmt = mt(physeq, classlabel = get_variable(physeq, varname))
    tab = resmt[, c("rawp"), drop = FALSE]
    tab = cbind(tab, padj = p.adjust(tab[, 1], method = "BH"))
    tab = cbind(id = row.names(tab), tab)
    return(tab)
}
raremt_reslist = foreach(physeq = rarelist, .packages = "phyloseq") %dopar% 
    {
        physeq2mt_res(physeq)
    }
propmt_reslist = foreach(physeq = proplist, .packages = "phyloseq") %dopar% 
    {
        physeq2mt_res(physeq)
    }
nonorm_reslist = foreach(physeq = simlist0, .packages = "phyloseq") %dopar% 
    {
        physeq2mt_res(physeq)
    }
```

---

### DESeq

Define function to perform the DESeq binom test for two classes.

```
deseq_binom_test = function(physeq) {
    # physeq = simlist0[[6]] Create the vector defining the 'experimental
    # design', the two conditions/source templates using the single-semicolon
    # delimiter
    designfac = factor(gsub("[[:print:]]+\\;", "", sample_names(physeq)))
    # Enforce Orientation
    if (!taxa_are_rows(physeq)) {
        physeq <- t(physeq)
    }
    # Convert to matrix, round up to nearest integer
    x = ceiling(as(otu_table(physeq), "matrix")) + 1
    # Add taxonomy data
    taxADF = as(data.frame(as(tax_table(physeq), "matrix")), "AnnotatedDataFrame")
    # Initalize the count data sets.
    cds = newCountDataSet(x, conditions = designfac, featureData = taxADF)
    # First, estimate size factors, then estimate dispersion.  Size factors
    cds = estimateSizeFactors(cds)  #, locfunc=genefilter::shorth)
    sizeFactors(cds)
    # Now dispersions Variance estimation, passing along additional options
    cds = estimateDispersions(cds, fitType = c("local"))
    res = nbinomTest(cds, levels(designfac)[1], levels(designfac)[2])
    return(res)
}
```

Perform DESeq differential abundance detection on both original and rarefied data.

```
DESeqreslist <- foreach(physeq = simlist0, .packages = c("phyloseq", "DESeq")) %dopar% 
    {
        return(deseq_binom_test(physeq))
    }
rare_DESeqreslist <- foreach(physeq = rarelist, .packages = c("phyloseq", "DESeq")) %dopar% 
    {
        return(deseq_binom_test(physeq))
    }
```

---

### DESeq2

Define function wrapping DESeq2

```
eval_DESeq2 = function(physeq, IndFilt = NULL) {
    require("DESeq2")
    require("phyloseq")
    # physeq = simlist0[[1]] physeq = rarelist[[7]] Enforce orientation. Samples
    # are columns
    if (!taxa_are_rows(physeq)) {
        physeq <- t(physeq)
    }
    # Coerce count data to vanilla matrix of integers
    countData = round(as(otu_table(physeq), "matrix"), digits = 0)
    countData = countData + 1L
    colData = data.frame(sample_data(physeq))
    # Re-order the levels so the NULL set is first.
    colData$postfix <- relevel(colData$postfix, levels(colData$postfix)[2])
    # Create the DESeq data set, dds.
    dds <- DESeqDataSetFromMatrix(countData, colData, design = ~postfix)
    # Run DESeq.
    suppressWarnings(dds <- try(DESeq(dds, quiet = TRUE), silent = TRUE))
    if (inherits(dds, "try-error")) {
        # If the parametric fit failed, try the local.
        suppressWarnings(dds <- try(DESeq(dds, fitType = "local", quiet = TRUE), 
            silent = TRUE))
        if (inherits(dds, "try-error")) {
            # If local fails, try the mean
            suppressWarnings(dds <- try(DESeq(dds, fitType = "mean", quiet = TRUE), 
                silent = TRUE))
        }
        if (inherits(dds, "try-error")) {
            # If still bad, quit with error.
            return(NULL)
        }
    }
    res = results(dds)
    # Independent Filtering
    if (!is.null(IndFilt)) {
        use <- res$baseMean >= IndFilt & !is.na(res$pvalue)
        resFilt <- res[use, ]
        resFilt$padj <- p.adjust(resFilt$pvalue, method = "BH")
        res = as(resFilt, "data.frame")
        res[order(res$padj), ]
    }
    res$id <- rownames(res)
    return(res)
}
```

Perform DESeq2 differential abundance detection on both original and rarefied data.

```
DESeq2reslist <- foreach(physeq = simlist0, .packages = c("phyloseq", "DESeq2")) %dopar% 
    {
        return(eval_DESeq2(physeq))
    }
rare_DESeq2reslist <- foreach(physeq = rarelist, .packages = c("phyloseq", "DESeq2")) %dopar% 
    {
        return(eval_DESeq2(physeq))
    }
names(rare_DESeq2reslist) <- names(rarelist)
rare_DESeq2reslist <- rare_DESeq2reslist[-which(sapply(rare_DESeq2reslist, is.null))]
```

---

### edgeR

Define function to normalize in the typical edgeR way.

```
edgeRnorm = function(physeq) {
    require("edgeR")
    require("phyloseq")
    # physeq = simlist0[[1]] physeq = simlist0[[117]] Enforce orientation.
    if (!taxa_are_rows(physeq)) {
        physeq <- t(physeq)
    }
    x = as(otu_table(physeq), "matrix")
    # Add one to protect against overflow, log(0) issues.
    x = x + 1
    # Now turn into a DGEList
    y = DGEList(counts = x, group = get_variable(physeq, "postfix"))  #, remove.zeros=TRUE)
    # Perform edgeR-encoded normalization, using the following method
    z = calcNormFactors(y, method = "RLE")
    # z = calcNormFactors(y, method='TMM') A check that we didn't divide by zero
    # inside `calcNormFactors`
    if (!all(is.finite(z$samples$norm.factors))) {
        stop("Something wrong with edgeR::calcNormFactors on this data, non-finite $norm.factors")
    }
    # Estimate dispersions
    z1 = estimateCommonDisp(z)
    z2 = estimateTagwiseDisp(z1)
    return(z2)
}
```

Now edgeR norm with group factor to each simulation in `simlist0`.

```
edgeRlist <- foreach(physeq = simlist0, .packages = c("phyloseq", "edgeR")) %dopar% 
    {
        return(edgeRnorm(physeq))
    }
rareedgeRlist <- foreach(physeq = rarelist, .packages = c("phyloseq", "edgeR")) %dopar% 
    {
        return(edgeRnorm(physeq))
    }
```

Define function to perform the basic edgeR test for two classes.

```
edgeRtest = function(DGEi) {
    et = exactTest(DGEi)
    tt = topTags(et, n = nrow(DGEi$table), adjust.method = "BH", sort.by = "PValue")
    res = tt@.Data[[1]]
    # Match the others, copy FDR as 'padj'.
    res$padj <- res$FDR
    # Add 'id' column
    res$id <- rownames(res)
    return(res)
}
edgeRreslist = foreach(DGEi = edgeRlist, .packages = "edgeR") %dopar% {
    return(edgeRtest(DGEi))
}
# The rarefied counts results list for edgeR
rare_edgeRreslist = foreach(DGEi = rareedgeRlist, .packages = "edgeR") %dopar% 
    {
        return(edgeRtest(DGEi))
    }
```

### metagenomeSeq

metagenomeSeq is an official Bioconductor package, with the explicit goal of detecting differentail abundance in microbiome experiments with an explicit design. Hence, its relative performance is also important to evaluate.

First, define a function to convert the simulated data into a microbiomeSeq object, and a second function to perform the zero-inflated Gaussian fit based on our simple two-class experimental design used by the other methods as well.

```
# Function to convert form phyloseq object to metagenomeSeq object
make_metagenomeSeq = function(physeq) {
    require("metagenomeSeq")
    require("phyloseq")
    # Enforce orientation
    if (!taxa_are_rows(physeq)) {
        physeq <- t(physeq)
    }
    OTU = as(otu_table(physeq), "matrix")
    # Convert sample_data to AnnotatedDataFrame
    ADF = AnnotatedDataFrame(data.frame(sample_data(physeq)))
    # define dummy 'feature' data for OTUs, using their name Helps with
    # extraction and relating to taxonomy later on.
    TDF = AnnotatedDataFrame(data.frame(OTUname = taxa_names(physeq), row.names = taxa_names(physeq)))
    # Create the metagenomeSeq object
    MGS = newMRexperiment(counts = OTU, phenoData = ADF, featureData = TDF)
    # Trigger metagenomeSeq to calculate its Cumulative Sum scaling factor.
    MGS = cumNorm(MGS)
    return(MGS)
}
# Function to perform relevant test (`fitZig`)
test_metagenomeSeq = function(MGS, variable, physeq = NULL) {
    require("metagenomeSeq")
    require("phyloseq")
    # MGS - A metagenomeSeq object, most-likely produced using the conversion
    # tool make_metagenomeSeq() variable - the variable among the sample_data
    # (aka 'phenoData' in metagenomeSeq) that you want to test physeq - optional
    # phyloseq data object that has the relevant tax_table. phyloseq or
    # taxonomyTable is fine.

    # Create the `mod` variable used in the fitZig test.
    if (inherits(variable, "factor")) {
        # If variable is already a factor, use directly in model.matrix
        mod = model.matrix(~variable)
    } else if (inherits(variable, "matrix")) {
        # If it is a matrix, assume that model.matrix() has been used already
    } else if (inherits(variable, "character")) {
        # If it is a character that specifies a variable in phenoData, use the
        # corresponding variable from MGS
        if (variable %in% colnames(phenoData(MGS)@data)) {
            mod = model.matrix(~phenoData(MGS)@data[, variable])
        } else {
            stop("The phenoData variable name you specified is not present in `phenoData(MGS)`")
        }
    } else {
        stop("Improper specification of the experimental design variable for testing. See `variable` argument")
    }
    # Wrapper to run the Expectation-maximization algorithm and estimate
    # $f_count$ fits with the zero-inflated Guassian (z.i.g.)
    fit = fitZig(MGS, mod)
    # You need to specify all OTUs to get the full table from MRfulltable.
    x = MRfulltable(fit, number = nrow(assayData(MGS)$counts))
    # if any OTUs left out, rm those from x. Detected by NA rownames.
    x = x[!is.na(rownames(x)), ]
    # Modify this data.frame by adding the OTUnames. Clip the ':1' added to the
    # OTU names
    rownames(x) <- gsub(":1", "", x = rownames(x), fixed = TRUE)
    x$OTUnames <- as.character(rownames(x))
    if (!is.null(tax_table(physeq, errorIfNULL = FALSE))) {
        # Attach the bacterial taxonomy to the table, if available
        TAX = data.frame(tax_table(physeq))
        TAX$OTUnames <- as.character(rownames(TAX))
        y = merge(x, TAX, by = "OTUnames")
    } else {
        y = x
    }
    # Sort and return
    y = y[order(y$adjPvalue), ]
    return(y)
}
```

Perform metagenomeSeq differential abundance detection on both the original and rarefied data. The default method for multiple-inference adjustment in `metagenomeSeq::MRfulltable` is Benjamini-Hochberg, the same we have used for other methods in this simulation.

```
MGSreslist <- foreach(physeq = simlist0, .packages = c("phyloseq", "metagenomeSeq")) %dopar% 
    {
        MGSi = make_metagenomeSeq(physeq)
        designfac = factor(gsub("[[:print:]]+\\;", "", sample_names(physeq)))
        y = test_metagenomeSeq(MGSi, designfac)
        rownames(y) <- y[, "OTUnames"]
        # our name convention for B-H adjusted P-value, and OTU-IDs.  Necessary for
        # standardized evaluation of performance
        colnames(y)[colnames(y) == "adjPvalue"] <- "padj"
        colnames(y)[colnames(y) == "OTUnames"] <- "id"
        return(y)
    }
# Test and validate the results using metagenomeSeq on rareified counts
rareMGSreslist <- foreach(physeq = rarelist, .packages = c("phyloseq", "metagenomeSeq")) %dopar% 
    {
        # physeq = rarelist[[3]]
        MGSi = make_metagenomeSeq(physeq)
        designfac = factor(gsub("[[:print:]]+\\;", "", sample_names(physeq)))
        y = test_metagenomeSeq(MGSi, designfac)
        rownames(y) <- y[, "OTUnames"]
        # our name convention for B-H adjusted P-value, and OTU-IDs.  Necessary for
        # standardized evaluation of performance
        colnames(y)[colnames(y) == "adjPvalue"] <- "padj"
        colnames(y)[colnames(y) == "OTUnames"] <- "id"
        return(y)
    }
```

---

## Organize validation results

---

### Summarize results in data.frame

Define function to create a `data.frame` summarizing the differential abundance detection performance, using the `comdelim` variable attributes to label each simulation instance.

```
make_power_df = function(reslist, comdelim, simparamslabels) {
    require("plyr")
    powerdf = ldply(reslist)
    colnames(powerdf)[1] <- "parameters"
    paramdf = ldply(strsplit(powerdf[, "parameters"], comdelim))
    colnames(paramdf) <- simparamslabels
    powerdf = cbind(powerdf, paramdf)
    return(powerdf)
}
```

Define a function for doing this in general, with a few naming conventions in the results table for adjusted P-value (`"padj"`) and OTU ID columns (`"id"`), respectively.

```
eval_res_list = function(resi, NTP) {
    require("ROCR")
    # Some DESeq2 results (for example) had NA adjusted p-values Replace NA
    # $padj values to highest possible value (1.0)
    resi[is.na(resi[, "padj"]), "padj"] <- 1
    # Evaluate detection performance.
    wh.pred = (resi[, "padj"] < 0.05)
    wh.pos = which(wh.pred)
    wh.neg = which(!wh.pred)
    wh.TP = grep("[[:print:]]+\\-TP$", resi[, "id"])
    FPs = sum(!wh.pos %in% wh.TP)
    TPs = sum(wh.pos %in% wh.TP)
    TNs = nrow(resi) - NTP
    FNs = sum(wh.neg %in% wh.TP)
    Power = TPs/NTP
    # Sensitivity: True Positives divided by all positives (sum of true
    # positives and false negatives)
    Sensitivity = TPs/(TPs + FNs)
    # Specificity: True negatives divided by all negatives (sum of true
    # negatives and false positives)
    Specificity = TNs/(TNs + FPs)
    # c(FP=FPs, TP=TPs, Power=Power)
    wh.truth = (1:nrow(resi) %in% wh.TP)
    pred <- prediction(as.numeric(wh.pred), factor(wh.truth))
    # perf <- performance(pred, 'tpr', 'fpr')
    AUC = performance(pred, "auc")@y.values[[1]]
    return(c(FP = FPs, TP = TPs, Power = Power, AUC = AUC, Specificity = Specificity, 
        Sensitivity = Sensitivity))
}
```

## Evaluate differential abundance detection performance

```
# Put every results list into a coherently-named superlist.
superlist = list(DESeq = DESeqreslist, rare_DESeq = rare_DESeqreslist, DESeq2 = DESeq2reslist, 
    rare_DESeq2 = rare_DESeq2reslist, nonorm_mt = nonorm_reslist, rare_mt = raremt_reslist, 
    prop_mt = propmt_reslist, metagenomeSeq = MGSreslist, rare_metagenomeSeq = rareMGSreslist, 
    edgeR = edgeRreslist, rare_edgeR = rare_edgeRreslist)
# Use foreach to organize all this data into a list of data.frames
perfdflist = foreach(resultslist = superlist) %dopar% {
    perflist <- lapply(resultslist, eval_res_list, NTP = nTP)
    if (is.null(names(resultslist))) {
        names(perflist) <- simparams
    } else {
        names(perflist) <- names(resultslist)
    }
    perfdf = make_power_df(perflist, comdelim, simparamslabels)
    return(perfdf)
}
names(perfdflist) <- names(superlist)
df = ldply(perfdflist)
colnames(df)[1] <- "Approach"
# Define whether or not counts were rarefied prior to testing.
df$Normalization <- "Model/None"
df$Normalization[grep("rare_", df$Approach, fixed = TRUE)] <- "Rarefied"
df$Normalization[grep("prop_", df$Approach, fixed = TRUE)] <- "Proportion"
df$Method <- gsub("^[[:alpha:]]+\\_", "", df$Approach)
# Some additional adjustments
df$nsamples <- as.numeric(df$nsamples)
df$EffectSize <- as.numeric(df$EffectSize)
df$FP <- df$FP/(nTP + df$FP)
```

#### Add alpha diversity measures

Calculate alpha diversity indices for every simulation (average across all samples in that experiment).

```
get_alpha = function(physeq) {
    require("phyloseq")
    OTU = otu_table(round(as(otu_table(physeq), "matrix")), FALSE)
    alpha = estimate_richness(OTU, split = FALSE, measures = c("Shannon", "InvSimpson"))
    return(alpha)
}
alphalist <- foreach(parami = df$parameters, .packages = "phyloseq") %dopar% 
    {
        get_alpha(simlist0[[parami]])
    }
alphadf = ldply(alphalist)
df = cbind(df, alphadf)
```

---

## Graphics code. Data organization and graphic specifications.

Calculate means, store as separate `dfmean` variable.

```
nonrepvars = c("Approach", "Normalization", "Method", simparamslabels[!simparamslabels %in% 
    c("Replicate")])
dfmeansd = ddply(df, nonrepvars[nonrepvars != "SampleType"], function(x, vars) {
    xdf = data.frame(x[1, nonrepvars, drop = FALSE], AUC = mean(x$AUC), sd.AUC = sd(x$AUC))
    xdf = cbind(xdf, Shannon = mean(x$Shannon), InvSimpson = mean(x$InvSimpson))
    xdf = cbind(xdf, FP = mean(x$FP), sd.FP = sd(x$FP))
    xdf = cbind(xdf, Power = mean(x$Power), sd.Power = sd(x$Power))
    xdf = cbind(xdf, Sensitivity = mean(x$Sensitivity), sd.Sensitivity = sd(x$Sensitivity))
    xdf = cbind(xdf, Specificity = mean(x$Specificity), sd.Specificity = sd(x$Specificity))
    return(xdf)
}, vars = nonrepvars[nonrepvars != "SampleType"])
maindat = transform(dfmeansd, group = paste0(nsamples, Normalization, Method))
```

Define the library sizes to show in the publication graphic.

```
nreadskeep = c(2000, 50000)
```

#### Main summary figure

PLoS Guidelines:

http://www.plosbiology.org/static/figureGuidelines#dimensions

6.83 width max

9.19in height max

Combine sample types and replicates.

```
pAUCzero = ggplot(dfmeansd, aes(EffectSize, AUC, color = Normalization)) + geom_path(size = 1) + 
    geom_errorbar(aes(ymax = AUC + sd.AUC, ymin = AUC - sd.AUC), width = 0, 
        alpha = 0.5, size = 2) + facet_grid(nreads + nsamples ~ Method) + ggtitle("Differential Abundance Detection Performance") + 
    scale_y_continuous(breaks = c(0.6, 0.8, 1)) + coord_cartesian(ylim = c(0.5, 
    1))
print(pAUCzero)
```

Test further combination

```
nreadskeep = c(2000, 50000)
maindat = transform(dfmeansd, group = paste0(nsamples, Normalization, Method))
pAUCmain = ggplot(maindat, aes(EffectSize, AUC, color = Normalization, alpha = factor(nsamples))) + 
    geom_path(size = 0.5, aes(group = group)) + geom_errorbar(aes(ymax = AUC + 
    sd.AUC, ymin = AUC - sd.AUC), width = 0.2, alpha = 0.25, size = 0.35, position = "dodge") + 
    geom_point(aes(shape = Normalization), size = 1.5) + scale_alpha_discrete(range = c(1, 
    0.4), guide = guide_legend(title = "Samples/Class")) + facet_grid(nreads ~ 
    Method) + scale_y_continuous(breaks = c(0.6, 0.8, 1)) + coord_cartesian(ylim = c(0.5, 
    1)) + theme(text = element_text(size = 8)) + ggtitle("Differential Abundance Detection Performance")
print(pAUCmain)
```

```
# Subset for main figure include in manuscript
pAUCmain$data <- subset(pAUCmain$data, as.numeric(nreads) %in% nreadskeep)
print(pAUCmain)
```

Plot False Positives for supplemental.

```
pDAFP = ggplot(dfmeansd, aes(EffectSize, FP, color = Normalization)) + geom_path(size = 0.5) + 
    geom_errorbar(aes(ymax = FP + sd.FP, ymin = FP - sd.FP), width = 0.2, alpha = 0.25, 
        size = 0.35, position = "dodge") + geom_point(aes(shape = Normalization), 
    size = 1.5) + facet_grid(nreads + nsamples ~ Method) + scale_y_continuous(breaks = c(0.2, 
    0.5, 0.8)) + coord_cartesian(ylim = c(0, 1)) + ylab("False Positive Rate") + 
    ggtitle("Differential Abundance Detection Performance, False Positives")
print(pDAFP)
```

Plot Power for supplemental HTML.

```
pDApower = ggplot(dfmeansd, aes(EffectSize, Power, color = Normalization)) + 
    geom_path(size = 0.5) + geom_errorbar(aes(ymax = Power + sd.Power, ymin = Power - 
    sd.Power), width = 0.2, alpha = 0.25, size = 0.35, position = "dodge") + 
    geom_point(aes(shape = Normalization), size = 1.5) + facet_grid(nreads + 
    nsamples ~ Method) + scale_y_continuous(breaks = c(0.2, 0.5, 0.8)) + coord_cartesian(ylim = c(0, 
    1)) + ylab("Power") + ggtitle("Differential Abundance Detection Performance, Power")
print(pDApower)
```

---

## Combine AUC, Specificity, Sensitivity in one main graphic

Combine overall AUC plot for main manuscript with additional rows summarizing sensitivity and specificity.

I *borrowed* some hints from the `multiplot` function shown in a Cookbook for R page/) about multiple ggplots on the same page/layout/graphic. Those grid-package-requiring lines are toward the bottom. The following puts together the different pieces. Specific plot parameters were chosen by trial and error to most clearly convey the patterns of the results data.

Some additional variable renaming for clarity in the figure.

```
# Set aside a copy that you will modify for plotting
maindat2 = maindat
# Rename the test method by package and function name
maindat2$Method <- gsub("^DESeq$", "'DESeq - nbinomTest'", maindat2$Method)
maindat2$Method <- gsub("^DESeq2$", "'DESeq2 - nbinomWaldTest'", maindat2$Method)
maindat2$Method <- gsub("^edgeR$", "'edgeR - exactTest'", maindat2$Method)
maindat2$Method <- gsub("^metagenomeSeq$", "'metagenomeSeq - fitZig'", maindat2$Method)
maindat2$Method <- gsub("^mt$", "'two sided Welch t-test'", maindat2$Method)
# Rename the library size to include plotmath symbol for clarity
lib_labels = paste0("tilde(N)[L]==", sort(unique(as.integer(maindat2$nreads))))
lib_levels = sort(unique(as.character(maindat2$nreads)))
maindat2$nreads <- factor(maindat2$nreads, levels = lib_levels, labels = lib_labels)
# Re-order the normalization levels
maindat2$Normalization <- factor(maindat2$Normalization, levels = c("Model/None", 
    "Rarefied", "Proportion"))
```

Create and save the figure.

```
pathsize = 0.5
pointsize = 1.5
errorwidth = 0.2
erroralpha = 0.25
errorsize = 0.35
plotlabtextsize = 7
nsampleslegtitle = "Number Samples per Class:"
normlegtitle = "Normalization Method:"
pAUCmain = ggplot(maindat2, aes(EffectSize, AUC, color = Normalization)) + geom_path(size = pathsize, 
    aes(group = group, alpha = factor(nsamples))) + geom_errorbar(aes(ymax = AUC + 
    sd.AUC, ymin = AUC - sd.AUC), width = errorwidth, alpha = erroralpha, size = errorsize, 
    position = "dodge") + geom_point(aes(shape = Normalization), size = pointsize) + 
    facet_grid(nreads ~ Method, labeller = label_parsed) + scale_alpha_discrete(range = c(1, 
    0.4), guide = guide_legend(title = nsampleslegtitle)) + scale_colour_discrete(guide = guide_legend(title = normlegtitle)) + 
    scale_shape_discrete(guide = guide_legend(title = normlegtitle)) + scale_y_continuous(breaks = c(0.6, 
    0.8, 1), limits = c(0.5, 1), oob = function(x, limits) {
    x
}) + coord_cartesian(ylim = c(0.5, 1)) + ggtitle("Differential Abundance Detection Performance (AUC)")
pAUCmain
```

```
# re-plot as function of alpha diversity (Shannon), use full (non-averaged)
# df
pAUCshan = pAUCmain %+% df
pAUCshan$mapping$x <- as.name("Shannon")
pAUCshan$labels$title <- "AUC versus Shannon Index"
pAUCshan$labels$x <- "Shannon Index"
pAUCshan$layers <- pAUCshan$layers[-(1:2)]
pAUCshan
```

```
# Specificity plot
pSpecificity = ggplot(maindat2, aes(EffectSize, Specificity, color = Normalization, 
    alpha = factor(nsamples))) + geom_path(size = pathsize, aes(group = group)) + 
    geom_errorbar(aes(ymax = Specificity + sd.Specificity, ymin = Specificity - 
        sd.Specificity), width = errorwidth, alpha = erroralpha, size = errorsize, 
        position = "dodge") + geom_point(aes(shape = Normalization), size = pointsize) + 
    facet_grid(nreads ~ Method, labeller = label_parsed) + scale_alpha_discrete(range = c(1, 
    0.4), guide = guide_legend(title = nsampleslegtitle)) + scale_colour_discrete(guide = guide_legend(title = normlegtitle)) + 
    scale_shape_discrete(guide = guide_legend(title = normlegtitle)) + scale_y_continuous(breaks = c(0.6, 
    0.8, 1), limits = c(0.5, 1), oob = function(x, limits) {
    x
}) + ggtitle("Differential Abundance Specificity")
pSpecificity
```

```
# re-plot as function of alpha diversity (Shannon Index)
pSpecificityShan = pSpecificity %+% df
pSpecificityShan$mapping$x <- as.name("Shannon")
pSpecificityShan$labels$title <- "Specificity versus Shannon Index"
pSpecificityShan$labels$x <- "Shannon Index"
pSpecificityShan$layers <- pSpecificityShan$layers[-(1:2)]
pSpecificityShan
```

```
# Sensitivity plot
pSensitivity = ggplot(maindat2, aes(EffectSize, Sensitivity, color = Normalization, 
    alpha = factor(nsamples))) + geom_path(size = pathsize, aes(group = group)) + 
    geom_errorbar(aes(ymax = Sensitivity + sd.Sensitivity, ymin = Sensitivity - 
        sd.Sensitivity), width = errorwidth, alpha = erroralpha, size = errorsize, 
        position = "dodge") + geom_point(aes(shape = Normalization), size = pointsize) + 
    facet_grid(nreads ~ Method, labeller = label_parsed) + scale_alpha_discrete(range = c(1, 
    0.4), guide = guide_legend(title = nsampleslegtitle)) + scale_colour_discrete(guide = guide_legend(title = normlegtitle)) + 
    scale_shape_discrete(guide = guide_legend(title = normlegtitle)) + scale_y_continuous(breaks = c(0.2, 
    0.5, 0.8, 1), limits = c(0, 1), oob = function(x, limits) {
    x
}) + xlab("Effect Size") + ggtitle("Differential Abundance Sensitivity")
pSensitivity
```

```
# re-plot as function of alpha diversity (Shannon Index)
pSensitivityShan = pSensitivity %+% df
pSensitivityShan$mapping$x <- as.name("Shannon")
pSensitivityShan$labels$title <- "Sensitivity versus Shannon Index"
pSensitivityShan$labels$x <- "Shannon Index"
pSensitivityShan$layers <- pSensitivityShan$layers[-(1:2)]
pSensitivityShan
```

Now modify these figures for inclusion as main figures in manuscript, and save as PDF version. Create multi-plot grid layout first for HTML supplemental, then to file.

```
# Remove main title
pAUCmain$labels$title <- NULL
pSpecificity$labels$title <- NULL
pSensitivity$labels$title <- NULL
# Remove margins
pAUCmain = pAUCmain + theme(text = element_text(size = plotlabtextsize), plot.margin = unit(c(0, 
    0, 0, 0), "cm"), legend.position = "top", legend.box = "horizontal", legend.margin = unit(-0.5, 
    "cm"))
pSpecificity = pSpecificity + theme(text = element_text(size = plotlabtextsize), 
    plot.margin = unit(c(0, 0, 0, 0), "cm"))
pSensitivity = pSensitivity + theme(text = element_text(size = plotlabtextsize), 
    plot.margin = unit(c(0, 0, 0, 0), "cm"))
# Remove horizontal axis labels from pSpecificity and pAUCmain
pAUCmain = pAUCmain + theme(axis.text.x = element_blank(), axis.title.x = element_blank(), 
    plot.margin = unit(c(0, 0, -0.4, 0), "cm"))
pSpecificity = pSpecificity + theme(axis.text.x = element_blank(), axis.title.x = element_blank(), 
    plot.margin = unit(c(0, 0, -0.4, 0), "cm"))
# Remove redundant x-labels, legends
pAUCmain = pAUCmain + theme(strip.background = element_blank())
pSpecificity = pSpecificity + theme(legend.position = "none", strip.text.x = element_blank(), 
    strip.background = element_blank())
pSensitivity = pSensitivity + theme(legend.position = "none", strip.text.x = element_blank(), 
    strip.background = element_blank())
# Subset for main figure include in manuscript
pAUCmain$data <- pAUCmain$data[(pAUCmain$data$nreads %in% c("tilde(N)[L]==2000", 
    "tilde(N)[L]==50000")), ]
pSpecificity$data <- pSpecificity$data[pSpecificity$data$nreads %in% "tilde(N)[L]==50000", 
    ]
pSensitivity$data <- pSensitivity$data[pSensitivity$data$nreads %in% "tilde(N)[L]==50000", 
    ]
# Create multi-plot grid layout
Layout = grid.layout(3, 1, heights = c(3, 1, 1.2))
grid.show.layout(Layout)
```

```
grid.newpage()
pushViewport(viewport(layout = Layout))
print(pAUCmain, vp = viewport(layout.pos.row = 1, layout.pos.col = 1))
print(pSpecificity, vp = viewport(layout.pos.row = 2, layout.pos.col = 1))
print(pSensitivity, vp = viewport(layout.pos.row = 3, layout.pos.col = 1))
```

Now repeat multi-plot build, but save to file.

```
pdf("Figure_5.pdf", width = 6.8, height = 5)
grid.newpage()
pushViewport(viewport(layout = Layout))
print(pAUCmain, vp = viewport(layout.pos.row = 1, layout.pos.col = 1))
print(pSpecificity, vp = viewport(layout.pos.row = 2, layout.pos.col = 1))
print(pSensitivity, vp = viewport(layout.pos.row = 3, layout.pos.col = 1))
```

---

# Final Save

Stop the cluster and save the environment data.

```
# Stop the cluster
stopCluster(cl)
# Save environemnt
save.image(paste0(savedatafile, "-", datestamp, ".RData"))
```
