# Supplementary figures and images for: Waste Not, Want Not: Why Rarefying Microbiome Data Is Inadmissible
Source: PLoS Comput Biol. 2014 Apr 3;10(4):e1003531. doi: 10.1371/journal.pcbi.1003531 (PMC3974642; doi:10.1371/journal.pcbi.1003531)

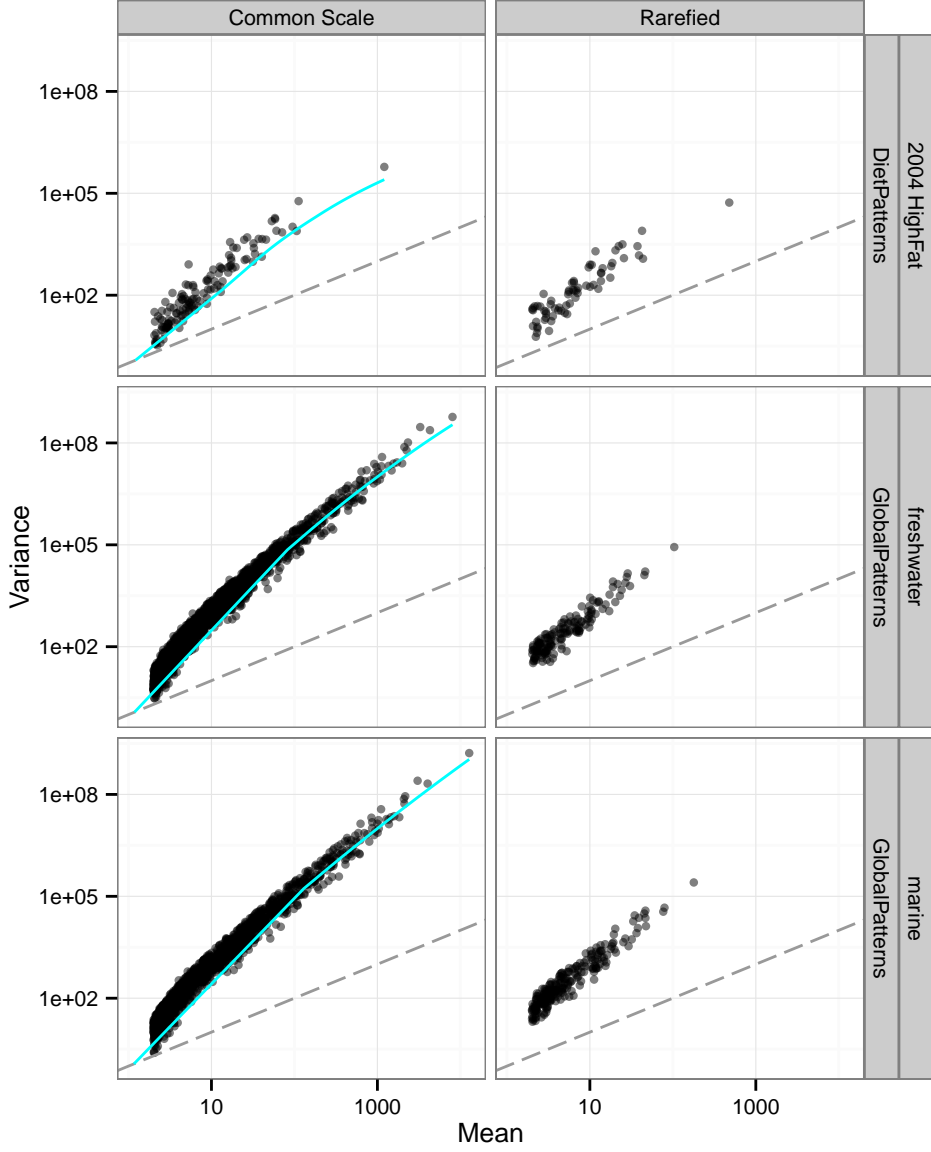

Supplement: Protocol S1 — A zip file containing all supplementary source files. This includes the Rmd source code, HTML output, and all related documentation and code to completely and exactly recreate every results figure in this article. (ZIP) [file pcbi.1003531.s001.zip › norarefy-source/dispersion-survey/microbiome-dispersion-survey.pdf]

Distance Method: —●— Bray-Curtis —▲— PoissonDist —■— top-MSD —✱— UniFrac-u —■— UniFrac-w

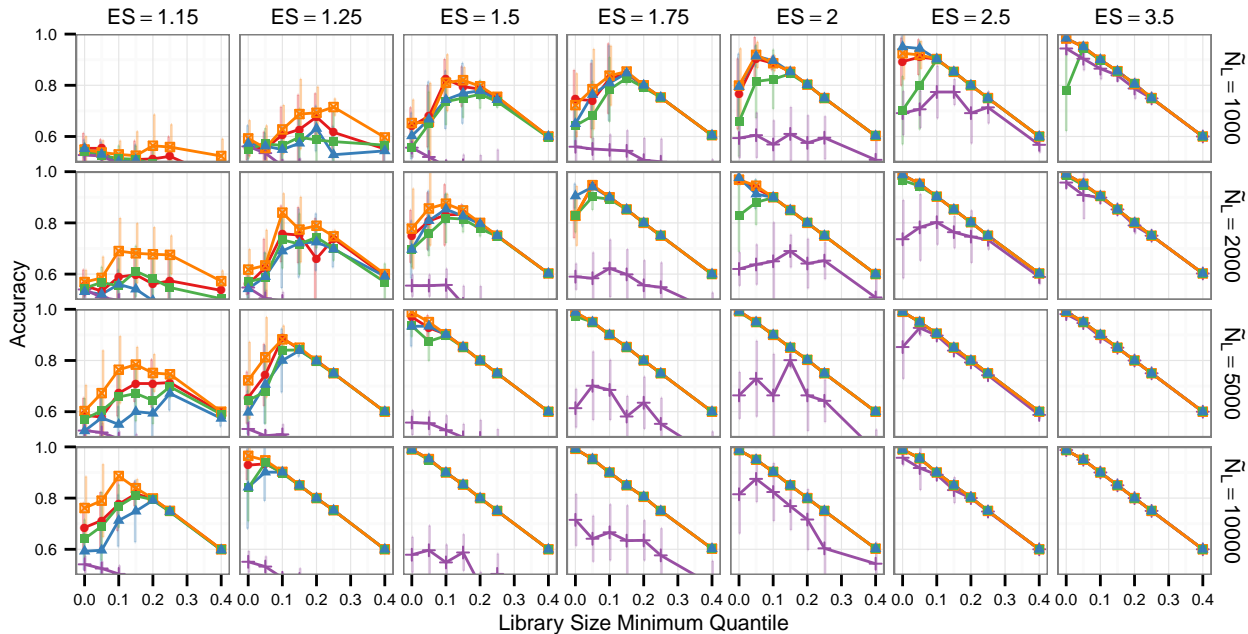

Supplement: Protocol S1 — A zip file containing all supplementary source files. This includes the Rmd source code, HTML output, and all related documentation and code to completely and exactly recreate every results figure in this article. (ZIP) [file pcbi.1003531.s001.zip › norarefy-source/simulation-cluster-accuracy/Figure_4_hclust.pdf]

Distance Method: 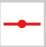 Bray-Curtis 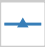 PoissonDist 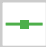 top-MSD 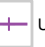 UniFrac-u 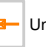 UniFrac-w

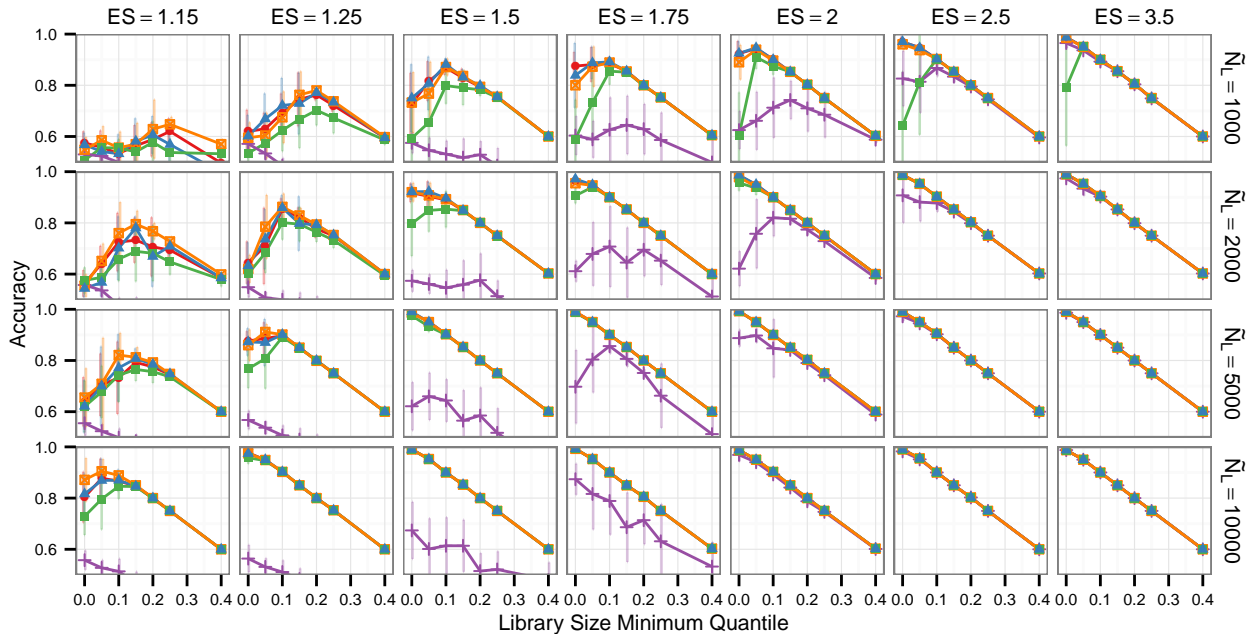

Supplement: Protocol S1 — A zip file containing all supplementary source files. This includes the Rmd source code, HTML output, and all related documentation and code to completely and exactly recreate every results figure in this article. (ZIP) [file pcbi.1003531.s001.zip › norarefy-source/simulation-cluster-accuracy/Figure_4_kmeans.pdf]
