# Supplementary material for: Waste Not, Want Not: Why Rarefying Microbiome Data Is Inadmissible
Source: PLoS Comput Biol. 2014 Apr 3;10(4):e1003531. doi: 10.1371/journal.pcbi.1003531 (PMC3974642; doi:10.1371/journal.pcbi.1003531)
Supplement: Text S1 — A supplemental appendix of the statistical mathematics described in the article. (PDF) [file pcbi.1003531.s002.pdf]

Mathematical Supplement

In this supplementary material we go over some of the statistical details pertaining to the use of hierarchical mixture models such as the Negative Binomial and the Beta Binomial, which are appropriate for addressing additional sources of variability inherent to microbiome experimental data, while still retaining statistical power. We have concentrated our comparison efforts on the Gamma-Poisson mixture model as some authors [1] have remarked that this approach seems to be the most statistically robust approach in the sense that the presence of outliers and model misspecification does not over-perturb the results. We show how a Negative Binomial distribution can occur in different ways leading to different parameterizations. We then show that there are transformations we can apply to these random variables, such that the transformed data have a variance which is much closer to constant than the original. These *variance stabilizing transformations* lead to more efficient estimators and give better decision rules than those obtained via the normalization-through-subsampling method known as *rarefying*.

Two parameterizations of the negative binomial

In classical probability, the negative binomial is often introduced as the distribution of the number of successes in a sequence of Bernoulli trials with probability of success p before the number r failures occur. Thus with the two parameters r and p, the probability distribution for the negative binomial is given as

$$X \sim \mathrm{NB}(r;p)$$

$$\begin{aligned} P(X=k) &= \binom{k+r-1}{k}(1-p)^r p^k \\ &= \frac{\Gamma(k+r)}{k!\Gamma(r)}(1-p)^r p^k \end{aligned}$$

The mean of the distribution is $m = \frac{pr}{1-r}$ and the variance $Var(X) = \frac{pr}{(1-p)^2}$. Sometimes the distribution is given a different parameterization which we use here. This takes as the two parameters: the mean m and $r = \frac{1-p}{p}m$, then the probability mass distribution is rewritten:

$$X \sim \mathrm{NB}(m;r)$$

$$\begin{aligned} P(X=k) &= \binom{k+r-1}{k}\left(\frac{r}{r+m}\right)^r\left(\frac{m}{r+m}\right)^k \\ &= \frac{\Gamma(k+r)}{k!\Gamma(r)}\left(\frac{r}{r+m}\right)^r\left(\frac{m}{r+m}\right)^k \end{aligned}$$

The variance is $Var(X) = \frac{m(m+r)}{r} = m + \frac{m^2}{r}$, we will also use $\phi = \frac{1}{r}$ and call this the overdispersion parameter, giving $Var(X) = m + \phi m^2$. When $\phi = 0$ the distribution of X will be Poisson(m). This is the (mean=m,overdispersion=ϕ) parametrization we will use from now on.

Negative Binomial as a hierarchical mixture for read counts

In biological contexts such as RNA-seq and microbial count data the negative binomial distribution arises as a hierarchical mixture of Poisson distributions. This is due to the fact that if we had technical replicates with the same read counts, we would see Poisson variation with a given mean. However, the variation among biological replicates and library size differences both introduce additional sources of variability.

To address this, we take the means of the Poisson variables to be random variables themselves having a Gamma distribution with (hyper)parameters shape r and scale $p/(1-p)$. We first generate a random mean, λ, for the Poisson from the Gamma, and then a random variable, k, from the Poisson(λ). The marginal distribution is:

$$\begin{aligned} P(X=k) &= \int_0^\infty Po_\lambda(k) \times \gamma_{(r,\frac{p}{1-p})} d\lambda \\ &= \int_0^\infty \frac{\lambda^k}{k!} e^{-\lambda} \times \frac{\lambda^{r-1} e^{-\lambda \frac{1-p}{p}}}{(\frac{p}{1-p})^r \Gamma(r)} d\lambda \\ &= \frac{(1-p)^r}{p^r k! \Gamma(r)} \int_0^\infty \lambda^{r+k-1} e^{-\lambda/p} d\lambda \\ &= \frac{(1-p)^r}{p^r k! \Gamma(r)} p^{r+k} \Gamma(r+k) \\ &= \frac{\Gamma(r+k)}{k! \Gamma(r)} p^k (1-p)^r \end{aligned}$$

Variance Stabilization

Statisticians usually prefer to deal with errors across samples or in regression situations which are independent and identically distributed. In particular there is a strong preference for homoscedasticity (equal variances) across all the noise levels. This is not the case when we have unequal sample sizes and variations in the accuracy across instruments. A standard way of dealing with heteroscedastic noise is to try to decompose the sources of heterogeneity and apply transformations that make the noise variance almost constant. These are called *variance stabilizing transformations*.

Take for instance different Poisson variables with mean μ_i. Their variances are all different if the μ_i are different. However, if the square root transformation is applied to each of the variables, then the transformed variables will have approximately constant variance[1]. More generally, choosing a transformation that makes the variance constant is done by using a Taylor series expansion, called the delta method. We will not give the complete development of variance stabilization in the context of mixtures but point the interested reader to the standard texts in Theoretical statistics such as [2] and one of the original articles on variance stabilization [3]. Anscombe showed that there are several transformations that stabilize the variance of the Negative Binomial depending on the values of the parameters m and r, where $r = \frac{1}{\phi}$, sometimes called the *exponent* of the Negative Binomial. For large m and constant $m\phi$, the transformation

$$\sinh^{-1} \sqrt{\left(\frac{1}{\phi} - \frac{1}{2}\right) \frac{x + \frac{3}{8}}{\frac{1}{\phi} - \frac{3}{4}}}$$

gives a constant variance around $\frac{1}{4}$. Whereas for m large and $\frac{1}{\phi}$ not substantially increasing, the following simpler transformation is preferable

$$\log\left(x + \frac{1}{2\phi}\right)$$

These two transformations are actually used in what is often known as a *generalized logarithmic* transformation applied in microarray variance stabilizing transformations and RNA-seq normalization [4].

Modeling read counts

If we have technical replicates with the same number of reads s_j, we expect to see Poisson variation with mean $\mu = s_j u_i$, for each taxa i whose incidence proportion we denote by u_i. Thus the number of reads for the sample j

[1]Actually if we take the transformation $x \longrightarrow 2\sqrt{x}$ we obtain a variance approximately equal to 1.

and taxa i would be

$$K_{ij} \sim \text{ Poisson } (s_j u_i)$$

We use the notational convention that lower case letters designate fixed or observed values whereas upper case letters designate random variables.

For biological replicates within the same group – such as treatment or control groups or the same environments – the proportions u_i will be variable between samples. A flexible model that works well for this variability is the Gamma distribution, as it has two parameters and can be adapted to many distributional shapes. Call the two parameters r_i and $\frac{p_i}{1-p_i}$. So that U_{ij} the proportion of taxa i in sample j is distributed according to Gamma$(r_i, \frac{p_i}{1-p_i})$. Thus we obtain that the read counts K_{ij} have a Poisson-Gamma mixture of different Poisson variables. As shown above we can use the Negative Binomial with parameters $(m = u_i s_j)$ and ϕ_i as a satisfactory model of the variability.

Now we can add to this model the fact that the samples belong to different conditions such as treatment and control or different environments. This is done by separately estimating the values of the parameters, for each of the different biological replicate conditions/classes. We will use the index c for the different conditions, we then have the counts for the taxa i and sample j in condition c having a Negative Binomial distribution with $m_c = u_{ic} s_j$ and ϕ_{ic} so that the variance is written

$$u_{ic} s_j + \phi_{ic} s_j^2 u_{ic}^2 \qquad (1)$$

We can estimate the parameters u_{ic} and ϕ_{ic} from the data for each OTU and sample condition. This is usually best accomplished by leveraging information across OTUs – taking advantage of a systematic relationship between the observed variance and mean – to obtain high quality shrunken estimates. The end result provides a variance stabilizing transformation of the data that allows a statistically efficient comparisons between conditions. This application of a hierarchical mixture model is very similar to the random effects models used in the context of analysis of variance. A very complete comparison of this particular choice of Gamma-Poisson mixture to the Beta-Binomial and nonparametric approaches can be found in [5].

By comparison, the procedures involving a systematic downsampling (rarefying) are inadmissible in the statistical sense, because there is another procedure that dominates it using a mean squared error loss function. With a Bayesian formalism we can show that the hierarchical Bayes model gives a Bayes rule that is admissible [6].

Other mixture models

If instead of modeling the read counts one uses the proportions as the random variables, with differing variances due to different library sizes, the Beta-Binomial model is the standard approach. This has also been used for RNA-seq data [7] and the package metaStats [8] uses this model although they don't use variance stabilizing transformations of the data.

References

1. Lu J, Tomfohr J, Kepler T (2005) Identifying differential expression in multiple sage libraries: an overdispersed log-linear model approach. BMC bioinformatics 6: 165.

2. Rice JA (2007) Mathematical statistics and data analysis. Cengage Learning.

3. Anscombe FJ (1948) The transformation of poisson, binomial and negative-binomial data. Biometrika 35: 246–254.

4. Anders S, Huber W (2010) Differential expression analysis for sequence count data. Genome biology 11: R106.

5. Yu D, Huber W, Vitek O (2013) Shrinkage estimation of dispersion in Negative Binomial models for RNA-seq experiments with small sample size. Bioinformatics (Oxford, England) 29: 1275–1282.

6. Berger JO (1985) Statistical decision theory and Bayesian analysis. Springer.

7. Zhou YH, Xia K, Wright FA (2011) A powerful and flexible approach to the analysis of RNA sequence count data. Bioinformatics (Oxford, England) 27: 2672–2678.

8. White JR, Nagarajan N, Pop M (2009) Statistical methods for detecting differentially abundant features in clinical metagenomic samples. PLoS computational biology 5: e1000352.
